# Living co-culture fabrication for biologically crosslinked mycelium–cellulose hydrogels and films

**DOI:** 10.1038/s41467-026-76430-6

**Published:** 2026-08-24

**Authors:** Bingyu Xia, Yanpei Tian, Qilong Cheng, Huilong Liu, Xiaojie Liu, Zhenyuan Niu, Xiwei Shan, Pengfei Deng, Wenhui Xu, Shubhra Bansal, Tian Li

**Affiliations:** 1 School of Mechanical Engineering, Purdue University, West Lafayette, IN USA; 2 School of Materials Engineering, Purdue University, West Lafayette, IN USA

**Keywords:** Bioinspired materials, Polymers, Mechanical properties

## Abstract

Living materials, owing to their inherent capacities for growth, self-healing, sensing, and adaptation, have attracted widespread attention in recent years. However, controlling growth of living matter to achieve robust and tunable material properties remains a challenge. Here, we report a co-culture strategy that integrates mycelial microfibers with bacterial cellulose nanofibers into hierarchical hydrogels and transparent films. The biological crosslinking between fungal cell wall mannans and cellulose chains yields an interpenetrating micro–nano network with enhanced interfacial hydrogen bonding. As a result, the films achieve simultaneous high tensile strength (195.62 ± 9.06 MPa) and toughness (11.51 ± 0.94 MJ m^–3^), surpassing most reported biodegradable films. The micro-nano architecture also enables wide-range optical tunability: haze increases from 16.0% to 78.5% while maintaining approximately 80% transparency by controlling culture duration. Mycelium/bacterial cellulose films with tailored properties are promising candidates for applications such as radiative cooling coatings on outdoor displays. This strategy demonstrates a generalizable principle for programming material structure and properties through microbial activity, offering a green pathway toward next-generation sustainable films for transparent radiative cooling, flexible electronics, and optical devices.

## Abstract (web summary)

Microbial growth can be used to create biomaterials, but controlling their growth and material properties is a challenge. Here, the authors co-culture mycelium and bacterial cellulose to create biopolymer composite films with high mechanical and optical properties.

## Introduction

Leveraging microbial activity for material fabrication has emerged as a compelling approach for generating high performance functional systems^1–4^. Different from traditional synthetic materials, engineered living materials exhibit growth-guided multifunctionality, self-healing, sensing, and adaptive capabilities, and are energy efficient and fully biodegradable^5,6^. Moreover, they can also deposit and organize biomaterials across multiple scales, showing great potential for the construction of materials with tunable features^7^. Therefore, exploring how to regulate microbial activities to program material structure and properties represents a highly promising research direction in materials science.

In nature, living organisms exhibit diverse structures and properties under different environmental conditions and growth durations. This principle suggests tunability of living materials can be achieved through controlling the environment (e.g., light, temperature, pH) and growth (nutrient, growth time, culture mode) conditions of living microorganisms^8–12^. Among microbial systems, bacterial cellulose (BC) produced extracellularly by *Komagataeibacter xylinus* has received considerable attention due to its high crystallinity, purity, and tensile strength, presenting potential applications in electronics, packaging and biomedical engineering^13–16^. However, despite high tensile strength, natural BC suffers poor stretchability due to its highly crystalline nanofiber network^17–20^. Besides, BC pellicles formed at air-liquid surface exhibit a uniform structure with limited tunability. These inherent disadvantages hinder the application of BC in a broader range of scenarios.

**Fig. 1:**
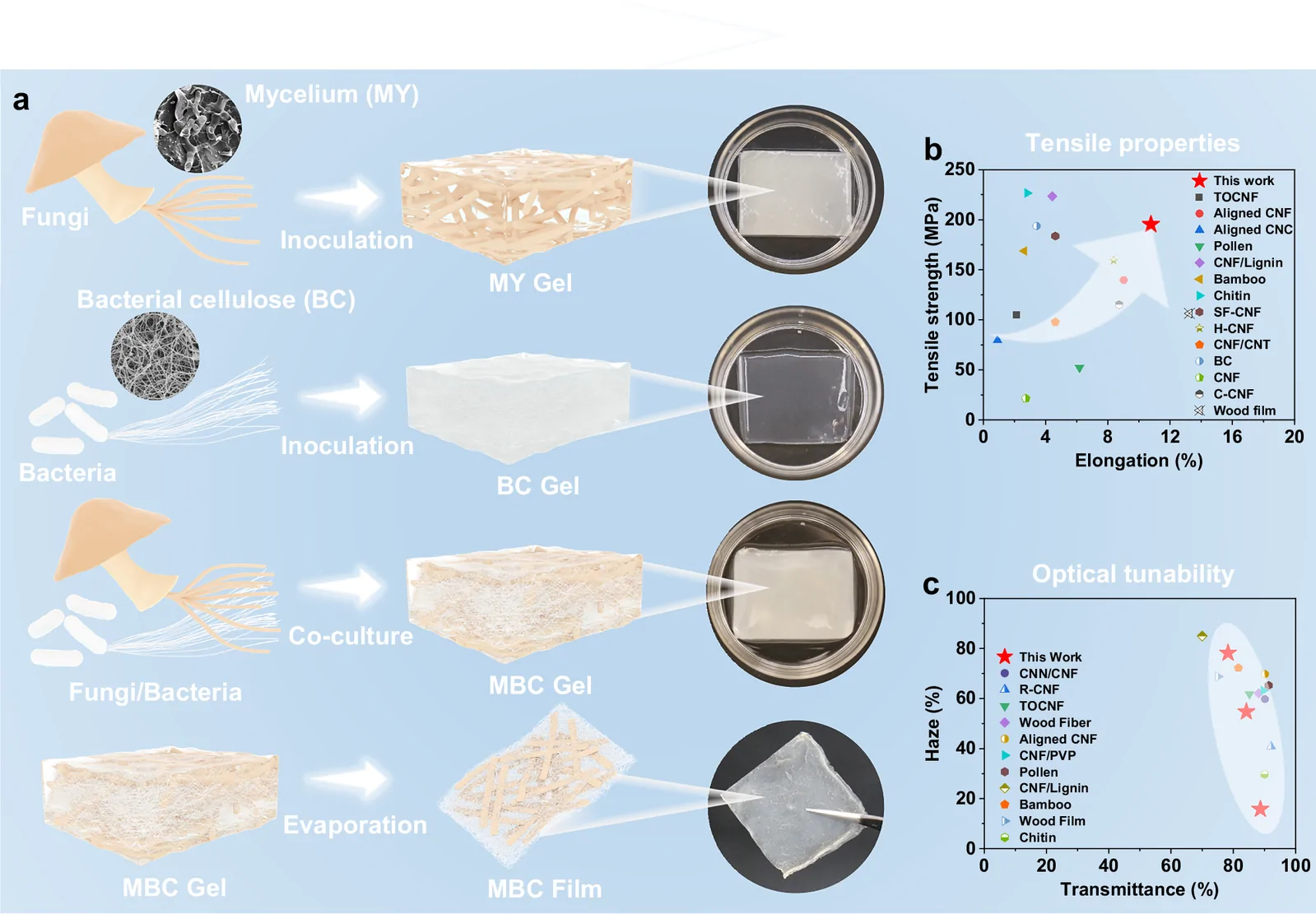
Fabrication and properties of MBC hydrogels and films. **a** Schematic diagram of the fabrication of bacterial cellulose (BC), mycelium (MY) and mycelium/bacterial cellulose (MBC) hydrogels and MBC films. **b**, **c** Comparison of mechanical and optical properties of MBC films with other biodegradable films. Abbreviations and corresponding references are provided in Supplementary Table 1.

Constructing hierarchical structures provides a strategy to overcome such limitations by enhancing material performance and tunability^21,22^. For example, nacre (mother-of-pearl, a natural hierarchical composite composed of aragonite platelets and organic interfaces) exhibits superior strength and toughness through a “brick-and-mortar” structure^23,24^, butterflies show beautiful tunable structural colors arising from the hierarchical structured wings^25^. Here, we introduce a living co-culture method that exploits the simultaneous growth of microscale fungal mycelium (MY) and nanoscale BC fibers in a designed liquid medium to form hierarchically structured hydrogels through biological crosslinking (i.e., the combined effect of physical entanglement and interfacial hydrogen bonding between mycelial hyphae and BC nanofibers) (Fig. 1a), without chemical additives or external processing. Through strain screening, we found that *Phanerochaete chrysosporium* (PC) forms only submerged hyphae in liquid medium without developing a white aerial hyphal layer within the desired culture duration (Supplementary Fig. 1). This growth behavior differs from previously reported bilayer mycelium hydrogels and provides a suitable structure for co-culturing with BC and regulating the optical properties of the resulting films^26^. The mycelium/bacterial cellulose (MBC) films exhibit a hierarchical micro-nano architecture that overcomes the conventional trade-off between strength and toughness through efficient load transfer and crack energy dissipation across multiscale fiber networks (Fig. 1b). Furthermore, MBC films also possess high haze due to the refractive index mismatch among BC, mycelium and interlamellar pores. By adjusting culture duration, we achieve haze values ranging from 16.0 to 78.5% at ~80% transparency, a property combination not accessible in natural or synthetic biofilms (Fig. 1c). The MBC films with engineered optical and mechanical properties were further coated onto glass and flexible substrates, exhibiting effective radiative cooling performance and outdoor display capability. This biologically driven method enables direct control of microstructure and macroscopic properties, establishing microbial regulation as a general principle for designing engineering materials.

## Results

### Morphology and chemical interactions between mycelium and BC

Mycelium is mainly composed of micron-sized tubular filaments termed hyphae, which are formed by linearly connected fungal cells and function as the main structures for growth and nutrient absorption (Supplementary Fig. 2)^27^. These cells are enclosed by cell walls, composed of mannan, glucan, chitin and mannoproteins, providing mechanical strength and reactive sites^28^. In liquid culture, we found that mycelium can independently form a hydrogel. As fungal spores germinate and hyphae branch, they develop into a three-dimensional entangled microfiber network with inherent porosity (Supplementary Fig. 3). By contrast, bacterial cellulose produced extracellularly by *Komagataeibacter xylinus*, self-assembles through hydrogen bonding into a highly crystalline nanofibrous hydrogel, consistent with previous reports (Supplementary Fig. 4). To enable the simultaneous growth of mycelium and BC, we designed a composite LM-HS medium that provides the nutrients required for both mycelial growth and BC biosynthesis. The presence of readily metabolizable glucose is known to repress the cellulase-related gene expression in white-rot fungi, including PC^29,30^, while the high crystallinity of BC further limits its enzymatic degradation^31,32^, allowing the BC network to remain largely preserved during the co-culture process. During the co-culture process, hyphae elongate primarily at their tips through continuous cell wall synthesis and vesicle-mediated transport^33^. At the same time, bacterial cells continuously synthesize and secrete cellulose nanofibers into the extracellular space. As hyphae extend and branch, these cellulose fibers are deposited between and around the growing hyphae, leading to an interpenetrating three-dimensional network stabilized by hydrogen bonding and physical interlocking.

**Fig. 2:**
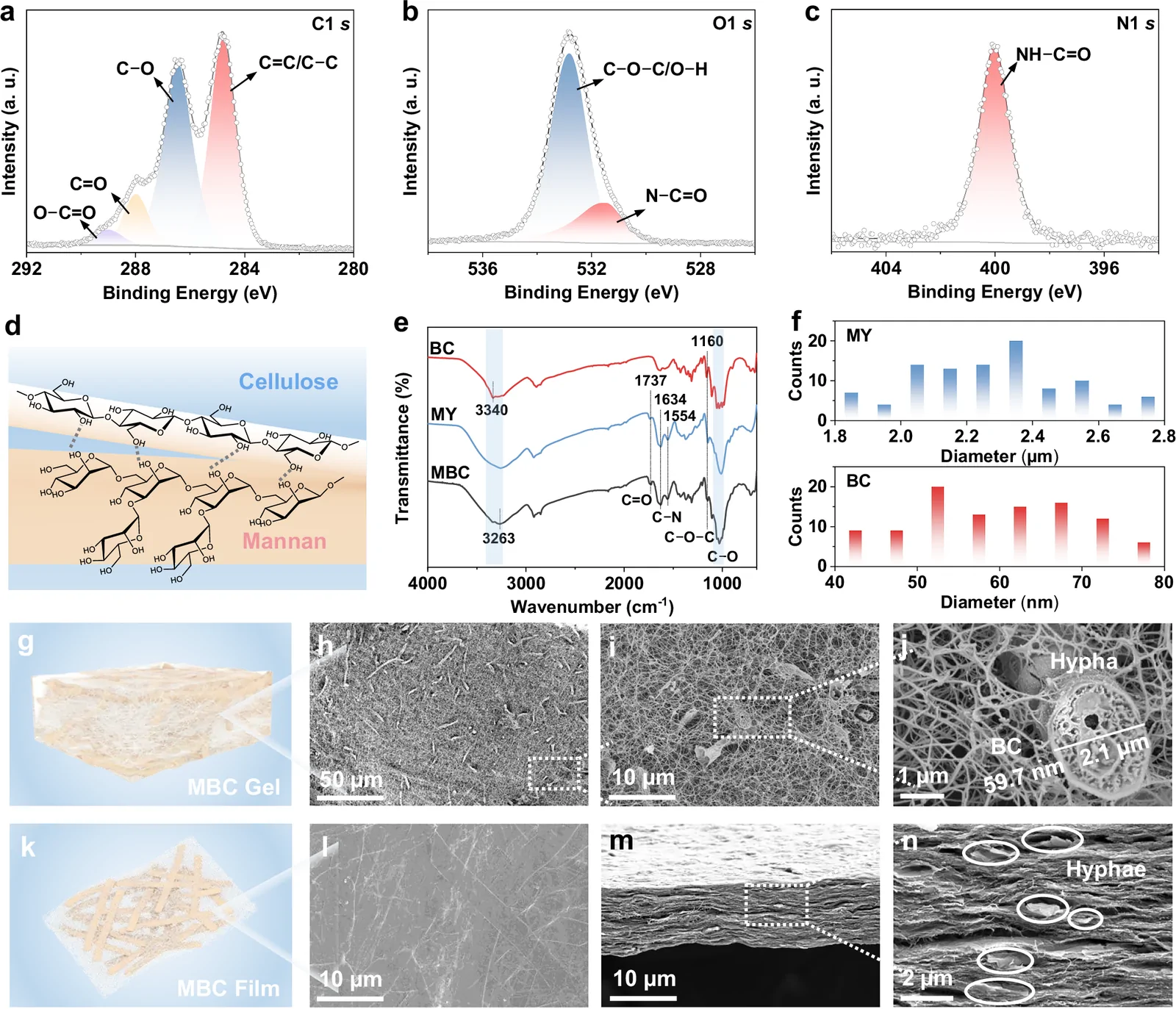
Structure of MBC hydrogel and MBC film. **a**–**c** C 1*s*, O 1*s* and N 1*s* X-ray photoelectron spectroscopy (XPS) spectra of pure mycelium film. **d** Schematic illustration of hydrogen bonding between mannan on mycelial hyphae and cellulose chains of BC. **e** Attenuated total reflectance Fourier transform infrared spectroscopy (ATR-FTIR) of BC, MY and MBC films. **f** Diameter distribution of MY and BC. **g** Schematic diagram of MBC hydrogel. **h**–**j** Cross-sectional cryo-scanning electron microscopy (cryo-SEM) images of MBC hydrogel. **k** Schematic diagram of MBC film. **l** Surface SEM images of MBC film. **m**, **n** Cross-sectional SEM images of MBC films.

X-ray photoelectron spectroscopy (XPS) analysis was carried out to characterize the surface composition of mycelial hyphae. The XPS spectrum of the pure mycelium film shows strong C 1*s* and O 1*s* peaks, along with a weak N 1*s* peak (Supplementary Fig. 5). The C 1*s* spectrum can be divided into four peaks at 284.80, 286.45, 287.95, and 289.05 eV, corresponding to C–C/C=C, C–O, C=O, and O–C=O, respectively (Fig. 2a). These peaks are consistent with the polysaccharide-rich cell wall structure of mycelium, including mannans and mannoproteins, as well as chitin-associated carbonyl groups^34^. In the O 1*s* spectrum, peaks at 531.50 and 532.85 eV are assigned to N–C=O and C–O–C/O–H groups, respectively (Fig. 2b). The N 1*s* spectrum displays a single peak at 400.00 eV, mainly arising from the amide groups (HN–C=O) of chitin (Fig. 2c)^35^. These results indicate that mannans and chitin on mycelial cell walls are rich in hydroxyl and amide groups, which can establish hydrogen bonds with the hydroxyl groups of bacterial cellulose (Fig. 2d). These interfacial interactions not only stabilize the junctions between mycelial hyphae and BC nanofibers but also contribute to the formation of an interpenetrating micro–nano fiber network, providing the structural basis for the hierarchical architecture of MBC hydrogels^36^. As shown in attenuated total reflectance Fourier transform infrared spectroscopy (ATR-FTIR) spectrum, BC exhibits a broad peak centered at 3340 cm^–1^, corresponding to the O–H stretching vibration, shifting to 3263 cm^–1^ in the MBC sample. This red shift indicates enhanced hydrogen bonding between BC and mycelial hyphae due to interfacial interactions^21^. Besides, both MY and MBC films show peaks at 1737 cm^–1^ (C=O stretching), 1634 cm^–1^ (amide I), and 1554 cm^–1^ (amide II), which are primarily associated with protein and polysaccharide components from mycelium (Fig. 2e). These peaks are absent in pure BC^37^, indicating successful incorporation of MY in BC. The peaks at 1160 cm^–1^ and 1050–1020 cm^–1^, attributed to C–O–C and C–O stretching vibrations of cellulose are retained in MBC films, confirming the preserved cellulose backbone structure^38^. These results demonstrate that the co-culture LM-HS medium successfully enabled the coexistence and integration of mycelium and bacterial cellulose within a unified composite matrix.

MBC hydrogel presents a self-supporting and hierarchical structure of microfibers and nanofibers. Diameter analysis reveals that mycelium is composed of micron-sized hyphae with a diameter of 2.3 ± 0.2 µm, while BC consists of nanofibers with a diameter of 59.5 ± 9.9 nm (Fig. 2f). The interpenetrating micro-nano fiber network stabilized by strengthened interfacial hydrogen bonding provides a structural basis for the tunable properties. Scanning electron microscopy (SEM) images show the intertwined fibers forming a uniform double network (Fig. 2g–j), in which BC nanofibers and hyphae are tightly bound via interfacial hydrogen bonding. Upon drying, the MBC hydrogel transforms into a flat film with a layer-stacked structure composed of hyphae and BC nanofibers (Fig. 2k–n)^33^. The top surface (Fig. 2l) exhibits a combination of microscale hyphae with a ribbon-like morphology and nanoscale fibrillar BC networks. The initially tubular hyphae collapsed into flattened, ribbon-like filaments due to the loss of turgor pressure, resulting in the formation of interlamellar pores within the MBC films (Fig. 2n). Furthermore, evaporation-induced consolidation reduced inter-fiber spacing and brought hydroxyl-rich chains into denser compaction, which further enhanced the interfacial interaction via hydrogen bonding. The biodegradability of the MBC film was also evaluated through a burial test. As shown in Supplementary Fig. 6, the MBC film degrades into small fragments after 4 weeks of soil burial, accompanied by an ~75% area loss, indicating its good biodegradability.

### Mechanical properties of MBC hydrogels and films

**Fig. 3:**
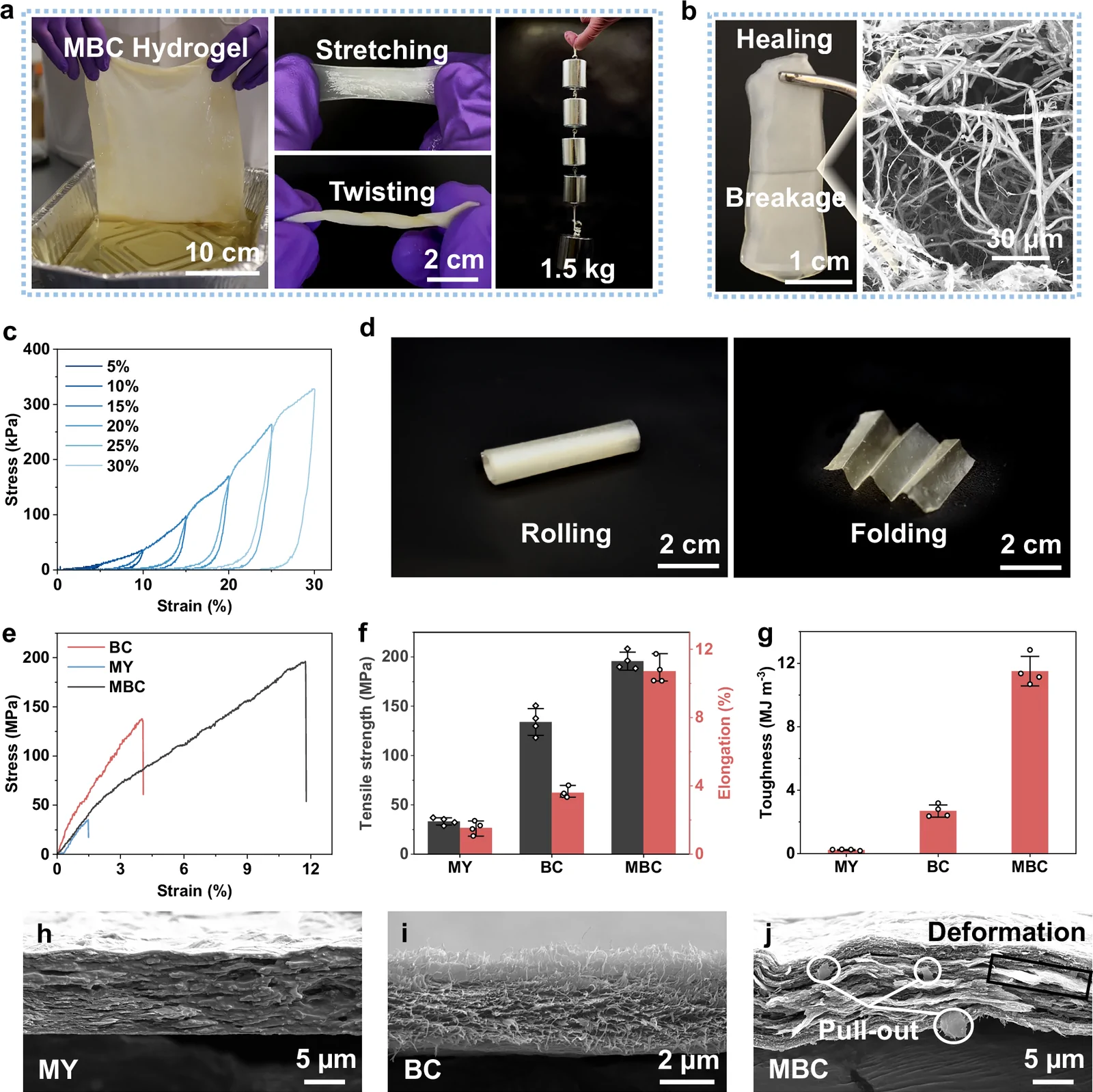
Mechanical properties of MBC hydrogels and films. **a** Photographs showing MBC hydrogels and their tensile properties. **b** Photographs of self-healing behavior of MBC hydrogels and SEM images of the breakage. **c** Loading-unloading cyclic tensile stress-strain curves of MBC hydrogel. **d** Photographs of the rolled and folded MBC films. **e** Stress-strain curves of MY, BC and MBC films. **f** Comparison of the tensile strength and elongation of MY, BC and MBC films. **g** Comparison of the toughness of MY, BC and MBC films. **h**–**j** SEM images of tensile fracture surfaces of MY, BC and MBC films. Fiber pull-out is marked by white circles, and plastic deformation is highlighted by black rectangles. All error bars represent standard deviation, *n* = 4 independently grown samples.

Benefiting from its hierarchical structure and strong hydrogen bonding between mycelium and BC, MBC hydrogel can be stretched, twisted, and can sustain a 1.5 kg load without rupture (Fig. 3a). The tensile strength and elongation of the MBC hydrogel are 1.27 ± 0.12 MPa and 48.47 ± 1.48%, respectively (Supplementary Fig. 7). In addition, the hydrogel also exhibits self-healing properties as the 2 cut pieces rejoined after 12 h (Fig. 3b). When the fractured surfaces were brought back into contact, hyphae and BC re-entangled at interface and hydrogen bonds between them were re-established. These interactions enable the reconstruction of the interfacial hydrogen bonding and restore the mechanical integrity of the MBC film, resulting in the observed self-healing behavior^39,40^. SEM image of the fractured region after an additional 24 h of healing shows that the continued growth of hyphae and BC forms new entanglements, further contributing to the repair of the damaged region^41^. As indicated in Supplementary Fig. 8, both the microscale hyphae and nanoscale BC in the MBC hydrogel continue to develop after being cut, forming a new hierarchical thin layer. Cyclic tensile test demonstrates that the MBC hydrogel withstands strains up to 30% without rupture and displays distinct hysteresis (Supplementary Fig. 9). The hydrogel exhibits ~80% residual strain after unloading (Fig. 3c), which arises from the disentanglement and reorientation of nano- and microfibers, accompanied by the disruption and reformation of hydrogen bonding networks^42,43^. The reorientation of nano- and microfibers at 30% strain was further confirmed by the anisotropic small-angle X-ray scattering (SAXS) pattern perpendicular to the loading direction (Supplementary Fig. 10)^44^. The synergistic effects of fiber reorientation and reversible hydrogen-bonding dynamics endow the MBC hydrogel with high stretchability and energy dissipation capability, which in turn accounts for the high toughness of the corresponding MBC films.

After air drying, the MBC film is transparent and highly processable, which can be rolled into tubes and folded into corrugations without cracking (Fig. 3d), making it attractive for optoelectronic devices and light-management films. Stress-strain curves of the MBC films indicate superior mechanical properties compared to pure MY and BC films (Fig. 3e), which can be attributed to its hierarchical structure and interfacial hydrogen bonding between hyphae and BC. As shown in Fig. 3f, g, the average tensile strength of MBC films is 195.62 ± 9.06 MPa which is 1.5 times higher than that of pure BC films (134.01 ± 13.63 MPa) and 5.9 times higher than that of MY films (33.18 ± 3.65 MPa). Moreover, MBC films also exhibit significantly improved toughness (11.51 ± 0.94 MJ m^–3^) when compared to pure BC (2.69 ± 0.39 MJ m^–3^) and MY (0.23 ± 0.04 MJ m^–3^) films. The tensile strength and toughness of MBC films are superior to most of the biomass films, such as CNF, wood and chitin (Supplementary Table 1). Cross-sectional SEM images of broken films were used to analyze the fracture behavior of the films. Both MY and BC films exhibit single-length-scale layer structure (Fig. 3h, i), whereas MBC film displays a multiscale lamellar structure consisting of micro-hyphae and BC nanofibers (Fig. 3j). The MY film exhibits a flat fracture surface without deformation and hypha pull-out, indicative of brittle fracture. The BC film shows nanofiber pull-out with short pull-out lengths and slight deformation, reflecting constrained plasticity. In comparison, the MBC film reveals significant layer deformation together with elongated and pulled-out hyphae and nanofibers, evidencing a plastic and highly dissipative failure mode. In comparison with pure MY and BC films, which show brittle fractures with elongations of only 1.54 ± 0.37% and 3.59 ± 0.30%, MBC film demonstrates a significantly enhanced elongation of 10.72 ± 0.75%, nearly 7 times higher than MY film and 3 times higher than BC film. High-magnification images further reveal that the pulled-out hyphae are encapsulated by BC nanofibers (Supplementary Fig. 11a), consistent with a strong hydrogen-bonding network that increases the interfacial adhesion and restricts relative sliding at the fiber interface^45,46^. The incorporation of mycelium into BC matrix facilitates efficient stress transfer and increases the energy dissipation of MBC films under tensile loading.

When tensile loading was applied to the MBC film, BC nanofibers were stretched and aligned along the load direction (Supplementary Fig. 11a). Hyphae encapsulated by nanofibers were also slightly stretched, followed by interlamellar shear between adjacent BC layers (Supplementary Fig. 11b). As the load increased, partial fracture of nanofibers occurs, initiating microcracks. Relative sliding between nanofibers and hyphae then occurred, but this motion was restricted by reversible interfacial hydrogen bonds, which deflected the cracks and constrained their propagation (Fig. 3j). At higher strains, the hyphae fractured, while adjacent BC nanofibers were pulled out from the matrix. This multistep fracture pathway, involving fiber stretching, shear deformation, crack deflection, and fiber pull-out, enabled the MBC film to simultaneously achieve high strength (195.62 ± 9.06 MPa) and toughness (11.51 ± 0.94 MJ m^–3^), helping overcome the conventional strength–toughness trade-off through an interpenetrating micro-nano hierarchical structure built by microbial growth.

### Tunable optical properties and application of MBC films

**Fig. 4:**
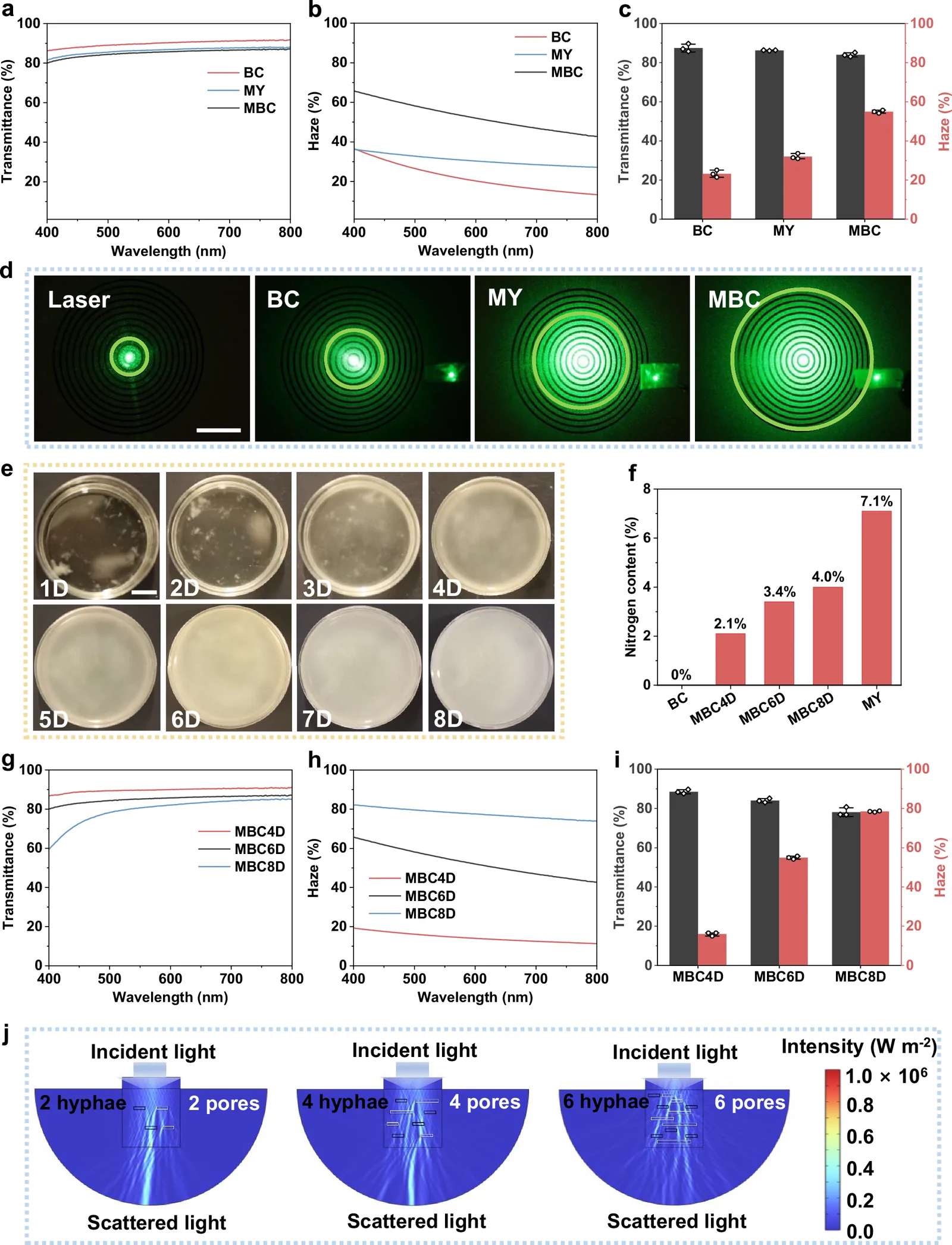
Tunable optical properties of MBC films. **a**, **b** Transmittance and haze ultraviolet-visible (UV–vis) spectra (400–800 nm) of BC, MY and MBC films cultured for 6 days. **c** Transmittance and haze comparison (550 nm) of BC, MY and MBC films. **d** Photographs of laser spots before and after being scattered by BC, MY and MBC films. Scale bar: 4 cm. **e** Growth process of co-cultured fungi and bacteria over 8 days (D denotes days). Scale bar: 1 cm. **f** Nitrogen content of BC, MY, and MBC films with different culture durations determined by energy-dispersive X-ray spectroscopy (EDS). (MBC4D, 4 days; MBC6D, 6 days; MBC8D, 8 days). **g**, **h** Transmittance and haze UV–vis spectra (400–800 nm) of MBC films with different culture duration (4 days, 6 days and 8 days). **i** Transmittance and haze comparison (550 nm) of MBC films. **j** Optical simulation of light scattering through MBC films with different mycelial content. The background is BC matrix; the black and white boxes represent hyphae and interlamellar air pores in the matrix. All error bars represent standard deviation, *n* = 3 independently grown samples.

The optical properties of MBC films were evaluated using an ultraviolet-visible (UV–vis) spectrophotometer. The BC, MY, and MBC films block 41.6%, 61.0%, and 56.8% of UV light, respectively (Supplementary Fig. 12), owing to the presence of melanins, carotenoids, and mycosporine-like amino acids that strongly absorb UV-A and UV-B radiation (280–400 nm)^47^. At 550 nm, pure BC films exhibit the highest transmittance (87.5%) but the lowest haze (23.2%), reflecting weak light scattering due to their uniform nanofiber network (Fig. 4a–c). By contrast, MBC film maintains a comparably high transmittance of 84.0% while achieving a significantly higher haze of 54.9% than both MY (32.0%) and BC films (23.2%), owing to its hierarchical structure. This demonstrates that the hierarchical integration of micro- and nanofibers in MBC films significantly enhances light scattering without severely compromising transparency. Light scattering effect was further visualized by a green laser spot test (Fig. 4d). As the beam passed through the films, the spot expanded into a luminous area proportional to the scattering intensity. MBC films produce the largest scattering pattern, followed by MY and then BC films, confirming that the hierarchical micro–nano architecture of MBC imparts the strongest light-scattering capability.

The increased haze of MBC films is mainly attributed to the mismatch of refractive index (RI) induced by hierarchical micro-nano structure. As given by Supplementary Note 1, the reflection coefficient *Г* increases with the RI contrast between neighboring components, leading to stronger interfacial reflection within the film. The pure BC film features a densely packed nanofibrous network with fiber diameters (59.5 ± 9.9 nm) much smaller than the wavelength of visible light (400–800 nm) (Supplementary Fig. 13a), leading to weak and small-angle Rayleigh scattering (*Q*_sca_ ∝ (*r*/*λ*)^4^, Supplementary Note 2). In pure MY films, the diameters of hyphae are comparable to visible wavelengths, giving rise to Mie scattering. The Mie scattering efficiency, as expressed in Supplementary Note 3 and 4, increases with the RI contrast between the fibers and the surrounding medium^48^. However, as observed in the cross-sectional SEM images, the MY film exhibits a uniform and dense morphology with minimal RI difference (Supplementary Fig. 13b), leading to low scattering efficiency. In contrast, the MBC film possesses a hierarchical structure composed of micro- and nanofibers with a RI contrast of 0.15 between the hyphae (RI = 1.41) and BC nanofibers (RI = 1.56)^49,50^. Moreover, interlamellar pores were formed when the tubular hyphae collapsed during the evaporation of the MBC hydrogel, resulting in a larger RI contrast of 0.56 between air (RI = 1.00) inside the pores and the BC nanofiber network. These multiscale RI mismatches induce multidirectional reflections and refractions at the interfaces among different components, thereby enhancing the overall Mie scattering within the MBC film.

The ability to tune optical haze while maintaining high transparency is highly desirable for optoelectronic films, as it allows light-scattering performance to be tailored for specific applications. In MBC films, this tunability is achieved through their hierarchical architecture, where mycelial microfibers interpenetrate BC nanofibers. By varying culture duration, the balance between these two components can be adjusted. The growth of BC, MY, and MBC hydrogels was monitored for 8 days. BC produced by *Komagataeibacter xylinus* formed a transparent nanofiber network after 3 days and a visible pellicle at the air-liquid interface after 5 days (Supplementary Fig. 14a). In parallel, hyphae growth initiated with spore germination and branching during the first 3 days, giving rise to a visible white gel by day 4 (Supplementary Fig. 14b). In the co-culture process, BC network formation coincided with hyphae germination, generating a double network stabilized by hydrogen bonding (Fig. 4e). By day 4, the liquid surface was covered with intertwined hyphae embedded in BC, and extended culture led to a progressively denser and more uniform hybrid hydrogel. Energy-dispersive X-ray spectroscopy (EDS) was applied to evaluate changes in mycelial content within the MBC films based on the nitrogen signal, since BC contains no nitrogen, whereas mycelium contains nitrogen-rich components such as chitin and proteins. As shown in Fig. 4f and Supplementary Fig. 15, the pure BC film exhibits no detectable nitrogen signal, while the pure MY film contains 7.1% nitrogen. In comparison, the MBC samples cultured for 4, 6 and 8 days show nitrogen contents of 2.1%, 3.4% and 4.0%, respectively. Although EDS cannot accurately quantify the mycelial content, the increasing nitrogen signal suggests a greater mycelial contribution at longer culture durations. Thermogravimetric analysis (TGA) also provides supporting evidence for the trend of increased mycelial contribution indicated by the EDS analysis, although it cannot directly quantify the mycelial content. Pure BC films show the greatest weight loss (83.9%) between 200 and 550 °C, leaving only 8.2% residue at 550 °C (Supplementary Fig. 16a). The weight loss of MBC films between 200 and 550 °C decreases from 73.8% to 64.9%, while the char yield at 550 °C increases from 15.2% to 26.0% with prolonged culture duration (Supplementary Fig. 16b–d). The increased char yield is mainly attributed to the greater contribution of mycelial components, such as chitin, proteins, and lipids, which tend to form more residual char during thermal degradation^51,52^.

The structural tunability of MBC films can be directly reflected in their optical performance. UV–vis measurements show that the haze of MBC films at 550 nm increases from 16.0% (4 days) to 54.9% (6 days) and 78.5% (8 days), while transmittance remains relatively high, only decreasing modestly from 88.5% to 78.1% (Fig. 4g–i). Compared with other biodegradable films (Supplementary Table 2), MBC films offer a wider range of haze tunability at high transparency, which can be attributed to the controllable mycelial content within the films. The increased mycelial hyphae with increasing culture duration introduces more interlamellar air pores into the MBC films. The estimated pore area fraction in the film cross-section increases from 5.02% to 11.85% with prolonged culture duration (Supplementary Fig. 17). Although the presence of wrinkles may affect the accuracy of the porosity measurement, the analysis still suggests a general increase in pore content within the films. The increased number of macropores and mycelial hyphae provides additional light-scattering interfaces due to the RI mismatch among air, mycelium, and BC. Optical simulation further shows that the incident light underwent multidirectional reflection and refraction when encountering the interfaces among BC nanofibers, hyphae, and air pores (Fig. 4j). The film thickness increases from 7.85 ± 0.22 µm at 4 days to 9.10 ± 0.28 µm at 6 days and 9.64 ± 0.44 µm at 8 days, which further promotes light scattering by extending the optical path length within the hierarchical network (Supplementary Fig. 18). In addition, the root mean square (RMS) roughness values for films cultured for 4, 6, and 8 days were 37.18, 37.60, and 44.34 nm, respectively (Supplementary Fig. 19). Although a slight increase is observed over the culture period, all values remain substantially below the wavelength range of visible light (400–800 nm), indicating that the surface of MBC films is optically smooth and the roughness contributes negligibly to light scattering (Supplementary Note 5)^53^. By simply adjusting culture duration, MBC films achieve tunable optical haze across a wide range while preserving high transmittance, offering a scalable, bio-based strategy for tailoring light scattering in applications.

**Fig. 5:**
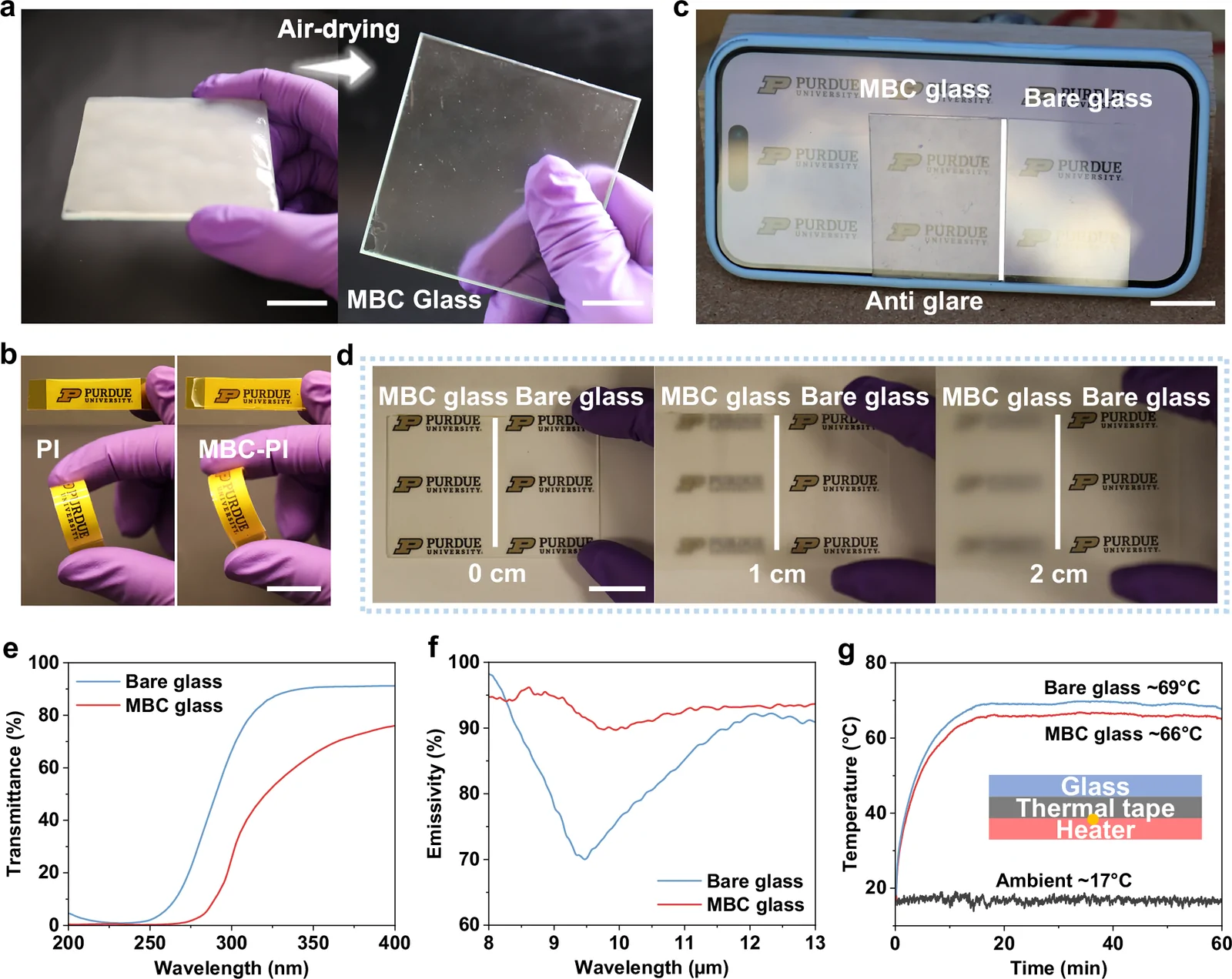
Application of MBC glass for transparent radiative cooling displays. **a** Preparation of MBC glass by air-drying the MBC hydrogel on glass. **b** MBC coating on flexible polyimide (PI) films. **c** Outdoor anti-glare performance of MBC glass and bare glass on the screen of smart phone. **d** Photographs showing the view of pattern under MBC glass and bare glass at different viewing distances (0 cm, 1 cm, 2 cm). **e** Transmittance spectra of bare glass and MBC glass in the ultraviolet region (200–400 nm). **f** Emissivity in the atmospheric window (8–13 μm) of bare glass and MBC glass. **g** Temperature of heater covered by bare glass and MBC glass under solar illumination. The orange circle shows the position of thermocouple. Scale bars: 2 cm.

Through microbial regulation of the hierarchical structure, MBC films combining simultaneously high transparency, high haze, and enhanced mechanical properties were obtained. These combined features make them suitable for outdoor radiative cooling displays. Therefore, MBC hydrogels were air-dried on various substrates (e.g., glass, polyimide) to form an adherent coating, in which the hierarchical BC nanofibers and hyphae established hydrogen bonding with the substrate surfaces, ensuring robust interfacial adhesion (Fig. 5a, b). Owing to its high tensile strength and toughness, the MBC composite can be uniformly coated on flexible polyimide (PI) films without cracking upon bending (Fig. 5b). The resulting MBC coating exhibits both good display performance and effective radiative cooling functionality.

In terms of display performance, the high optical haze of the MBC coating provides effective anti-glare capability, preserving display readability under strong illumination (Fig. 5c). Meanwhile, display-visibility tests reveal that the MBC glass maintains good display readability nearly identical to that of bare glass when placed directly on patterns (Fig. 5d). As the distance increases, images beneath glass remain sharp, while those beneath MBC films progressively blurred due to the enhanced light scattering. Notably, MY films exhibit a similar scattering behavior to MBC films, whereas BC films preserve image clarity even at 2 cm, confirming their weak scattering (Supplementary Fig. 20). Compared with the free-standing MBC film, the MBC glass shows better visibility, which may be attributed to hydrogen-bond-assisted adhesion between the coating and the glass substrate that restricts drying-induced deformation and results in a flatter surface morphology^54^. Moreover, the MBC glass achieves a UV-blocking efficiency of 68.0%, which is 38.5% higher than that of bare glass, contributing to improved durability of outdoor display modules (Fig. 5e). Regarding its radiative cooling property, the MBC glass exhibits an improved mid-infrared emissivity of ~93% within the atmospheric window (8–13 µm), compared with bare glass (Fig. 5f). This enhancement is mainly attributed to the Mie-scattering interactions of infrared wavelengths within the hierarchical porous network and the bonding vibrations of mycelium and BC^55,56^. As shown in Fig. 5g, the MBC glass suppresses the temperature rise of a simulated heating display by ~3 °C relative to bare glass under identical solar illumination, consistent with infrared thermal images (Supplementary Fig. 21). In addition, the MBC-coated glass demonstrates good stability, showing no noticeable degradation after 30 days of outdoor exposure while still achieving a temperature reduction of ~2.5 °C on the heater surface (Supplementary Fig. 22).

## Discussion

In summary, we demonstrate a co-culture strategy in which fungi and bacteria are grown together to yield hydrogels and films with tunable structures and properties. Mycelium microfibers and BC nanofibers co-assemble within a designed liquid medium, enabling their simultaneous growth into an interpenetrating double network via biological crosslinking, which results in a strong, self-supporting hydrogel. The hydrogel exhibits intrinsic self-healing, enabled by continued hyphal growth and reformation of interfacial hydrogen bonds, and can be transformed upon drying into robust films. These MBC films simultaneously achieve high tensile strength (195.62 ± 9.06 MPa) and toughness (11.51 ± 0.94 MJ m^–3^), overcoming the conventional strength–toughness trade-off through efficient stress transfer and energy dissipation within the hierarchical architecture. Moreover, by regulating culture duration, the film thickness, mycelial content and interlamellar pore structure can be tuned, enabling programmable optical scattering: the haze can be modulated from 16.0% to 78.5% while maintaining ~80% transmittance. The MBC films with tailored properties are suitable for flexible and transparent coatings with effective radiative cooling and outdoor display performance, showing great potential in sustainable energy-efficient coatings and flexible optoelectronic applications. Overall, these results establish microbial regulation as a general principle for directly tailoring material structures and functions through growth control.

## Methods

### Materials

The white rot fungi *Phanerochaete chrysosporium* (PC) was purchased from Outgrow on Etsy. *Pleurotus ostreatus*, *Pholiota adiposa* and *Pleurotus columbinus* were purchased from mymushshop on Etsy. *Ganoderma sessile* was purchased from Primefungi on Etsy. *Komagataeibacter xylinus* (*K. xylinus*, ATCC 23767) for bacterial cellulose (BC) production was purchased from ATCC. Mannitol was purchased from ChemCenter. Malt extract and yeast extract were purchased from Sigma-Aldrich. Sodium hydroxide (NaOH), peptone, disodium phosphate anhydrous and citric acid monohydrate were purchased from ThermoFisher Scientific.

### Fabrication of mycelium (MY), BC, MBC hydrogels, films and coatings

For the pre-culture of fungi, 3 mL of the purchased liquid culture was inoculated into a sterilized liquid medium containing 8 g L^–1^ glucose and 1.5 g L^–1^ peptone. The fungal pre-culture was maintained at room temperature (23–25 °C) for 2 weeks, during which the culture was shaken by hand once per day to maintain a more uniform distribution of the mycelium.

For the pre-culture of BC, 3 mL of the purchased bacteria strain (*K. xylinus*) was inoculated into a sterilized liquid medium containing 5 g L^–1^ yeast extract, 3 g L^−^^1^ peptone, and 25 g L^−^^1^ mannitol. The bacterial pre-culture was maintained under static conditions at room temperature for 3 weeks, during which a BC pellicle formed at the air-liquid interface. For inoculation, the liquid culture collected from beneath the BC pellicle was used.

Both fungal and bacterial pre-cultures were prepared in sterilized 500 mL bottles, with the culture volume maintained at approximately half of the bottle volume. After cultivation, the fungal and bacterial pre-cultures were stored at 3 °C to maintain relatively stable inoculum concentrations and were directly used for subsequent co-culture experiments. No BC pellicles or mycelial mats were directly transferred between experiments. The OD600 value of the liquid culture collected from beneath the BC pellicle was 0.018 ± 0.001, while that of the fungal liquid culture was 0.023 ± 0.006.

Besides, the MY hydrogel was prepared by inoculating 3 mL of fungal pre-culture into 10 mL of LM medium containing 10 g L^–1^ malt extract, 5 g L^–1^ yeast extract. The BC hydrogel was prepared by inoculating 3 mL of bacterial pre-culture into 10 mL of HS medium containing 20 g L^–1^ glucose, 5 g L^–1^ yeast extract, 5 g L^–1^ peptone, 2.7 g L^–1^ disodium phosphate anhydrous and 1.5 g L^–1^ citric acid monohydrate. The co-culture medium (LM-HS) is the combination of LM and HS medium with a volume ratio of 7:3. All the liquid medium was sterilized by autoclaving at 121 °C for 15 min. For the co-culture of mycelium and BC, 3 mL of fungal pre-culture and 3 mL of bacterial pre-culture were added into 10 mL of LM-HS medium in a petri dish (diameter: 5 cm; height: 1 cm), followed by static incubation for 4–10 days to allow hydrogel formation. All procedures were conducted under sterile conditions. All cultures were incubated under room temperature (23–25 °C) for desired culture duration (4 days, 6 days and 8 days). To prepare films, the hydrogels were immersed in 0.01 M NaOH solution at 80 °C for 1 h to remove residual cells and nutrients. They were then washed alternately with ethanol and deionized water until the pH reached neutral. Finally, the hydrogels were air-dried on a polytetrafluoroethylene (PTFE) substrate at room temperature with a relative humidity of 30% for 24 h to obtain films. MBC glasses and MBC-PI films were prepared by simply air-drying MBC hydrogels on glasses and PI films as coatings.

### Characterizations

The cross-sectional scanning electron microscopy (SEM) images of the films were obtained using an Apreo 2S SEM (Thermo Fisher Scientific, USA). The microstructure of the hydrogels and the morphology of hyphae were characterized by Apreo 2S SEM equipped with a Cryo-prep system (Quorum, USA). Energy-dispersive X-ray spectroscopy (EDS) was performed using a Helios G4 UX dual-beam system to evaluate the elemental composition of the films. All EDS spectra were collected at an accelerating voltage of 10 kV and a beam current of 0.1 nA. The relative nitrogen content was calculated by dividing the nitrogen atomic percentage by the sum of the carbon, nitrogen, and oxygen atomic percentages obtained from EDS analysis software Aztec 6.3. All films used for EDS analysis were prepared from NaOH-treated hydrogels followed by thorough washing. The sample was first frozen in liquid nitrogen and subsequently transferred to the fracture chamber under a nitrogen purge. Inside the chamber, the specimen was fractured to expose its cross-section. To enhance imaging quality, the fractured surface was sputter-coated with a 4 nm platinum layer. The OD600 values of four independent fungal pre-cultures and four independent bacterial pre-cultures were measured using an ultraviolet-visible (UV–vis) spectrophotometer (Shimadzu, UV-2600, Japan). The surface element composition of pure mycelium film was characterized using X-ray photoelectron spectroscopy (XPS, Kratos, UK) and the data were analyzed using Avantage 6.60. The orientation of fibers was characterized using small-angle X-ray scattering (SAXS, Anton Paar SAXS point 2.0, Austria). The SAXS data were analyzed using SAXS analysis 4.1. The chemical structure of the films was analyzed by attenuated total reflectance Fourier transform infrared spectroscopy (ATR-FTIR, Thermo Fisher Scientific, Nicolet 6700, USA) in the range of 4000–400 cm^–1^. The thermostability of the films was tested by thermogravimetry (TA Instruments, SDT Q600, USA) at temperatures ranging from 25 °C to 600 °C at a heating rate of 20 °C min^–1^ in nitrogen. The pore fraction data was obtained from the SEM images using ImageJ v1.54 g. Root mean surface roughness of the MBC film was obtained using an atomic force microscopy (AFM, Oxford Instruments, Asylum Cypher, UK). The AFM images were analyzed using Gwyddion 2.70. The thicknesses of the MBC films were estimated from cross-sectional SEM images by measuring six different positions for each sample, and the average value was calculated. The IR light transmittance and reflectance in the wavelengths of 8–13 μm region were measured using a Fourier transform infrared spectrometer (Thermo Scientific, Nicolet iS50, USA).

### Biodegradability test

The biodegradability of the MBC film (2 cm × 2 cm) was evaluated by burying the film at a depth of approximately 2 cm in soil contained within an aluminum box for 4 weeks. The setup was placed in the laboratory at room temperature (23–25 °C) and a relative humidity of 30%. To maintain soil moisture, 50 mL of water was added to the soil every 4 days throughout the experiment. The soil was not replaced during the 4-week burial period. The MBC film used in this experiment was obtained by air-drying a NaOH-treated MBC hydrogel. The area of the film was recorded before and after burial for comparison.

### Mechanical properties test

The tensile properties of the films were measured using a tensile tester (ADMET, USA). The films were cut into strips with a width of ~4 mm and conditioned at 30% relative humidity for 24 h at room temperature prior to testing. The MBC hydrogels were cut into specimens with widths of 10–15 mm and a thickness of ~1 mm. All experiments were conducted at a loading rate of 1 mm/min. Four independently grown samples were tested for each film type. The cyclic tensile test of MBC hydrogel was performed by Univert testing system (CellScale, Canada), the hydrogel sample was 5 mm in width and ~1 mm in thickness.

### Self-healing test

After 8 days of culture, the MBC hydrogel sample was removed from the liquid medium and directly cut into two pieces using a sterilized blade. No washing step was performed before rejoining to preserve the living cells and nutrients within the hydrogel. The two cut pieces were immediately brought back into contact and placed in a sterilized petri dish. After 12 h of healing, the two cut pieces had reconnected into an integrated hydrogel. After another 24 h of healing, the sample was freeze-dried, and the healed fracture region was observed by SEM.

### Optical properties test

The optical transmittance and haze of the films were measured using a UV–vis spectrophotometer from 400 nm to 800 nm (Shimadazu, UV-2600, Japan). The transmittance and haze value at 550 nm of the films were used for comparison. Three independently grown samples were tested for each film type.

### Electromagnetic simulation for the light scattering of MBC films

A 550 nm light source was simulated to pass through a two-dimensional material domain of 10 µm × 10 µm, consisting of a bacterial cellulose matrix (refractive index, *n* = 1.56) with embedded mycelium hyphae (diameter ~2 µm, *n* = 1.41). The full electromagnetic field was solved using COMSOL 6.0. The transmitted light was collected by a hemispherical receiver with a radius of 20 µm to illustrate the light scattering effect.

### Radiative cooling tests

Field tests were conducted from 13:00 to 14:00 on November 3, 2025, and 12:20 to 13:20 on March 31, 2026, on the roof of Herrick Labs under clear sky conditions in West Lafayette, Indiana, USA. Heaters (40 mm × 40 mm) covered with bare glass and MBC glass, respectively, were used as simulated display modules. The samples were placed on a polystyrene foam substrate facing upward toward the sky during the measurement. The polystyrene foam was covered with aluminum to reduce solar absorption. The setup was sealed with a polyethylene film to reduce heat convection. A heating power density of 300 W m^–2^ was maintained using a power supply (HM305, HANMATEK) connected to the heater. K-type thermocouples (Omega 5TC-TT-K-30-72) were fixed using thermal tape between the glass and heater to measure the surface temperature, and temperature readings were recorded using a data logger (MCC USB-TC) and LabVIEW 2019 software. Another thermocouple was placed in the shade surrounded by white polystyrene foam to measure the ambient temperature. Infrared images were obtained using an infrared thermal camera (E8-XT, FLIR).

### Reporting summary

Further information on research design is available in the Nature Portfolio Reporting Summary linked to this article.

## Supplementary information

Supplementary Information

Reporting Summary

Transparent Peer Review file

## Source data

Source data

## Data Availability

The data that support the findings of this study are available from the corresponding author upon request. Source data are provided with this paper.
