# Supplementary material for: Living co-culture fabrication for biologically crosslinked mycelium–cellulose hydrogels and films
Source: Nat Commun. 2026 Aug 24;17:10134. doi: 10.1038/s41467-026-76430-6 (PMC13601560; doi:10.1038/s41467-026-76430-6)
Supplement: Supplementary file 1 [file 41467_2026_76430_MOESM1_ESM.pdf]

Supplementary Information

Living Co-culture Fabrication for Biologically Crosslinked Mycelium–Cellulose Hydrogels and Films

Authors:

Bingyu Xia[1], Yanpei Tian[1], Qilong Cheng[1], Huilong Liu[1], Xiaojie Liu[1], Zhenyuan Niu[1], Xiwei Shan[1], Pengfei Deng[1], Wenhui Xu[1], Shubhra Bansal[1,2], Tian Li[1]*

Affiliations:

1 School of Mechanical Engineering, Purdue University, West Lafayette, IN 47907, USA.

2 School of Materials Engineering, Purdue University, West Lafayette, IN 47907, USA.

*Email: tianli@purdue.edu

Supplementary Notes

The light scattering mechanism of the bacerial cellulose (BC), mycelium (MY) and mycelium/bacterial cellulose (MBC) films are based on the following equations:

Supplementary Note 1

For the light reflection at interface between different components:

$$\Gamma = \left(\frac{n_1 - n_2}{n_1 + n_2}\right)^2 \tag{1}$$

where Γ is the reflection coefficient, n_1 and n_2 are the redractive index (RI) of different substances at the interface.

Supplementary Note 2

For the Rayleigh scattering within pure BC films:

$$Q_{\mathrm{sca}} = \frac{8}{3}\left(\frac{2\pi}{\lambda}\right)^4 \left(\frac{n^2 - 1}{n^2+2}\right)^2 r^4 \tag{2}$$

where Q_{sca} is the Rayleigh scattering efficiency, λ is the wavelength of the light, r is the radius of the fiber, n is the refractive index of the bacterial cellulose.

Supplementary Note 3

For the Mie scattering within pure MY and MBC films:

$$Q_{\mathrm{sca}} = \frac{2}{x^2}\sum_{n=1}^{\infty}(2n+1)(|a_n|^2+|b_n|^2) \tag{3}$$

where Q_{sca} is the Mie scattering efficiency, x is the size parameter ($x = 2\pi r/\lambda$, λ is the wavelength of the light, r is the radius of the fiber).

Supplementary Note 4

a_n and b_n are Mie Scattering Coefficients and are functions of x and m:

$$a_n = \frac{m\psi_n(mx)\psi_n'(x) - \psi_n(x)\psi_n'(mx)}{m\psi_n(mx)\xi_n'(x) - \xi_n(x)\psi_n'(mx)}$$

$$b_n = \frac{\psi_n(mx)\psi_n'(x) - m\psi_n(x)\psi_n'(mx)}{\psi_n(mx)\xi_n'(x) - m\xi_n(x)\psi_n'(mx)} \quad (4)$$

where m = n_f/n_m being the refractive-index ratio between the fiber and surrounding medium.

Supplementary Note 5

$$\mathrm{TIS} = 1 - \exp\left[-\left(\frac{4\pi\sigma\cos\theta_{\mathrm{i}}}{\lambda}\right)^2\right] \quad (5)$$

where TIS is the total integrated scattering, θ_i is the incident angle, λ is the laser wavelength, σ is the root mean square (RMS) roughness.

Supplementary Figures

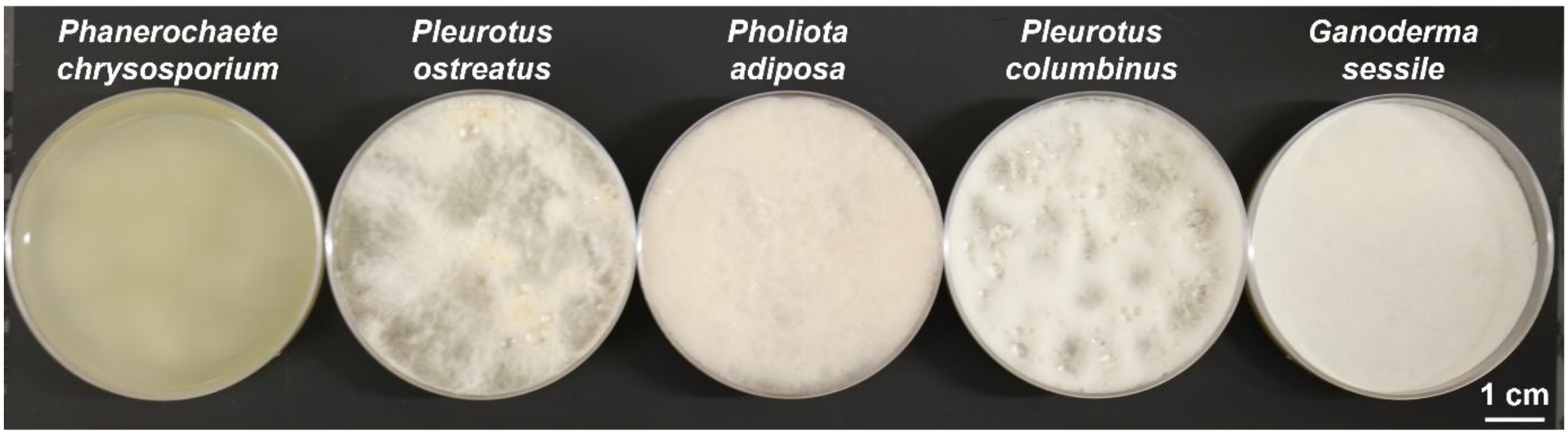

Supplementary Fig. 1. Fungal species screening. Photographs of 5 species grown in LM medium (liquid medium for mycelium hydrogel) under room temperature for 8 days.

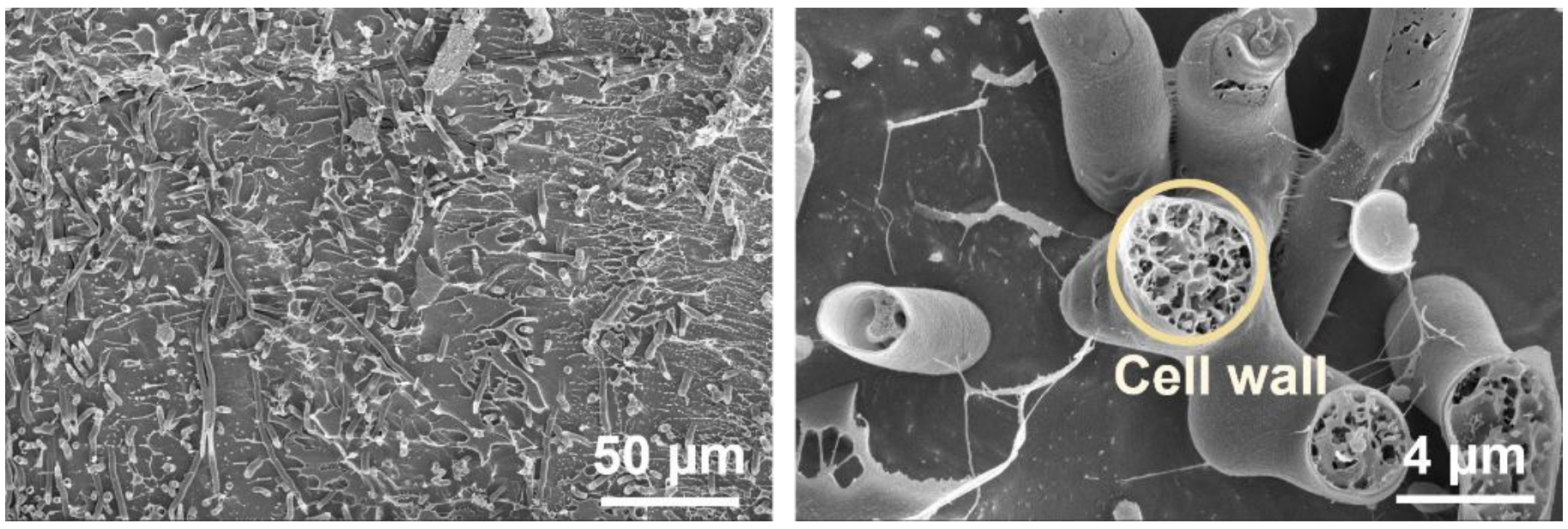

Supplementary Fig. 2. Morphology of mycelium hyphae. Cryo-scanning electron microscopy (Cryo-SEM) images of mycelium hyphae at different magnifications.

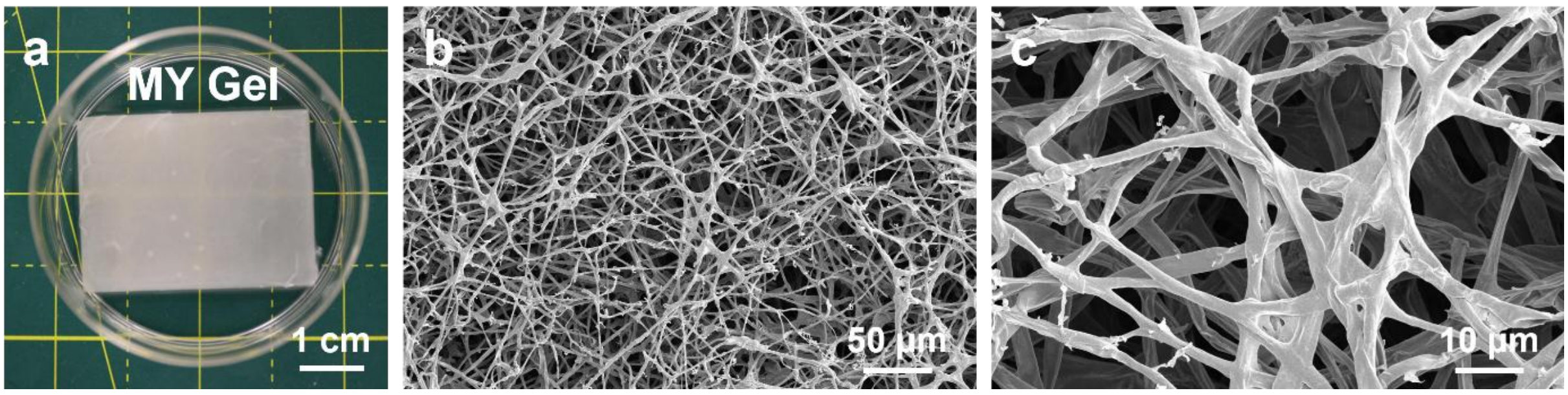

Supplementary Fig. 3. Morphology of MY hydrogel. a Photograph of MY hydrogel. **b, c** SEM images of MY hydrogel.

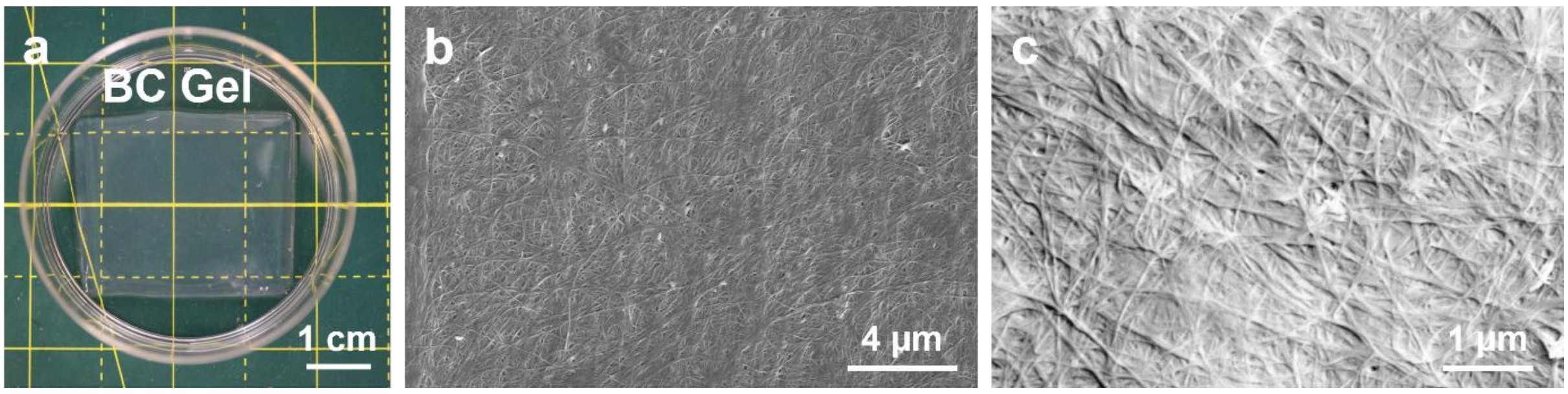

Supplementary Fig. 4. Morphology of BC hydrogel. a Photograph of BC hydrogel. **b, c** SEM images of mycelium hydrogel.

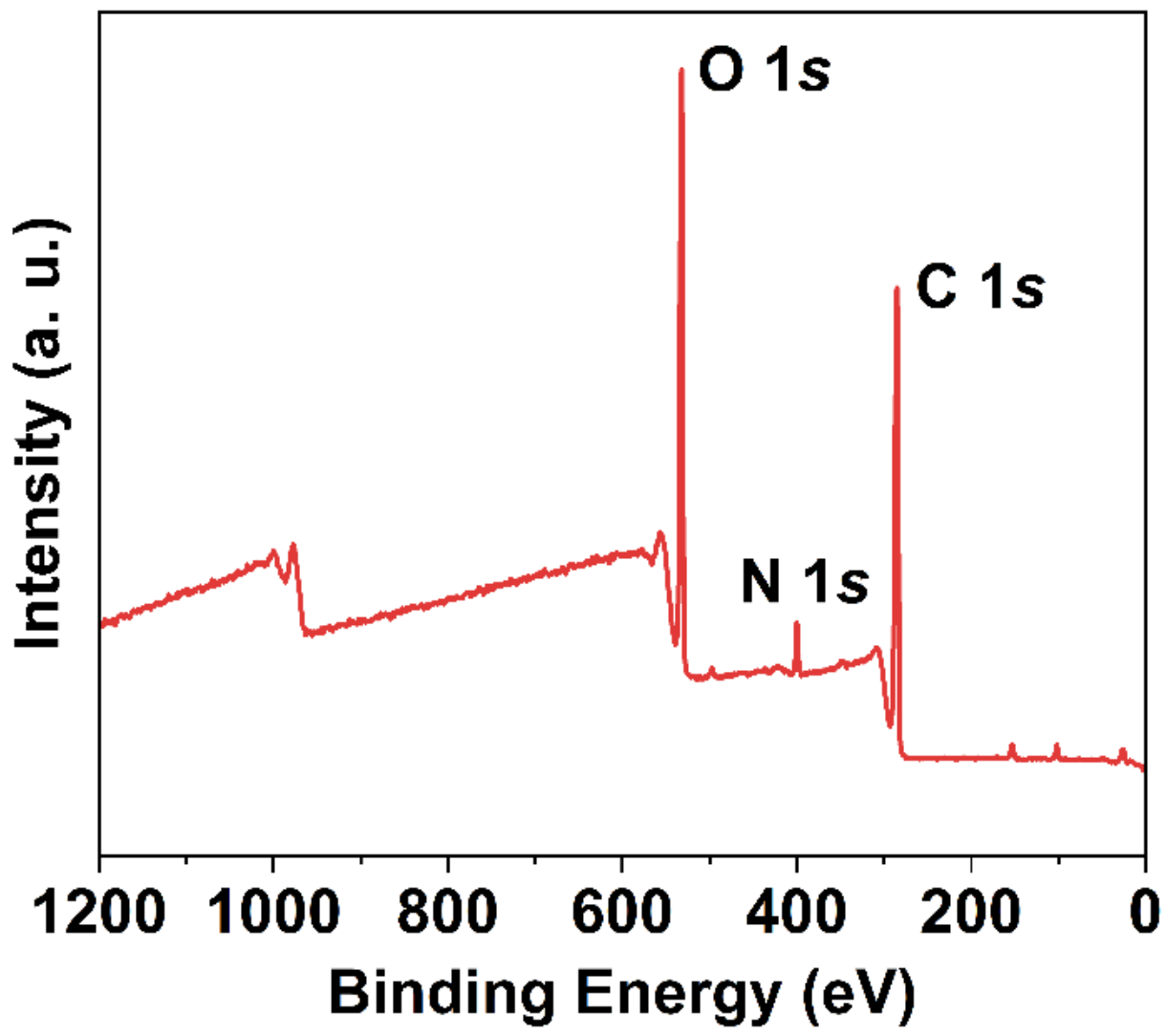

Supplementary Fig. 5. Surface element composition of mycelial hyphae. X-ray photoelectron spectroscopy (XPS) spectrum of pure mycelium film.

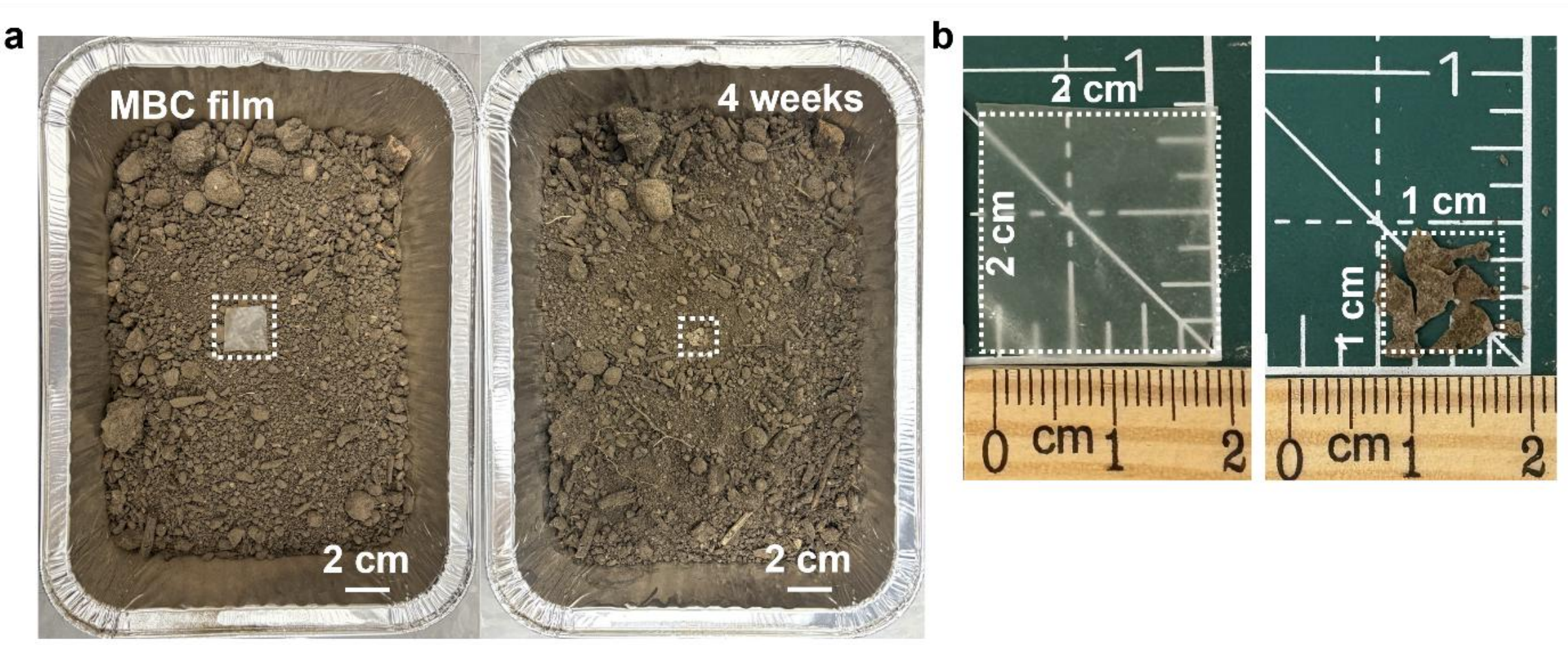

Supplementary Fig. 6. Biodegradability of the MBC film. a Photographs of the MBC film before and after 4 weeks of soil burial. **b** Photographs showing the area of the MBC film before and after 4 weeks of soil burial.

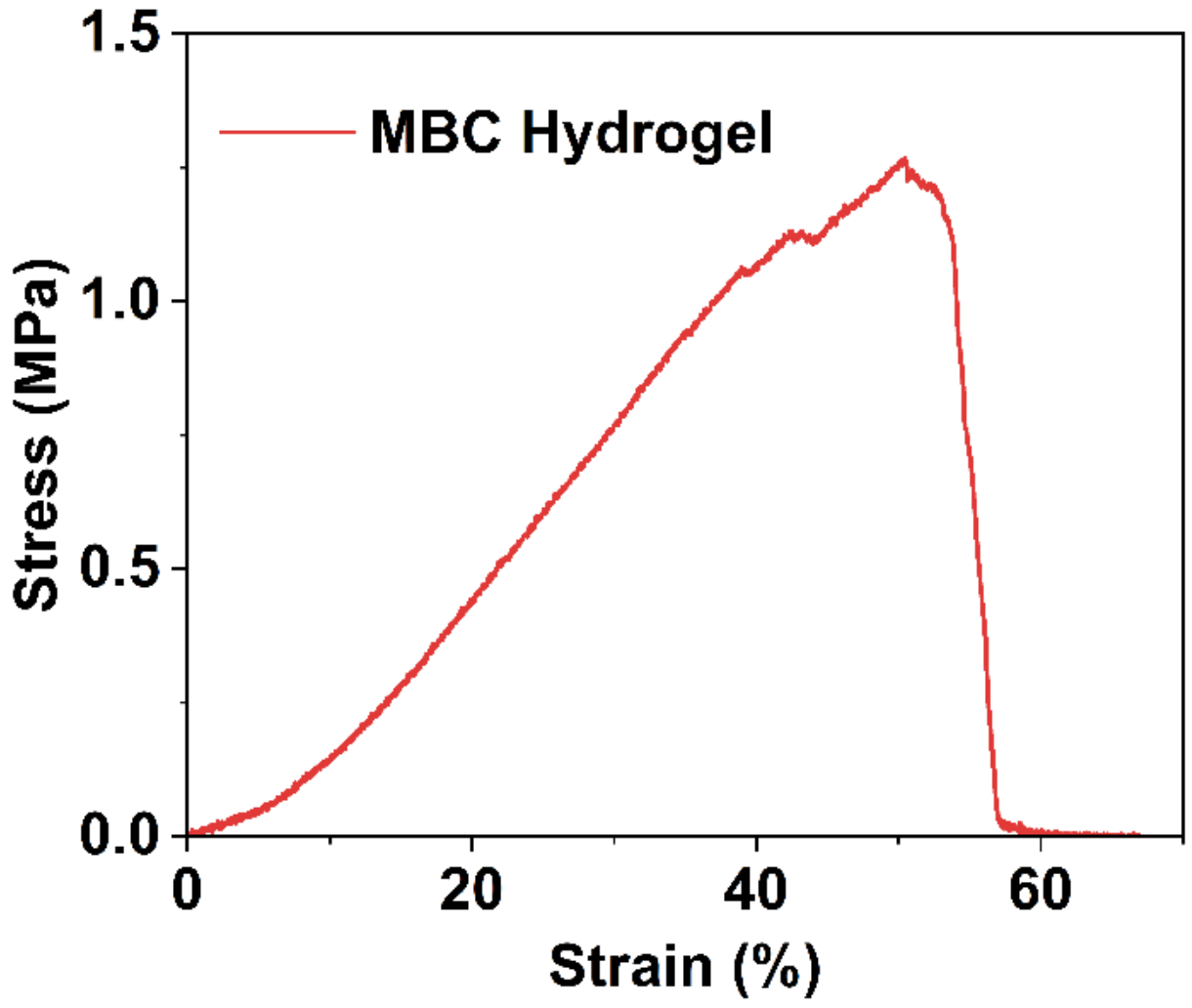

Supplementary Fig. 7. Tensile property of MBC hydrogel. Strain-stress curve of MBC hydrogel.

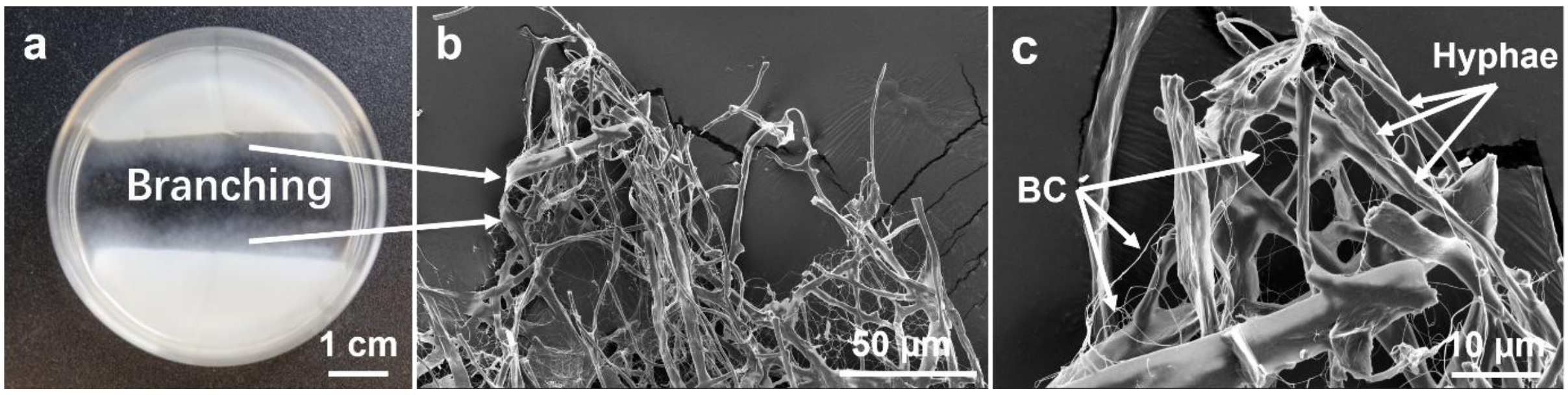

Supplementary Fig. 8. Self-healing and re-growth of MBC hydrogels. a Photographs showing the self-healing and re-growth of MBC hydrogel. **b, c** SEM images of the newly grown thin layer composed of hierarchical BC nanofibers and hyphae.

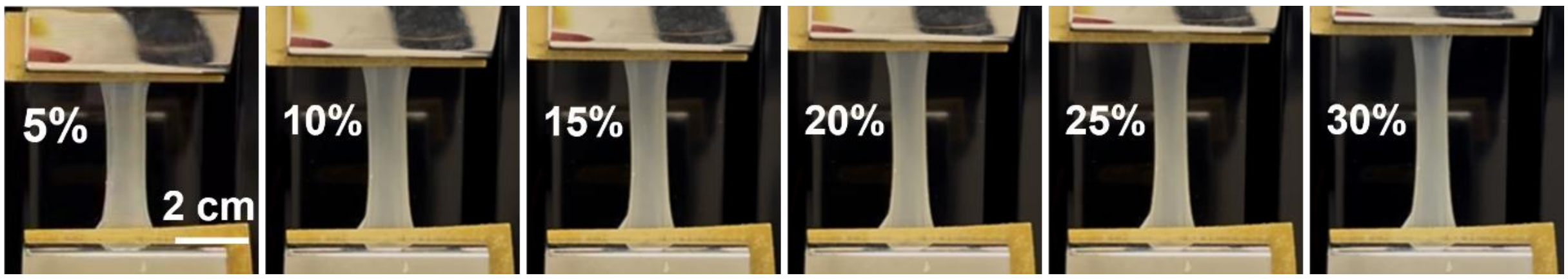

Supplementary Fig. 9. Cyclic tensile behavior of the MBC hydrogel. Photographs of the MBC hydrogel under different strains (5%–30%).

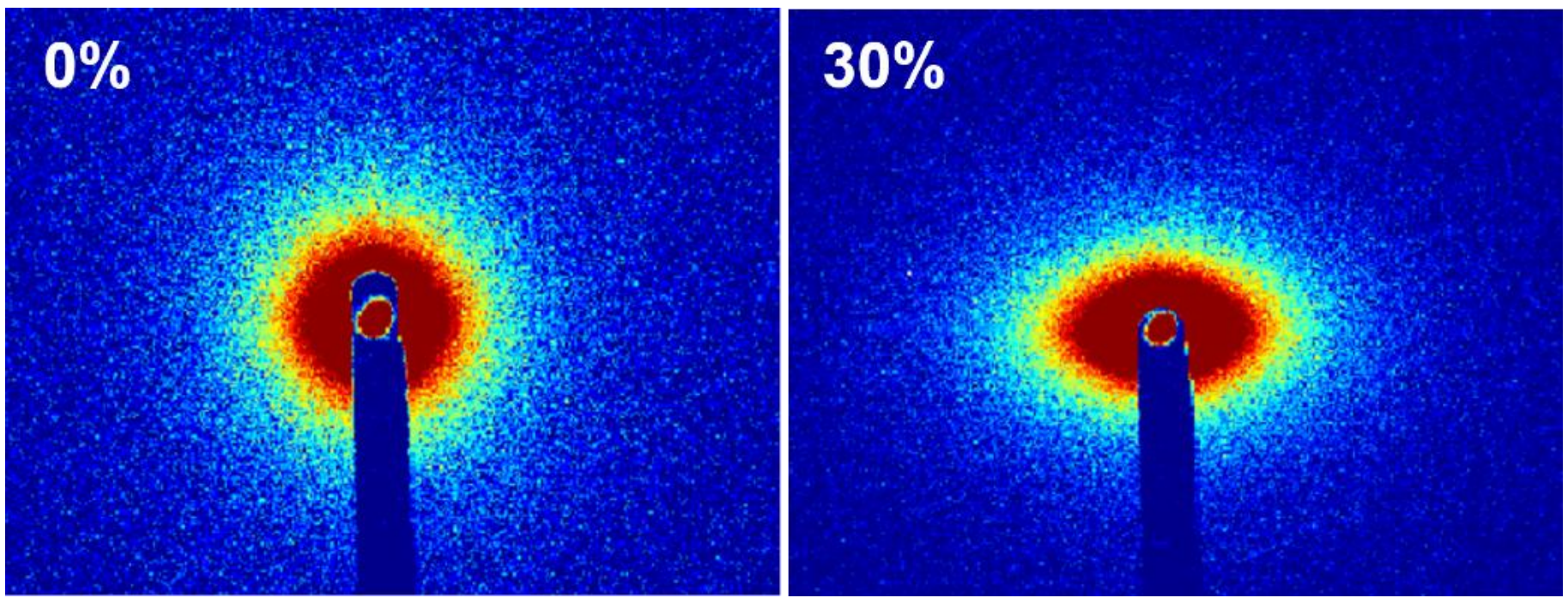

Supplementary Fig. 10. Fiber reorientation within the MBC hydrogel at 30% strain. 2D Small-angle X-ray scattering (SAXS) patterns of the MBC hydrogel under different tensile strains (0% and 30%).

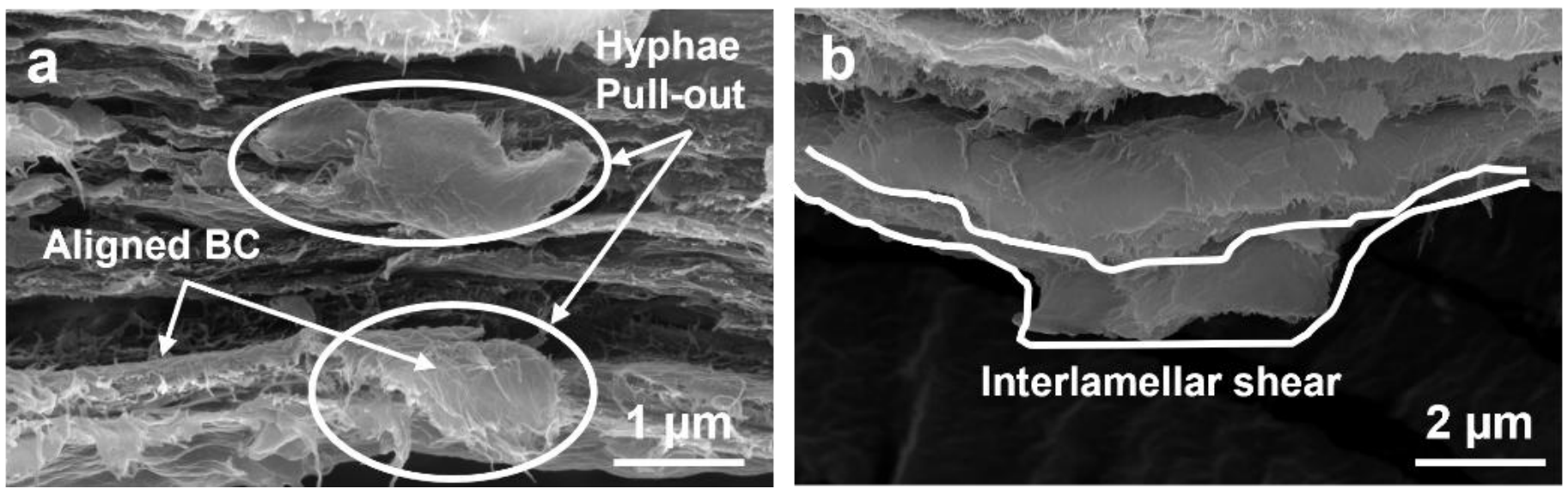

Supplementary Fig. 11. SEM images of the fracture surface of MBC films. a Deformation of MBC films. **b** Interlamellar shear of MBC films.

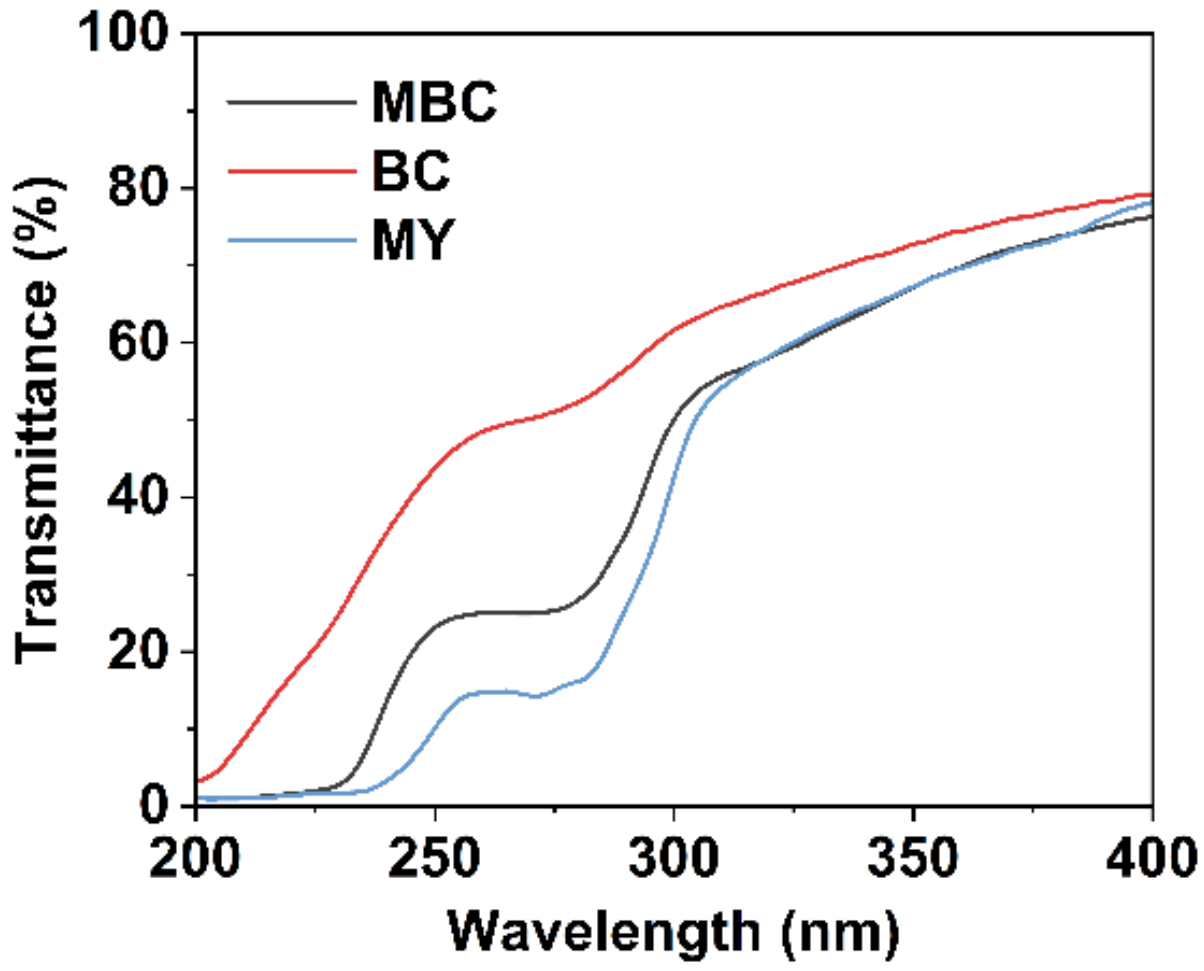

Supplementary Fig. 12. UV blocking performance of the films. Transmittance spectra of MY, BC and MBC films in the UV region (200–400 nm).

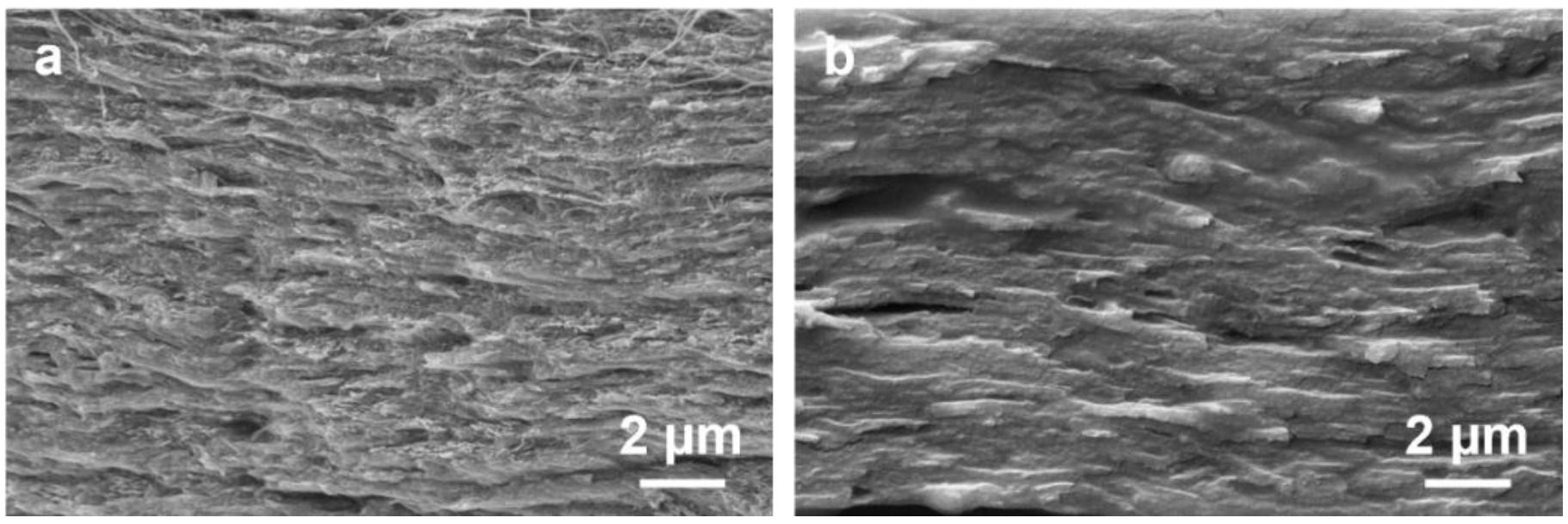

Supplementary Fig. 13. SEM images after liquid-nitrogen fracturing. Cross-sectional SEM images of **a** Pure BC and **b** MY films.

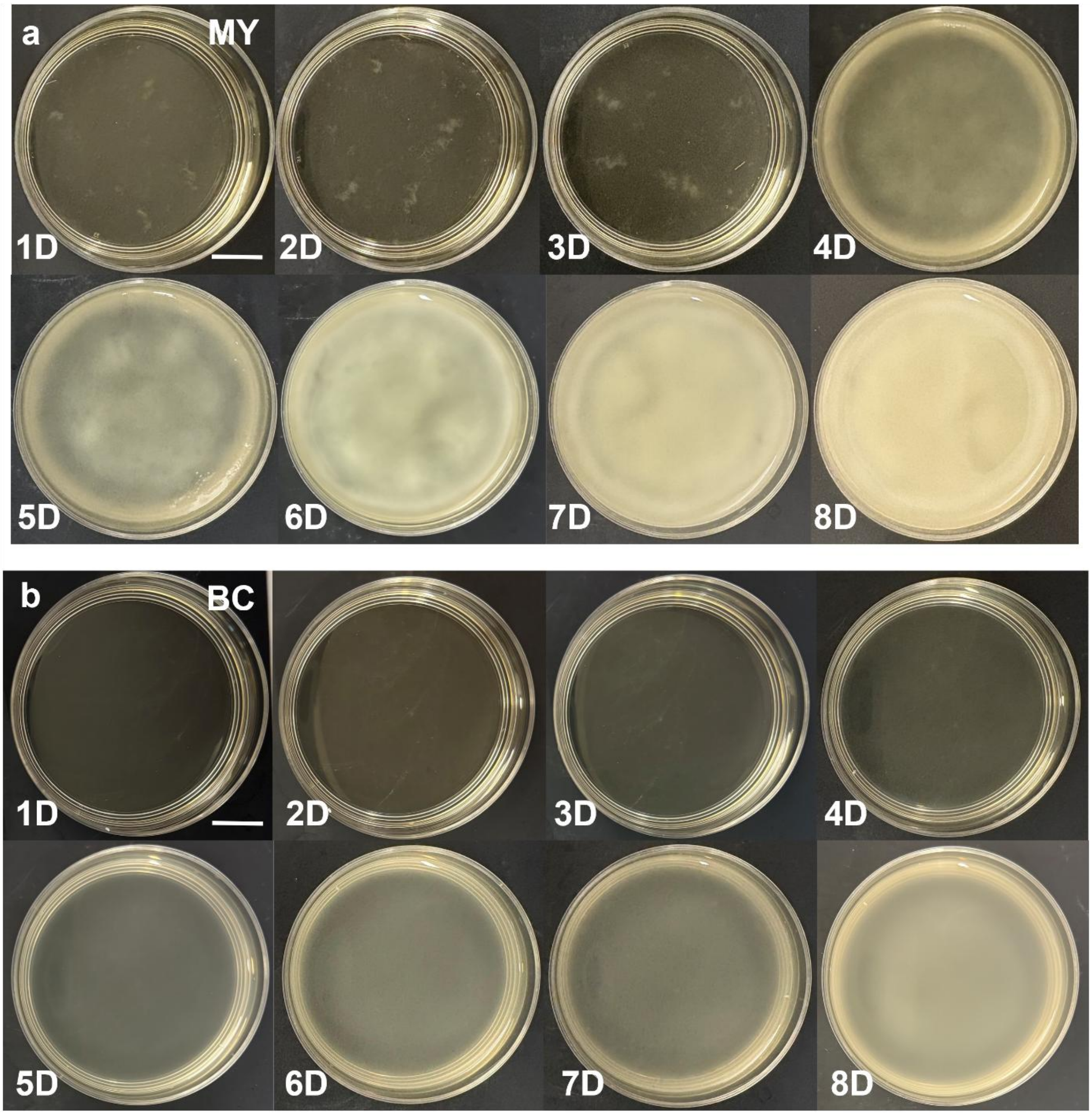

Supplementary Fig. 14. Growth process of microorganisms. a Growth process of the MY hydrogel over 8 days. **b** Growth process of the BC hydrogel over 8 days. D denotes days. Scale bars: 1cm.

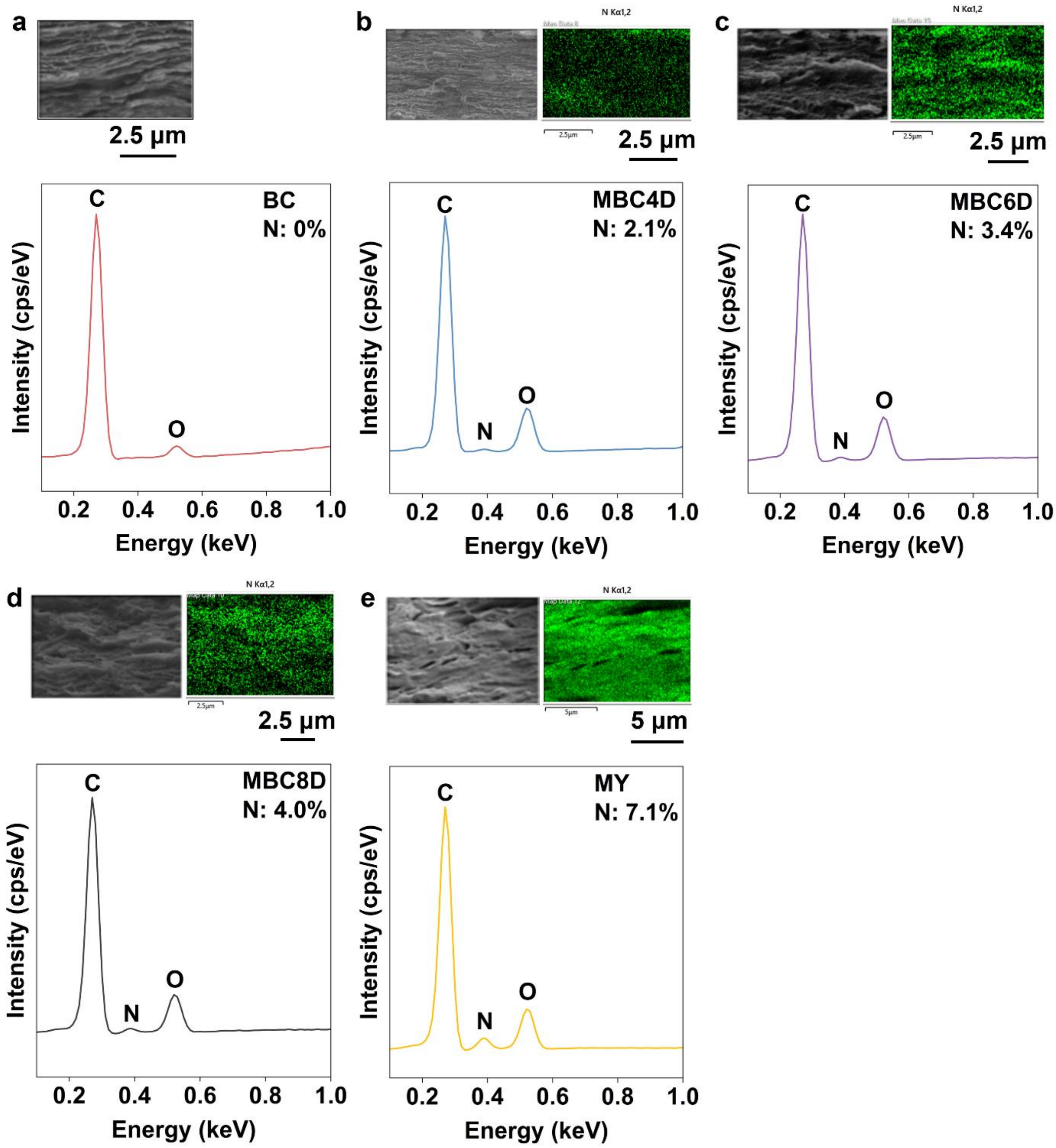

Supplementary Fig. 15. Energy-dispersive X-ray spectroscopy (EDS) analysis of BC, MY and MBC films with different culture durations. a–e SEM images, EDS nitrogen elemental maps, and EDS spectra of **a** BC, **b** MBC4D, **c** MBC6D, **d** MBC8D and **e** MY films.

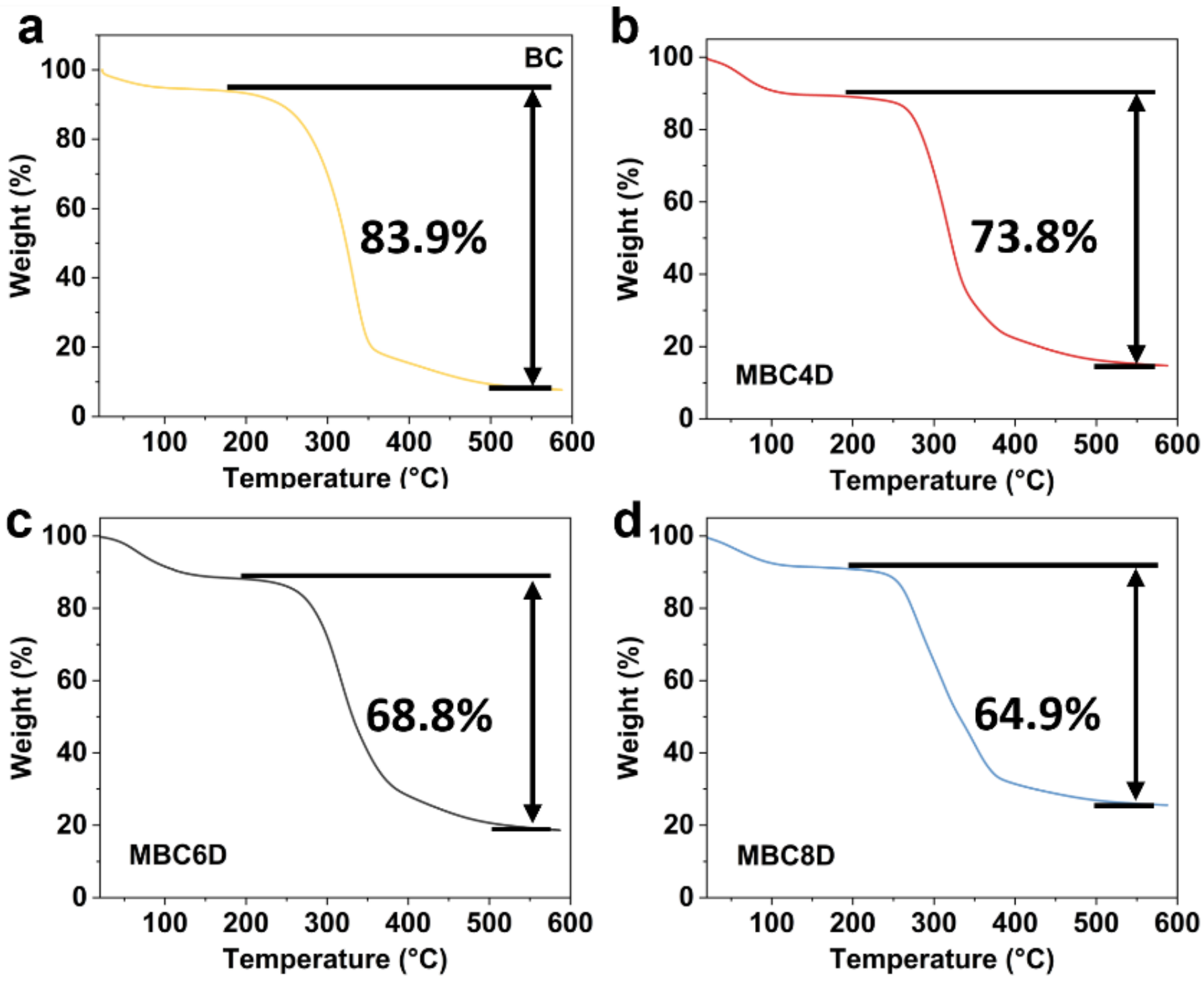

Supplementary Fig. 16. Thermal degradation analysis of pure BC and MBC films. a–d Thermogravimetric analysis (TGA) curves of pure BC film and MBC films with diffrent culture durations (MBC4D, 4 days; MBC6D, 6 days; MBC8D, 8 days).

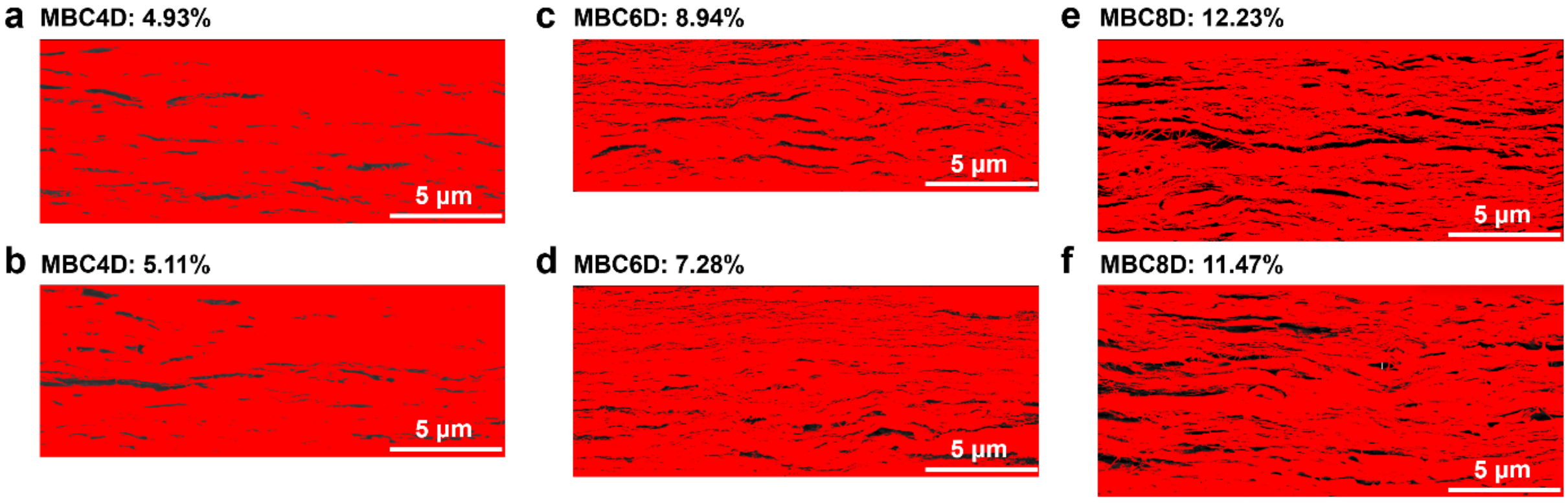

Supplementary Fig. 17. Porous structure of MBC films. Cross-sectional porosity analysis of MBC films with different culture durations using ImageJ.

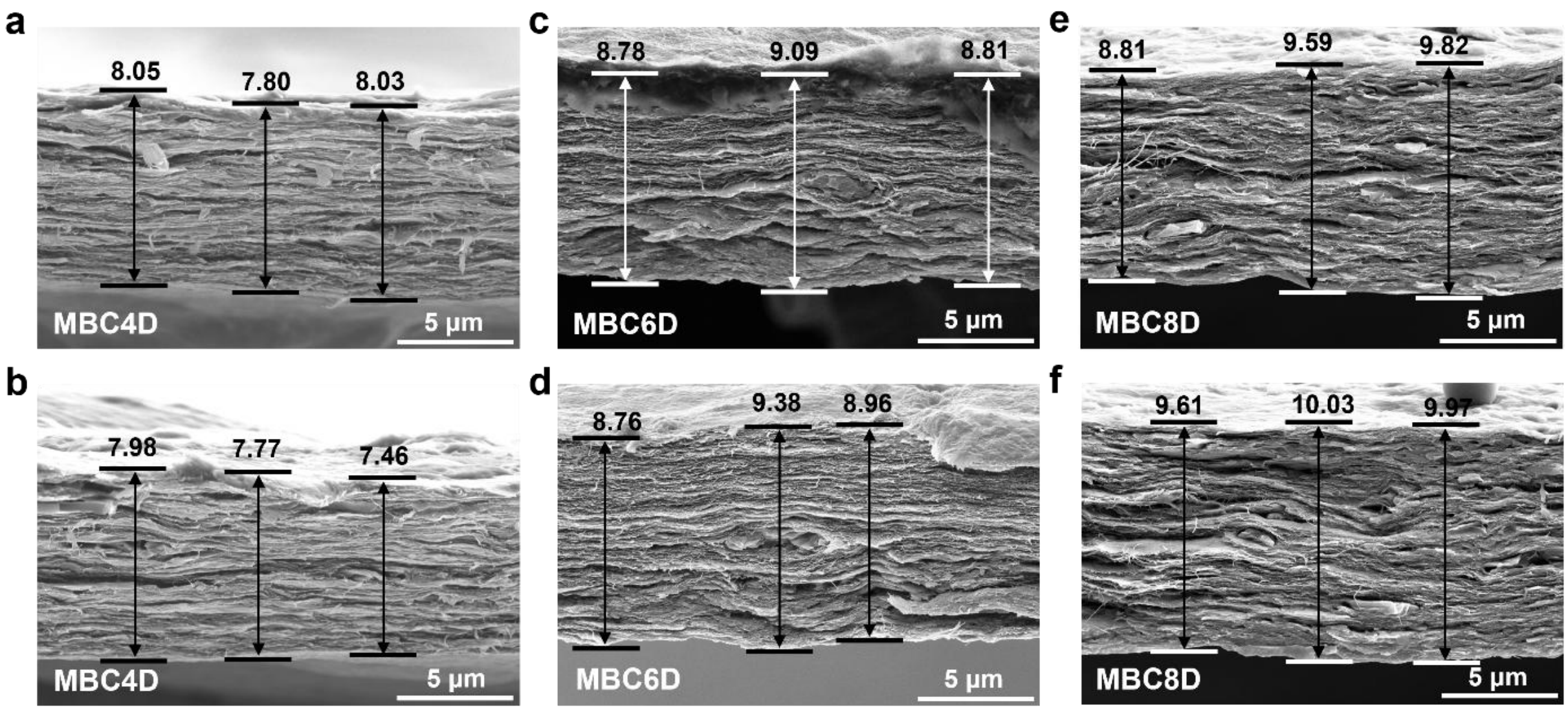

Supplementary Fig. 18. Thicknesses of MBC films. a–f Cross-sectional SEM images of MBC films with different culture durations (4 days, 6 days and 8 days).

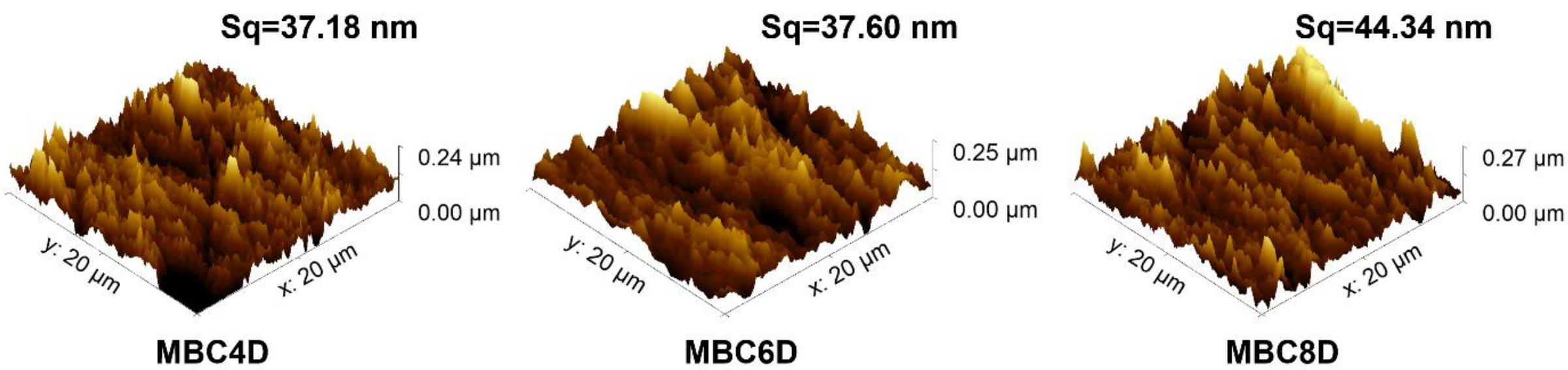

Supplementary Fig. 19. Surface structures of MBC films. Root mean square (RMS) roughness of MBC films with different culture duarations. Sq is the RMS roughness.

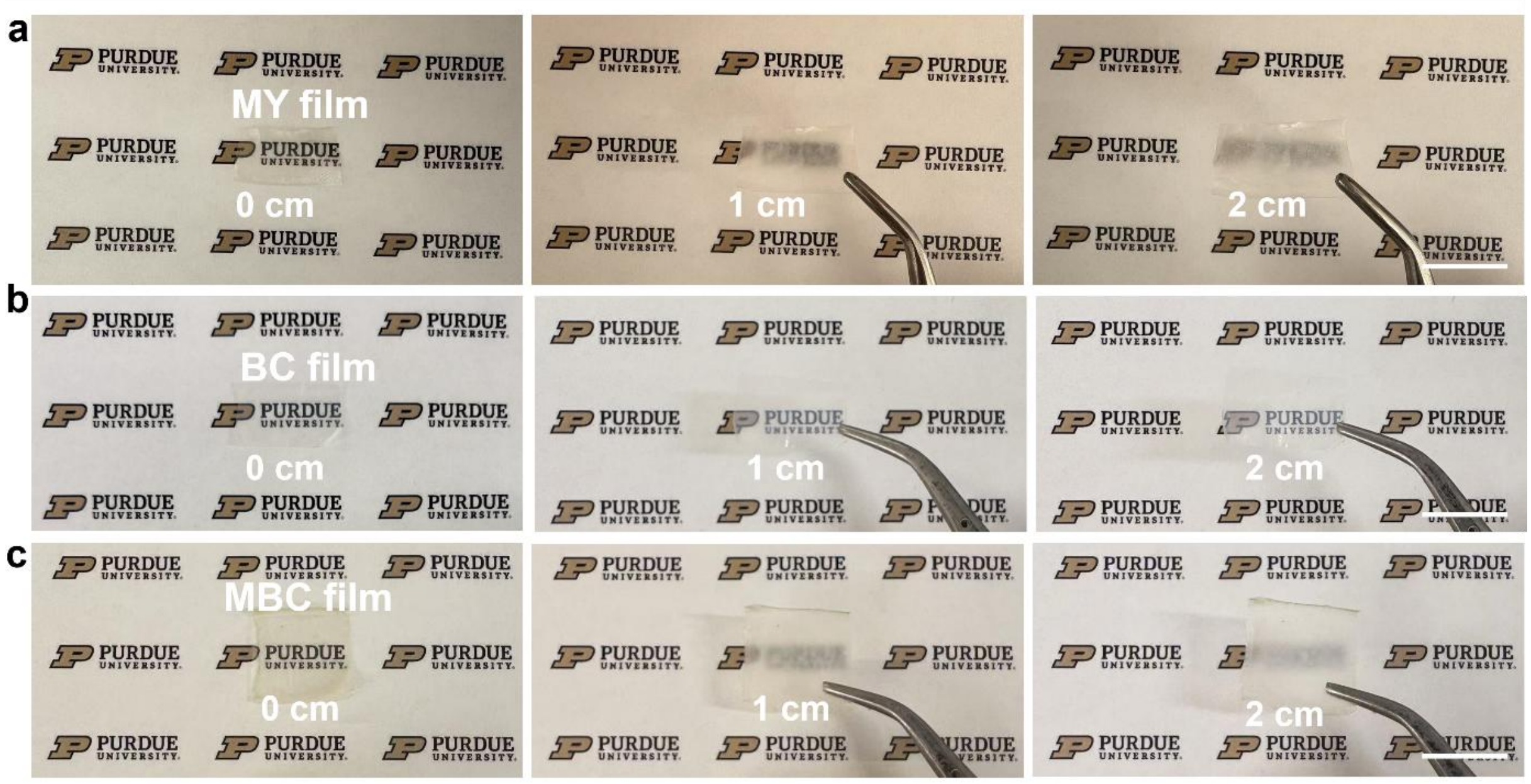

Supplementary Fig. 20. Pattern visibility tests. Photographs showing the view of pattern under **a** MY film, **b** BC film and **c** MBC film at different viewing distances (0 cm, 1 cm, 2 cm). Scale bars: 2 cm.

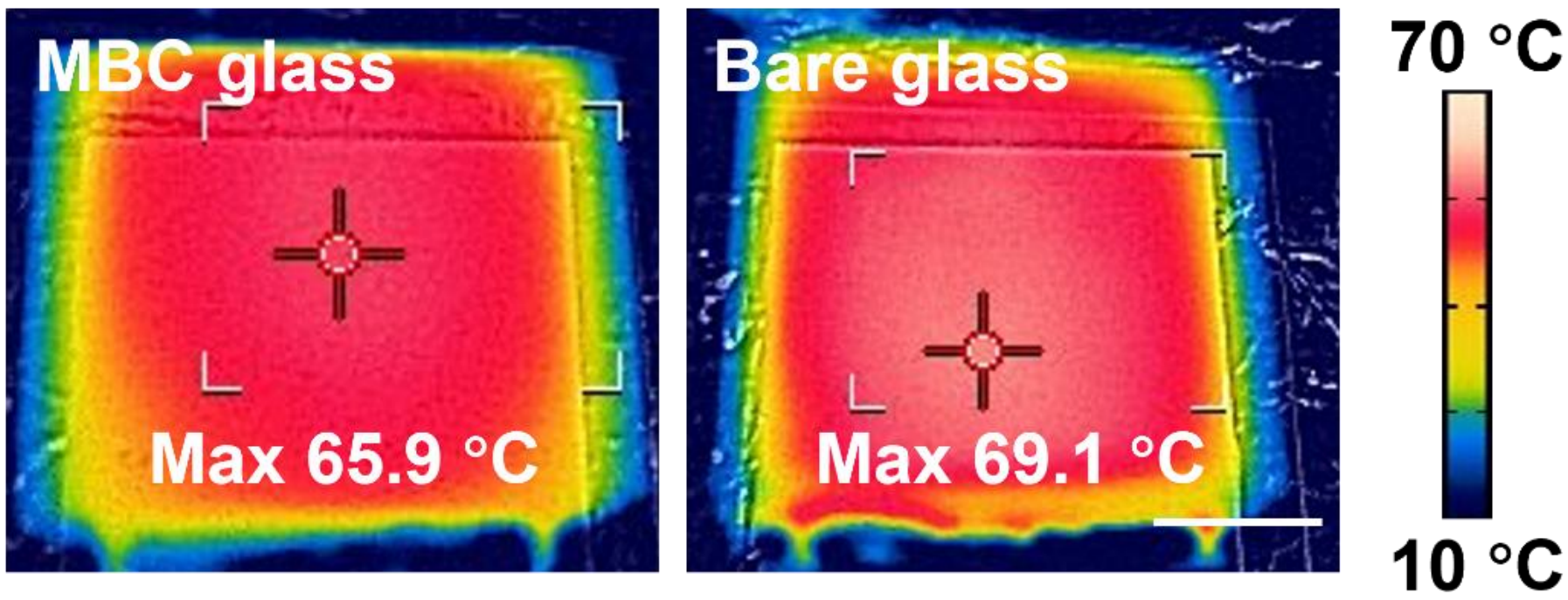

Supplementary Fig. 21. Surface temperature of simulated heater covered by MBC glass and bare glass. Infrared thermal images of MBC glass and bare glass. Scale bar: 2 cm.

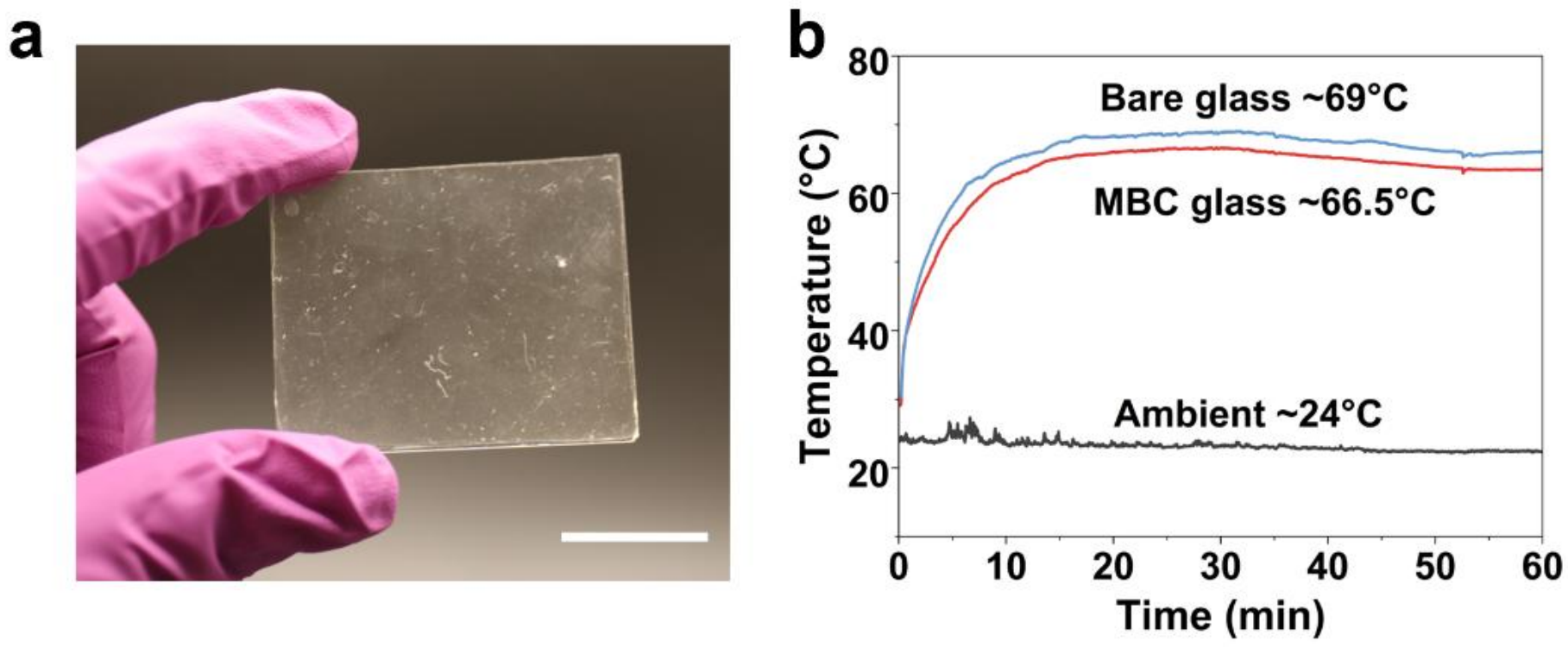

Supplementary Fig. 22. Durability of MBC glass after 30-day outdoor exposure. a Photograph of MBC glass after 30-day outdoor exposure. **b** Temperature of heater covered by bare glass and MBC glass under solar illumination. Scale bar: 2 cm.

Supplementary Information Tables

Supplementary Table 1. Comparison of mechanical properties of MBC film and other biomass-based films (CNF, cellulose nanofiber; TOCNF, TEMPO-oxidizedCNF; CNC, cellulse nanocrystal; SF-CNF, Self-fibrillating CNF; H-CNF, HoloCNF; CNT, Carbon nanotube; C-CNF, Carboxylated CNF).

Materials	Tensile strength (MPa)	Elongation (%)	Toughness (MJ m^{-3})	Ref.
This work	195.62	10.72	11.51	
TOCNF	105	2.1	1.88	1
Aligned CNF	140	9.0	8.5	2
Aligned CNC	79.7	0.88	0.415	3
Pollen film	52.22	6.15		4
CNF/lignin	223.8	4.4	8.76	5
Bamboo film	169.03	2.6		6
Chitin film	227	2.8		7
Wood film	106.5	13.2	7.7	8
SF-CNF	184	4.6		9
H-CNF	159.39	8.35	7.36	10
CNF/CNT	97.9	4.6	2.1	11
Pure BC	194	3.4	3.8	12
Pure CNF	21.5	2.7	0.29	13
C-CNF	115.4	8.7	4.98	

Supplementary Table 2. Comparison of optical properties of MBC film and other biomass-based films (PVA, Polyvinyl alcohol; R-CNF, Regenerative CNF; PVP, Polyvinyl pyrrolidone).

Materials	Haze (%)	Transmittance (%)	Thickness (μm)	Ref.
This work	16.0 ~ 78.5	78.1 ~ 88.5	9.64	
CNF/PVA	60	90	102	14
R-CNF	41	92	60	15
TOCNF	62	85		16
Wood fiber film	62.3	88	42.5	17
Aligned CNF	70	90	50	18
CNF/PVP	63.4	89.5		19
Pollen film	65.47	91.19	100	4
CNF/Lignin	70 ~ 78	60 ~ 85	20	20
Bamboo film	81.8 ~ 83.6	66.5 ~ 89.4	500	6
Wood film	69	75	90	21
Chitin film	29.9	89.9	11	7

Supplementary References

1. Fang, Z. *et al.* Novel Nanostructured Paper with Ultrahigh Transparency and Ultrahigh Haze for Solar Cells. *Nano Lett.* **14**, 765-773, (2014).
2. Hu, W. *et al.* Transparent and Hazy All-Cellulose Composite Films with Superior Mechanical Properties. *ACS Sustainable Chem. Eng.* **6**, 6974-6980, (2018).
3. Zhang, X. *et al.* Uniformly aligned flexible magnetic films from bacterial nanocelluloses for fast actuating optical materials. *Nat. Commun.* **13**, 5804, (2022).
4. Qiu, Y. *et al.* Coassembly of hybrid microscale biomatter for robust, water-processable, and sustainable bioplastics. *Sci. Adv.* **11**, eadr1596, (2025).
5. Xu, Y. *et al.* Metal Ion Cross-Linked Cellulose/Lignin Nanocomposite Films: A Pathbreaking Approach toward High-Performance Sustainable Biomaterials. *ACS Nano* **19**, 9801-9813, (2025).
6. Wang, W. *et al.* Flexible and transparent bamboo composites Enabled by a Dual-Resin blending strategy. *Chem. Eng. J.* **509**, 161256, (2025).
7. Ma, J. *et al.* Uniform Micronano Network Affords All-Chitin Films with Water Resistance, Biodegradability, and High Strength. *ACS Sustainable Chem. Eng.* **12**, 3081-3088, (2024).
8. Jia, C. *et al.* From Wood to Textiles: Top-Down Assembly of Aligned Cellulose Nanofibers. *Adv. Mater.* **30**, 1801347, (2018).
9. Gorur, Y. C., Larsson, P. A. & Wågberg, L. Self-Fibrillating Cellulose Fibers: Rapid In Situ Nanofibrillation to Prepare Strong, Transparent, and Gas Barrier Nanopapers. *Biomacromolecules* **21**, 1480-1488, (2020).
10. Rao, J. *et al.* Strong Holocellulose-Based Nanopaper with a Sandwich-Like Structure for Effective Electromagnetic Shielding. *ACS Sustainable Chem. Eng.* **10**, 11396-11405, (2022).
11. Cao, W. *et al.* Ultrathin and Flexible CNTs/MXene/Cellulose Nanofibrils Composite Paper for Electromagnetic Interference Shielding. *Nano-Micro Lett.* **11**, 72, (2019).
12. Wang, S. *et al.* Transparent, Anisotropic Biofilm with Aligned Bacterial Cellulose Nanofibers. *Adv. Funct. Mater.* **28**, 1707491, (2018).
13. Shi, X. *et al.* Scalable production of carboxylated cellulose nanofibres using a green and recyclable solvent. *Nat. Sustain.* **7**, 315-325, (2024).
14. Dai, X. *et al.* High transmittance, high haze, and UV-harvesting CNNs@CNF/PVA composite film for light management. *Adv. Compos. Hybrid Mater.* **7**, 234, (2024).
15. Chen, J. *et al.* Rapid Dissolving-Debonding Strategy for Optically Transparent Paper Production. *Sci. Rep.* **5**, 17703, (2015).
16. Aaen, R., Simon, S., Wernersson Brodin, F. & Syverud, K. The potential of TEMPO-oxidized cellulose nanofibrils as rheology modifiers in food systems. *Cellulose* **26**, 5483-5496, (2019).
17. Yang, W., Jiao, L., Liu, W., Deng, Y. & Dai, H. Morphology control for tunable optical properties of cellulose nanofibrils films. *Cellulose* **25**, 5909-5918, (2018).
18. Zhu, M. *et al.* Anisotropic, Transparent Films with Aligned Cellulose Nanofibers. *Adv. Mater.* **29**, 1606284, (2017).
19. Li, G., Yu, D., Song, Z., Wang, H. & Liu, W. Reducing formation time while improving transparency and strength of cellulose nanostructured paper with polyvinylpyrrolidone and Laponite. *Carbohydr. Polym.* **230**, 115580, (2020).
20. Jeon, Y. *et al.* Lignin-tailored lignocellulose nanofiber films for light management using a deep eutectic solvent. *Chem. Eng. J.* **520**, 165989, (2025).

21. Koskela, S. *et al.* An Oxidative Enzyme Boosting Mechanical and Optical Performance of Densified Wood Films. *Small* **19**, 2205056, (2023).
