# Supplementary material for: Living co-culture fabrication for biologically crosslinked mycelium–cellulose hydrogels and films
Source: Nat Commun. 2026 Aug 24;17:10134. doi: 10.1038/s41467-026-76430-6 (PMC13601560; doi:10.1038/s41467-026-76430-6)
Supplement: Supplementary file 2 [file 41467_2026_76430_MOESM2_ESM.pdf]

nature portfolio

Corresponding author(s): Tian Li

Last updated by author(s): Jul 15, 2026

Reporting Summary

Nature Portfolio wishes to improve the reproducibility of the work that we publish. This form provides structure for consistency and transparency in reporting. For further information on Nature Portfolio policies, see our Editorial Policies and the Editorial Policy Checklist.

Statistics

For all statistical analyses, confirm that the following items are present in the figure legend, table legend, main text, or Methods section.

n/a	Confirmed	
☐	☒	The exact sample size (n) for each experimental group/condition, given as a discrete number and unit of measurement
☐	☒	A statement on whether measurements were taken from distinct samples or whether the same sample was measured repeatedly
☒	☐	The statistical test(s) used AND whether they are one- or two-sided<br>*Only common tests should be described solely by name; describe more complex techniques in the Methods section.*
☒	☐	A description of all covariates tested
☐	☒	A description of any assumptions or corrections, such as tests of normality and adjustment for multiple comparisons
☐	☒	A full description of the statistical parameters including central tendency (e.g. means) or other basic estimates (e.g. regression coefficient) AND variation (e.g. standard deviation) or associated estimates of uncertainty (e.g. confidence intervals)
☒	☐	For null hypothesis testing, the test statistic (e.g. F, t, r) with confidence intervals, effect sizes, degrees of freedom and P value noted<br>*Give P values as exact values whenever suitable.*
☒	☐	For Bayesian analysis, information on the choice of priors and Markov chain Monte Carlo settings
☒	☐	For hierarchical and complex designs, identification of the appropriate level for tests and full reporting of outcomes
☒	☐	Estimates of effect sizes (e.g. Cohen's d, Pearson's r), indicating how they were calculated

Our web collection on statistics for biologists contains articles on many of the points above.

Software and code

Policy information about availability of computer code

Data collection	FTIR data were collected using Nicolet 6700 and Nicolet iS50 fourier transform infrared spectroscopy; XPS data were collected using Kratos X-ray photoelectron spectroscopy; SEM images were collected using Apreo 2S microscopy equipped with a Quorum Cryo-prep system; EDS data were collected using a Helios G4 UX dual-beam system; TGA data were collected using SDT Q600 thermogravimetry; surface roughness data was collected using Asylum Cypher atomic force microscopy; SAXS data was collected using SAXS point 2.0 small-angle X-ray scattering; tensile test data were collected using ADMET and CellScale mechanical testers; optical data were collected using a UV-2600 spectrophotometer; radiative cooling data were collected using a K-type thermocouple (Omega 5TC-TT-K-30-72) and LabVIEW 2019. Infrared images were obtained using E8-XT infrared thermal camera. The pore fraction data were collected using ImageJ v1.54g. All schematic illustrations were created using Autodesk 3ds Max 2018. Chemical structures were created using ChemDraw 21.0.0. Figure panels were assembled using Microsoft PowerPoint 2021.
Data analysis	EDS data were analyzed using Aztec 6.3. SAXS data were analyzed using SAXS analysis 4.1. AFM images were analyzed using Gwyddion 2.70. Optical simulation was performed using COMSOL 6.0. XPS data were analyzed by Avantage 6.6.0. Graphs were generated using Origin 2025. Data calculation was performed by Microsoft Excel 2021.

For manuscripts utilizing custom algorithms or software that are central to the research but not yet described in published literature, software must be made available to editors and reviewers. We strongly encourage code deposition in a community repository (e.g. GitHub). See the Nature Portfolio guidelines for submitting code & software for further information.

Data

Policy information about availability of data

All manuscripts must include a data availability statement. This statement should provide the following information, where applicable:

- Accession codes, unique identifiers, or web links for publicly available datasets
- A description of any restrictions on data availability
- For clinical datasets or third party data, please ensure that the statement adheres to our policy

The data that support the findings of this study are available from the corresponding author upon request. Source data are provided.

Research involving human participants, their data, or biological material

Policy information about studies with human participants or human data. See also policy information about sex, gender (identity/presentation), and sexual orientation and race, ethnicity and racism.

Reporting on sex and gender	This study does not involve human participants or human data. Therefore, sex and gender informations are not included.
Reporting on race, ethnicity, or other socially relevant groupings	This study does not involve human participants or human-derived data. Therefore, reporting on race, ethnicity, or other socially relevant groupings is not included.
Population characteristics	Population characteristics was not considered in the study design. Therefore, no such information has been collected or included in this study.
Recruitment	N/A
Ethics oversight	N/A

Note that full information on the approval of the study protocol must also be provided in the manuscript.

Field-specific reporting

Please select the one below that is the best fit for your research. If you are not sure, read the appropriate sections before making your selection.

☒ Life sciences ☐ Behavioural & social sciences ☐ Ecological, evolutionary & environmental sciences

For a reference copy of the document with all sections, see nature.com/documents/nr-reporting-summary-flat.pdf

Life sciences study design

All studies must disclose on these points even when the disclosure is negative.

Sample size	No statistical method was used to predetermine sample size. Sample sizes were chosen based on established practices in previous similar studies and included at least three independently grown samples to ensure the reproducibility of the findings.
Data exclusions	No data were excluded from the analyses.
Replication	All experiments were repeated independently at least three times with consistent results, confirming the reproducibility of the findings.
Randomization	Randomization was not relevant to this study, as no experimental groups requiring random allocation were involved.
Blinding	Blinding was not relevant to this study, as no subjective assessment or group allocation was involved during data collection or analysis.

Reporting for specific materials, systems and methods

We require information from authors about some types of materials, experimental systems and methods used in many studies. Here, indicate whether each material, system or method listed is relevant to your study. If you are not sure if a list item applies to your research, read the appropriate section before selecting a response.

Materials & experimental systems

n/a	Involved in the study
☒	☐ Antibodies
☒	☐ Eukaryotic cell lines
☒	☐ Palaeontology and archaeology
☒	☐ Animals and other organisms
☒	☐ Clinical data
☒	☐ Dual use research of concern
☒	☐ Plants

Methods

n/a	Involved in the study
☒	☐ ChIP-seq
☒	☐ Flow cytometry
☒	☐ MRI-based neuroimaging

Plants

Seed stocks: N/A

Novel plant genotypes: N/A

Authentication: N/A
