# Supplementary material for: Living co-culture fabrication for biologically crosslinked mycelium–cellulose hydrogels and films
Source: Nat Commun. 2026 Aug 24;17:10134. doi: 10.1038/s41467-026-76430-6 (PMC13601560; doi:10.1038/s41467-026-76430-6)
Supplement: Supplementary file 3 [file 41467_2026_76430_MOESM3_ESM.pdf]

nature portfolio

Peer Review File

Living Co-culture Fabrication for Biologically Crosslinked Mycelium–Cellulose Hydrogels and Films

Corresponding Author: Professor Tian Li

This file contains all reviewer reports in order by version, followed by all author rebuttals in order by version.

Version 0:

Reviewer comments:

Reviewer #1

(Remarks to the Author)
This manuscript presents a novel biological co-culture approach in which fungal mycelium and bacterial cellulose form hierarchical hydrogels and robust transparent films. The authors aim to demonstrate a process–structure–property relationship driven entirely by microbial biological activity, which is an important and emerging direction for sustainable materials design. The work is promising, but several mechanistic and experimental issues should be addressed to fully substantiate the claims and strengthen the manuscript.
1. The mechanistic basis of “biological crosslinking” requires further clarification. The manuscript attributes network formation to “biological crosslinking,” but the mechanistic origin of this process is not sufficiently explained. The authors should clarify which specific microbial activities drive the formation and strengthening of the mycelium–cellulose interface. It would also be helpful to indicate whether measurable biological or chemical changes occur during co-culture that distinguish biological crosslinking from simple physical entanglement or hydrogen bonding.
2. Process–structure–property relationship. The novelty of this work lies in demonstrating that microbial activity directly programs material structure and functionality. However, the manuscript does not clearly articulate how growth kinetics and co-culture dynamics translate into hierarchical fiber assembly, pore formation, interfacial bonding, and ultimately mechanical or optical properties. A more explicit framework connecting biological processes, microstructure and macroscopic properties is needed.
3. The manuscript states that the MBC hydrogel can sustain a 1.5 kg load without rupture, but no tensile strength or related mechanical parameters are reported. Quantitative mechanical measurements are required to substantiate this claim.
4. The authors attribute haze enhancement to multiscale refractive index mismatches, including air pores created by hyphal collapse. However, pore size distribution, density, and whether pores are primarily nanoscale or microscale are not reported. Since optical tunability is a major contribution of this work, pore morphology should be quantified and explicitly linked to scattering behavior.
5. The unexpectedly high transparency of pure mycelium films requires further mechanistic explanation. The manuscript reports approximately 80% transparency for pure mycelium films despite the presence of microscale hyphae. The structural or optical mechanisms responsible for this unexpectedly high transparency should be clarified.
6. The manuscript describes the films as biodegradable but does not provide experimental evidence. Since the work compares MBC films to other biodegradable materials, at least preliminary degradation data (e.g., soil burial tests) should be included.
7. The XPS discussion identifies functional groups but does not specify which material components contribute to each peak or whether co-culture induces discernible spectral shifts. A more rigorous and component-specific interpretation is needed.
8. The manuscript should clarify whether hydrogel healing behavior is dominated by continued biological growth or by reversible physical interactions. These mechanisms have different implications for material performance.
9. Scale bars are missing in Fig. 3a and 3b and should be added.
10. Terminology should be used consistently throughout the manuscript. Terms such as “biological crosslinking,” “co-assembly,” and “interfacial bonding” should be clearly defined and used with precision.
11. Drying conditions should be reported clearly, as optical properties depend strongly on drying rate, humidity and temperature. These parameters should be specified.

Reviewer #2

(Remarks to the Author)
With the advancing field of Engineered Living Materials, living fabrication has become a promising strategy for producing novel programmable materials. Several co-culture studies have been reported to generate functional bacterial cellulose materials. Here, the authors report a novel composite material with controllable optical properties produced by co-culturing white rot fungi spores with cellulose-producing bacteria. The authors conducted a wide range of material characterisation in terms of mechanical properties of this composite film and thorough investigation of optical properties. Potentially, this new material could have a wide range of applications. However, there are vital flaws in the current manuscript. Although the innovation has potential value, it requires major revision before it can be accepted for publication.

Major Concerns:
The primary concern with the current manuscript is the lack of detail in the methods section. Critical information is missing, including: the specific fungal strain used, how fungal spores were produced, how cellulose fermentation was set up, the inoculation ratio between spores and cellulose-producing bacteria, and the dimensions and volumes of the fermentation reactors used in each experimental setup. All of these details have direct impacts on growth dynamics and material properties. Without this information, property comparisons between materials are not valid, experiments cannot be replicated, and results cannot be confidently interpreted or concluded.

In addition, many other critical details are missing, such as the thickness of films harvested at different time periods, porosity, and surface roughness. All of this information impacts the properties claimed in the paper. Without in-depth investigation of these parameters, the conclusions are not convincing. The number of replicates for each test and statistical analysis methods should also be required in the methods section.

Finally, the authors claim that MBC films are ideal for applications as coatings on outdoor displays. There is always a challenge for biodegradable materials to balance degradability and durability for outdoor applications. These properties are not necessarily in conflict, but the authors should demonstrate consideration of overall property requirements for outdoor applications to support this claim.

Minor Issues:
Line 25: Please avoid acronyms in the abstract if possible, or define them before use.
Line 33: While this statement is true, microorganisms can also make inert materials that do not self-heal. Consider using the term "Engineered Living Material" more precisely.
Line 38: I am not sure this paper has explored regulation of living materials sufficiently. Perhaps this point should be moved to the discussion.
Line 45: "Acetobacter" is an outdated name for this organism. Please update it with the latest scientific name (Komagataeibacter).
Line 49: Can the authors please provide a reference?
Line 54: Could the authors briefly explain what nacre is?
Line 55: Instead of "butterfly shows," consider "butterflies show."
Line 85: Could the authors please explain this hydrogel more fully and explain how it differs from mycelium, which is well known to form on the surface of liquid cultures? In fact, many references in this paper rely on the known fact that mycelium forms a layer on liquid surfaces, and Agrawal et al., 2025 in JOM has also reported mycelium hydrogels.
Figure 2i: The annotation (highlighted hyphae region) is misleading; please revise it.
Figure S5: Could the authors add scale bars to these microscope images?
Line 142: Although Fig. 3b shows 'self-healing' properties, there is no explanation in the text of how this was achieved. This is important as it demonstrates the mechanism for healing.
Line 144: References 29-30 are inappropriate, as the mechanisms in those papers are very different from what is presented here.
Line 145: From the images, it appears that only hyphae are growing. Could the authors explain how they identified new growth of both BC and mycelium? There is an absence of bacterial cells in the image, which are necessary to produce more BC, unlike mycelium which is itself alive.
Lines 212-227: Transmittance and haze of films often depend on thickness, fibril density, porosity, and surface roughness. Without investigating these properties, only presenting percentages is weak and unconvincing. Additionally, how repeatable are these data? In the literature, unmodified BC usually shows much lower transmittance than what the authors present here. Could you discuss this discrepancy?
Line 283: Adjusting culture duration will undoubtedly impact film thickness, and film thickness has a direct and strong impact on scattering intensity. Attributing scattering intensity changes only to hyphae and interlamellar pores provides only a partial explanation. The authors should consider all other elements that changed and their impacts.
Figure 5g: Could the authors explain why the thermocouple was positioned next to the heater rather than on the glass surface?
Line 347: Can the authors explain succinctly how they measured the increase in mycelium (and how this was attributed to mycelium rather than BC)? Please provide a percentage increase.
Line 357: Please use italics for microorganism names.
Line 357: Please provide the name of the seller on Etsy.
Line 358: Please use italics for microorganism names.
Line 367: Please use italics for microorganism names.
Line 367: Did your media contain any glucose? If not, can the authors explain how they achieved any BC production?
Line 368: Please provide details on how the fungi and bacteria were prepared for co-culture and how much of each was added.
Line 370: Could you please state the temperature range for "room temperature" in your geographical location?

Line 371: Were all films treated with NaOH? If so, can the authors explain how any BC could grow during breakage and self-healing without any living cells to produce it? And how did the mycelium survive this treatment?
Line 373: Can the authors please provide details on the air-drying procedure, i.e., how long were samples air-dried, and how was the endpoint determined?
Line 394: Please reread this sentence for clarity.

Reviewer #3

(Remarks to the Author)
I co-reviewed this manuscript with one of the reviewers who provided the listed reports. This is part of the Nature Communications initiative to facilitate training in peer review and to provide appropriate recognition for Early Career Researchers who co-review manuscripts.

Version 1:

Reviewer comments:

Reviewer #1

(Remarks to the Author)
The authors have addressed all my concerns.

Reviewer #2

(Remarks to the Author)
The authors have made a good effort to address the initial comments and the further tests and images provided in the revised manuscript are very welcome. There are still some areas that require more detail in both methods and results, and some conclusions which are not supported by the evidence provided.
Major concerns:
1. Authors claim that their MBC hydrogel is distinct from previously reported mycelium hydrogels due to the absence of an extensive hyphal network, but they do not provide a mechanistic explanation for this observation. Given that hyphal network formation is a well-known characteristic of fungal growth, the authors should discuss in depth why the co-culture conditions suppress hyphal network development. This explanation is critical to substantiate their claim of a unique co-culture system and would significantly strengthen the process–structure–property relationship that is central to this work.
2. Additionally, P. chrysosporium is known to be a very efficient degrader of cellulose. The authors should address why this fungus has been chosen and why it did not metabolise the bacterial cellulose in this co-culture system, and provide a mechanistic discussion of how the BC matrix was preserved despite the presence of a known cellulolytic organism. This is a fundamental question that has not been adequately addressed in the current revision.
3. The authors' response regarding the inoculation ratio remains unsatisfactory. Authors sated that a fixed spore-to-bacteria numerical ratio cannot be precisely defined due to the dynamic nature of the cultures, simply reporting a volumetric ratio (3 mL spores : 3 mL bacteria : 10 mL medium) is insufficient to ensure reproducibility and rigorous interpretation of the results. In standard microbiology practice, inocula are routinely standardised using quantitative measures such as OD600, CFU/mL, or spore counts per mL at the time of inoculation, regardless of the dynamic nature of the cultures. Without such standardisation, it is unclear whether the reported material properties are reproducible across different batches and different lab conditions. The actual ratio of fungal to bacterial biomass introduced into the co-culture system could vary significantly between experiments. The authors should provide at least one quantitative measure of the starting biomass concentration for both the fungal spore suspension and the bacterial culture, and discuss how batch-to-batch variation in inoculum concentration may affect the resulting material properties.
4. The methods section still lacks critical details regarding the pre-culture conditions for both organisms. The authors should specify: the temperature, agitation speed, volume of media, and duration for which each pre-culture was grown or maintained; whether a new pre-culture was prepared for each experiment or whether it was maintained continuously; and whether 3 mL of liquid culture was used directly, or whether mycelium mats or BC pellicles were added instead. If pellicles were used, the authors must explain how this translates to a consistent 3 mL volume across replicates.
5. Authors have added soil burial experiments in response to the biodegradability concern, which is welcomed. But the evidence presented in Supplementary Fig. 5 raises questions about experimental consistency. Specifically, the soil texture and appear noticeably different across the three timepoints (initial, 2 weeks, and 4 weeks), which makes it difficult to confirm that the same samples are being monitored throughout the experiment. In a rigorous soil burial degradation study, it is standard practice to track the same samples continuously under identical environmental conditions, with consistent imaging conditions including fixed camera angle, lighting, and distance. The authors need to clarify whether the images presented represent the same experimental batch monitored over time, or whether different soil batches or containers were used at each timepoint. In addition, to strengthen the biodegradability claims, the authors should supplement the visual observations with quantitative measurements such as mass or area loss percentage at each timepoint, which would provide more convincing and reproducible evidence of degradation. The inclusion of scale bars in the images would also improve the clarity and rigor of this figure. The authors should also clarify whether the samples used for biodegradability testing were treated with NaOH. If not, we suspect we should see mycelium growth. If so, this is inconsistent with the self-healing experiments where living cells are required, and this distinction should be made explicit in the manuscript.
Minor concerns:
1. The authors should explain why the air-dried samples on glass plates (Fig 3d and Fig S8) appear considerably more

transparent than those dried without glass (Fig 5). It is unclear whether this difference is due to film thickness, surface contact effects during drying, or other factors. This discrepancy should be addressed explicitly.
2. The TGA graph presented in Fig 4f does not appear sufficient to definitively determine or calculate hyphal content based on height or pore size. The authors should consider revising the manuscript to reflect this limitation. Alternative approaches such as EDX analysis (which may allow a crude estimate based on nitrogen presence, given that chitin is nitrogen-rich) or through the use of specific cellulose or mycelium fluorescent dyes could provide more rigorous quantification of the relative contributions of each component
3. The text states that “MBC films with 4-day and 6-day culture durations exhibit only a few scattered hyphae, while the MBC8D sample shows relatively higher hyphal content.” However, in Fig S15, the thickness measurements are very similar for the 6-day and 8-day samples, and the porosity measurements from Fig S16 show only modest differences. The authors should explain more rigorously how these structural measurements support the conclusion that 8-day cultivation results in significantly higher hyphal content. Furthermore, the ImageJ porosity analysis may reflect pore spaces caused by wrinkling during drying rather than definitively being caused by hyphal incorporation, and this limitation should be acknowledged.
4. Fig 4f: It does not look possible to determine hyphal content from this TGA graph, nor is it possible to calculate this based on height or pore size. Could the authors consider revising the manuscript to reflect this limitation? An EDX analysis may allow a crude estimate based on nitrogen presence. Or through use of a specific cellulose or mycelium dye.
5. Line 162: Where is the evidence for hydrogen bond formation? There is still no evidence that the fibres shown are “new” fibres, specifically the BC.
6. The HS medium as described in the manuscript appears to be prepared at half strength compared to the standard Hestrin-Schramm formulation (Glucose: 20 g/L, Yeast Extract: 5 g/L, Peptone: 5 g/L, Disodium Phosphate: 2.7 g/L, Citric Acid: 1.15 g/L). The authors should clarify whether this was intentional and provide justification. Additionally, it should be stated whether the medium was titrated to a specific pH prior to inoculation, as pH has a well-known effect on both BC production and fungal growth.
7. The methods for the self-healing experiments are insufficiently described. The authors should clarify: how the samples were fractured; whether and how the samples were washed prior to rejoining; how long the samples were grown before fracture; and how long the healing period was before assessment. These details are essential for reproducibility and for interpreting the self-healing results.

Reviewer #3

(Remarks to the Author)
I co-reviewed this manuscript with one of the reviewers who provided the listed reports. This is part of the Nature Communications initiative to facilitate training in peer review and to provide appropriate recognition for Early Career Researchers who co-review manuscripts.

Version 2:

Reviewer comments:

Reviewer #2

(Remarks to the Author)
The authors have addressed most of my concerns. One minor change is required: the figure 4f should be a barplot rather than a line graph as there is no relationship across all the samples so the points shouldn't be joined up

Reviewer #3

(Remarks to the Author)
I co-reviewed this manuscript with one of the reviewers who provided the listed reports. This is part of the Nature Communications initiative to facilitate training in peer review and to provide appropriate recognition for Early Career Researchers who co-review manuscripts.

Open Access This Peer Review File is licensed under a Creative Commons Attribution 4.0 International License, which permits use, sharing, adaptation, distribution and reproduction in any medium or format, as long as you give appropriate credit to the original author(s) and the source, provide a link to the Creative Commons license, and indicate if changes were made.
In cases where reviewers are anonymous, credit should be given to 'Anonymous Referee' and the source.
The images or other third party material in this Peer Review File are included in the article's Creative Commons license, unless indicated otherwise in a credit line to the material. If material is not included in the article's Creative Commons license and your intended use is not permitted by statutory regulation or exceeds the permitted use, you will need to obtain permission directly from the copyright holder.
To view a copy of this license, visit https://creativecommons.org/licenses/by/4.0/

Response Letter for NCOMMS-25-92975

Manuscript Information

Manuscript ID: NCOMMS-25-92975

Title: Living Co-culture Fabrication for Biologically Crosslinked Mycelium–Cellulose Hydrogels and Films

Authors: Bingyu Xia[1], Yanpei Tian[1], Qilong Cheng[1], Huilong Liu[1], Xiaojie Liu[1], Zhenyuan Niu[1], Xiwei Shan[1], Pengfei Deng[1], Wenhui Xu[1], Shubhra Bansal[1,2], Tian Li[1]*

*Correspondence: tianli@purdue.edu

Reviewers' comments:

Reviewer #1: *This manuscript presents a novel biological co-culture approach in which fungal mycelium and bacterial cellulose form hierarchical hydrogels and robust transparent films. The authors aim to demonstrate a process–structure–property relationship driven entirely by microbial biological activity, which is an important and emerging direction for sustainable materials design. The work is promising, but several mechanistic and experimental issues should be addressed to fully substantiate the claims and strengthen the manuscript.*

Response: We sincerely thank Reviewer #1 for the thorough and constructive review of our manuscript. We appreciate the positive assessment of our work and have carefully addressed all comments, making corresponding revisions as detailed below. We have added some content in the Results and Discussion to clarify the process–structure–property relationship and performed additional experiments to demonstrate the biodegradability of the film and the tensile properties of the hydrogel.

Comment 1: *The mechanistic basis of "biological crosslinking" requires further clarification. The manuscript attributes network formation to "biological crosslinking," but the mechanistic origin of this process is not sufficiently explained. The authors should clarify which specific microbial activities drive the formation and strengthening of the mycelium–cellulose interface. It would also be helpful to indicate whether measurable biological or chemical changes occur during co-culture that distinguish biological crosslinking from simple physical entanglement or hydrogen bonding.*

Response: Thanks for raising this important point. The biological crosslinking in this work does not refer to chemical changes. Instead, it describes a growth-driven, biologically mediated interfacial integration process that occurs during co-culture of bacteria and fungi. Hyphal growth occurs primarily at the apical tip through continuous cell wall synthesis and vesicle-mediated transport. Tip elongation and branching progressively generate a three-dimensional mycelial network. At the same time, bacterial cells continuously synthesize and secrete cellulose nanofibers into the extracellular space. As hyphae extend and branch, these cellulose fibers are deposited between and around the growing hyphae. The simultaneous hyphal growth

and cellulose biosynthesis lead to a co-evolving, interpenetrating three-dimensional network stabilized by hydrogen bonding and physical interlocking. The corresponding revisions have been incorporated into the manuscript. Besides, FTIR was used to characterize the hydrogen bonding formation between hyphae and BC. The corresponding content in the manuscript is detailed below.

"During the co-culture process, hyphae elongated primarily at the apical tip through continuous cell wall synthesis and vesicle-mediated transport. At the same time, bacterial cells continuously synthesize and secrete cellulose nanofibers into the extracellular space. As hyphae extend and branch, these cellulose fibers are deposited between and around the growing hyphae, leading to an interpenetrating three-dimensional network stabilized by hydrogen bonding and physical interlocking." (Page 6, Line 96)

"As shown in Fourier transform infrared spectroscopy (FTIR) spectrum, BC exhibits a broad peak centered at 3340 cm^{-1}, corresponding to the O–H stretching vibration, shifting to 3263 cm^{-1} in the MBC sample. This red shift indicates enhanced hydrogen bonding between BC and mycelial hyphae due to interfacial interactions." (Page 7, Line 117)

Comment 2: *Process–structure–property relationship. The novelty of this work lies in demonstrating that microbial activity directly programs material structure and functionality. However, the manuscript does not clearly articulate how growth kinetics and co-culture dynamics translate into hierarchical fiber assembly, pore formation, interfacial bonding, and ultimately mechanical or optical properties. A more explicit framework connecting biological processes, microstructure and macroscopic properties is needed.*

Response: We thank the reviewer for these insightful comments. During the co-culture process, hyphae elongated primarily at the apical tip through continuous cell wall synthesis and vesicle-mediated transport. At the same time, bacterial cells continuously synthesize and secrete cellulose nanofibers into the extracellular space. As hyphae extend and branch, these cellulose fibers are deposited between and around the growing hyphae, leading to a co-evolving, interpenetrating three-dimensional network stabilized by hydrogen bonding and physical

interlocking. Upon drying, MBC hydrogel was transformed into a flat film with a layer-stacked structure of hyphae and BC nanofibers as shown in the SEM images (Figs. 2k-n). The initial tubular hyphae collapsed into flattened, ribbon-like filaments due to the loss of turgor pressure, leading to the formation of interlamellar pores within the MBC films (Fig. 2n). The content of hyphae and interlamellar macropores can be increased by extending culture durations, which increases the number of interfaces with large RI contrast. The resulting RI mismatch further enhances Mie scattering, leading to higher haze values. Moreover, evaporation-induced consolidation reduced inter-fiber spacing and brought hydroxyl-rich chains into denser compaction, which further enhanced the interfacial interaction via hydrogen bonding. We have added the corresponding discussions to the revised manuscript as detailed below.

"As hyphae extend and branch, these cellulose fibers are deposited between and around the growing hyphae, leading to an interpenetrating three-dimensional network stabilized by hydrogen bonding and physical interlocking." (Page 6, Line 99)

"Furthermore, evaporation-induced consolidation reduced inter-fiber spacing and brought hydroxyl-rich chains into denser compaction, which further enhanced the interfacial interaction via hydrogen bonding." (Page 8, Line 146)

"Moreover, by regulating culture duration, the film thickness, hyphal content and interlamellar pore structure can be tuned, enabling programmable optical scattering: the haze can be modulated from 16.0% to 78.1% while maintaining ~80% transmittance." (Page 18, Line 384)

Comment 3: *The manuscript states that the MBC hydrogel can sustain a 1.5 kg load without rupture, but no tensile strength or related mechanical parameters are reported. Quantitative mechanical measurements are required to substantiate this claim.*

Response: We have acted on this suggestionand performed quantitative tensile testing of the MBC hydrogel, with results reported below.. As shown in Supplementary Fig. 6, the average tensile strength and elongation of the MBC hydrogel after four measurements are 1.27 ± 0.12 MPa and 48.47 ± 1.48 %, respectively. The corresponding content was also added in the manuscript as detailed below.

"The tensile strength and elongation of the MBC hydrogel are 1.27 ± 0.12 MPa and 48.47 ± 1.48 %, respectively (Supplementary Fig. 6)." (Page 9, Line 160)

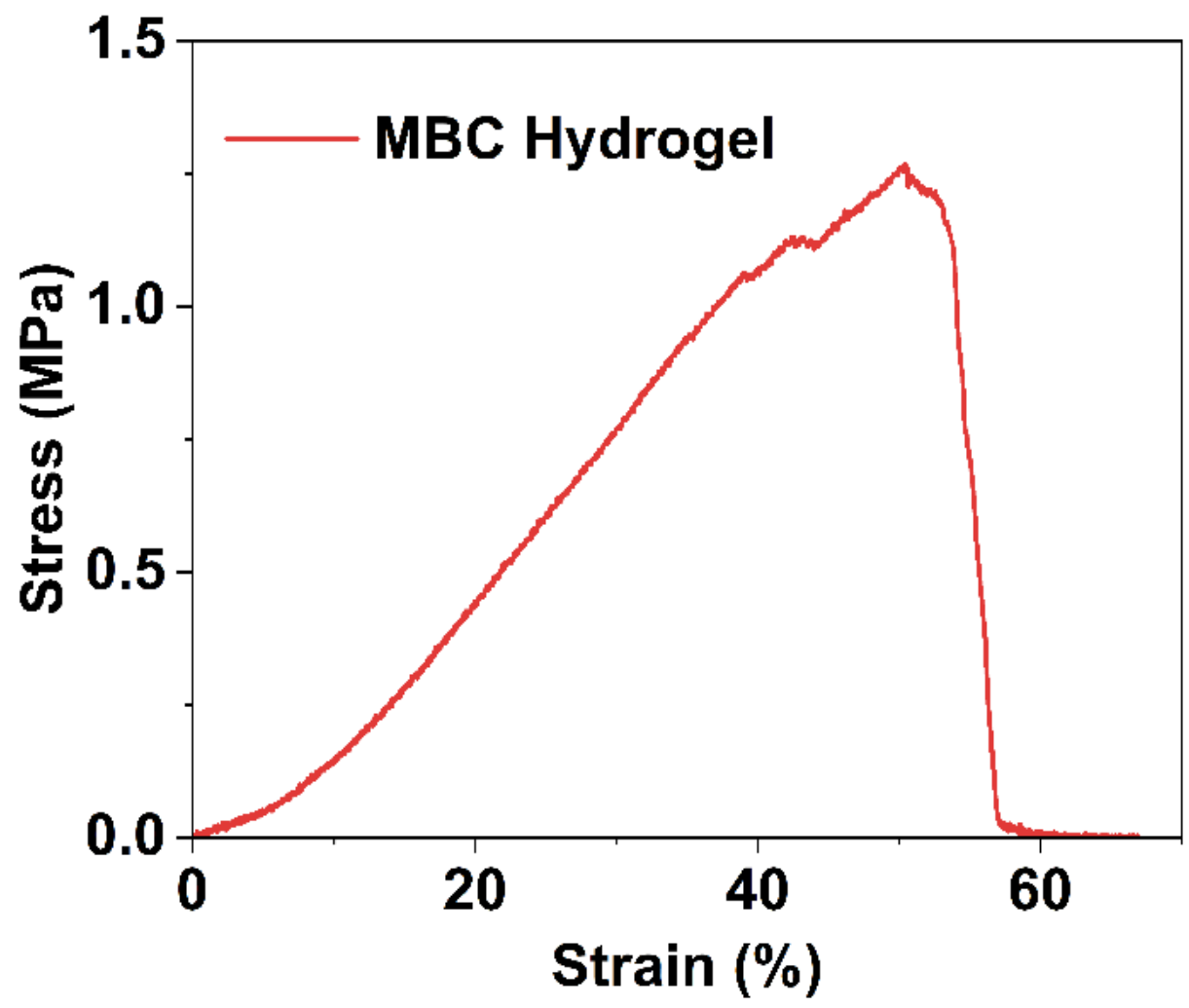

Supplementary Fig. 6. Tensile properties of MBC hydrogels. Strain-stress curves of the MBC hydrogel.

Comment 4: *The authors attribute haze enhancement to multiscale refractive index mismatches, including air pores created by hyphal collapse. However, pore size distribution, density, and whether pores are primarily nanoscale or microscale are not reported. Since optical tunability is a major contribution of this work, pore morphology should be quantified and explicitly linked to scattering behavior.*

Response: We appreciate this important observation. Pore morphology indeed plays a critical role in light scattering. As observed in the SEM images (Fig. 2n), the pores in the MBC films are primarily at the macroscale, which originate from the deformation and collapse of hyphae upon the loss of turgor pressure during drying. These macroscale pores introduce strong refractive index contrast and are expected to dominate light scattering. Porosity plays an important role in light scattering. However, smaller pores (below ~100 nm), which are difficult to resolve by SEM, are expected to contribute less to light scattering. According to Supplementary Note 2, in the Rayleigh scattering regime ($r \ll \lambda$), the scattering efficiency increases with particle size as $Q_{sca} \propto (r/\lambda)^4$. Pores with diameters much smaller than the wavelength of visible light contribute only weakly to the overall scattering intensity compared

to macropores. Therefore, to quantify the pore structure, ImageJ was employed to analyze the pore area fraction in the cross-section. The pore area fraction increases with increasing culture time. As shown in Supplementary Fig. 16, increasing culture time leads to the incorporation of more mycelial hyphae and the formation of corresponding macropores within the BC matrix. These macropores act as effective scattering centers due to the refractive index (RI) contrast, in agreement with the optical simulation results (Fig. 4j). We have added the content in the revised manuscript as detailed below.

"The increase of hyphal content was also observed from the cross-section of the films. As shown in Supplementary Fig. 15, MBC films with 4-day and 6-day culture durations exhibit only a few scattered hyphae, while the MBC8D sample shows relatively higher hyphal content. Furthermore, the pore area fraction in the cross-section of the MBC films also increase due to the incorporation of more hyphae in the BC matrix (Supplementary Fig. 16). These macropores act as effective light scattering centers due to the RI contrast between air and matrix." (Page 14, Line 290)

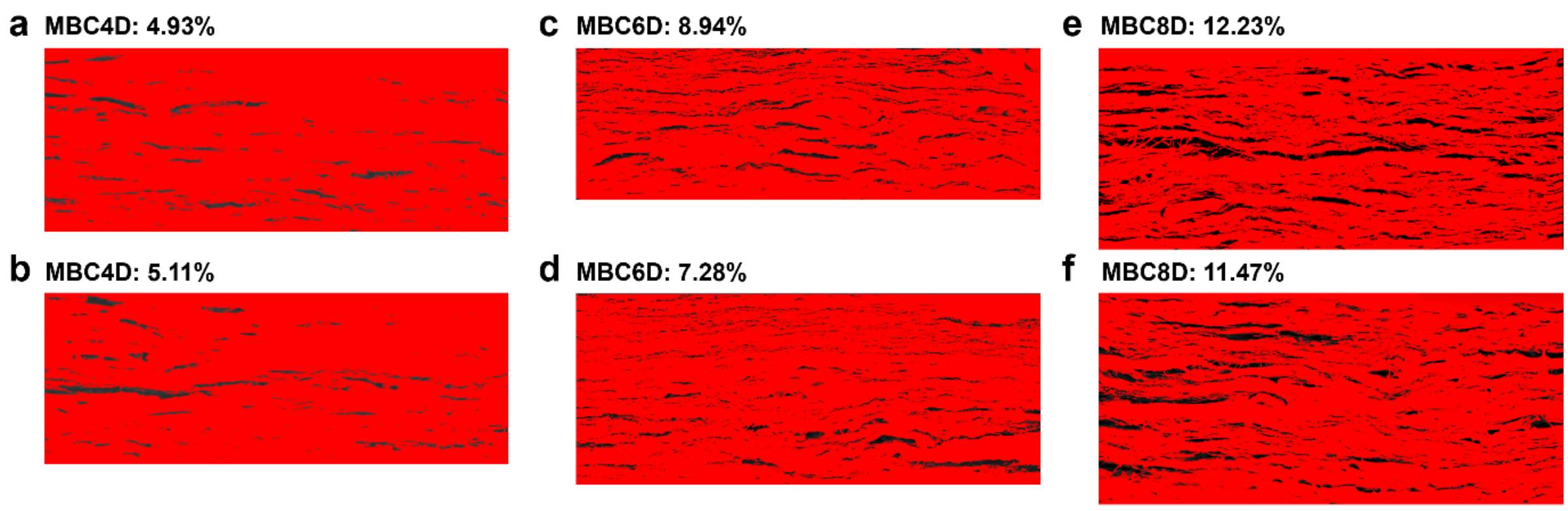

Supplementary Fig. 16. Porous structure of MBC films. Cross-sectional porosity analysis of MBC films with different culture durations using ImageJ.

Comment 5: *The unexpectedly high transparency of pure mycelium films requires further mechanistic explanation. The manuscript reports approximately 80% transparency for pure mycelium films despite the presence of microscale hyphae. The structural or optical mechanisms responsible for this unexpectedly high transparency should be clarified.*

Response: This is an excellent point. During the air-drying process of the pure mycelium

hydrogel, the initially tubular hyphae collapsed into flattened, ribbon-like filaments due to the loss of turgor pressure. These ribbon-like hyphae subsequently stacked closely in a layered manner, forming a dense and compact structure, as shown in Fig. 3h. The compact structure reduced the inter-fiber voids, thereby minimizing air-filled gaps within the film. As a result, the refractive index mismatch between the hyphae and the surrounding medium decreased, leading to reduced light scattering and enhanced optical transparency. The corresponding content in the manuscript is shown below.

"In pure MY films, the diameters of hyphae are comparable to visible wavelengths, giving rise to Mie scattering. The Mie scattering efficiency, as expressed in Supplementary Note 3 and 4, increases with the RI contrast between the fibers and the surrounding medium ($Q_{sca} \propto n_f/n_m$)[1]. However, as observed in the cross-sectional SEM images, the MY film also exhibits a uniform and dense morphology with minimal RI difference (Supplementary Fig. 12b), leading to low scattering efficiency." (Page 12, Line 255)

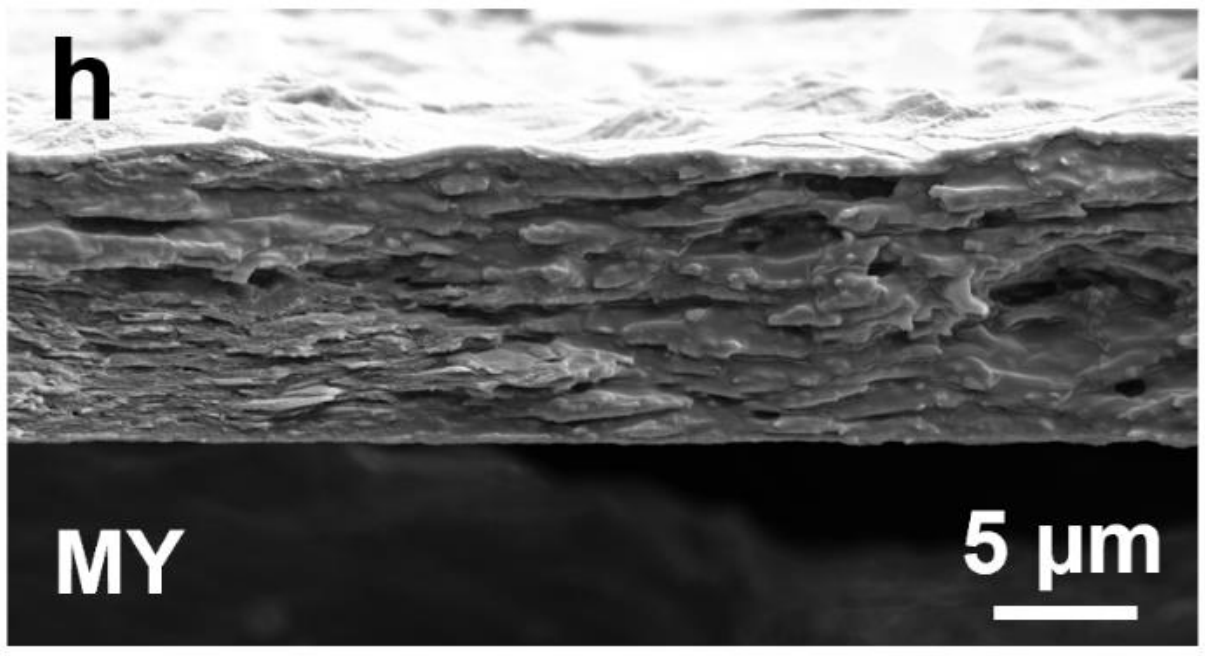

Fig. 3h. SEM images of tensile fracture surfaces of pure MY film.

Comment 6: *The manuscript describes the films as biodegradable but does not provide experimental evidence. Since the work compares MBC films to other biodegradable materials, at least preliminary degradation data (e.g., soil burial tests) should be included.*

Response: We agree that experimental evidence for biodegradability is needed. The biodegradability of the MBC and MY films was therefore evaluated by soil burial tests. As shown in Supplementary Fig. 5, both the MY film and the MBC film exhibited visible wrinkles and structural damage after 2 weeks of burial, and were further degraded into small fragments after 4 weeks. These observations demonstrate the good biodegradability of both mycelium

hyphae and bacterial cellulose under natural soil conditions. Soil burial tests and the corresponding description have been added to the revised manuscript and the Supplementary Information, as detailed below.

"The biodegradability of the MBC film and MY film was evaluated by burying the samples in soil. As shown in Supplementary Fig. 5, both the MY film and the MBC film exhibited visible wrinkles and structural damage after 2 weeks of burial, and were further degraded into small fragments after 4 weeks. These observations demonstrate the favorable biodegradability of mycelium and bacterial cellulose under natural soil conditions." (Page 7, Line 128)

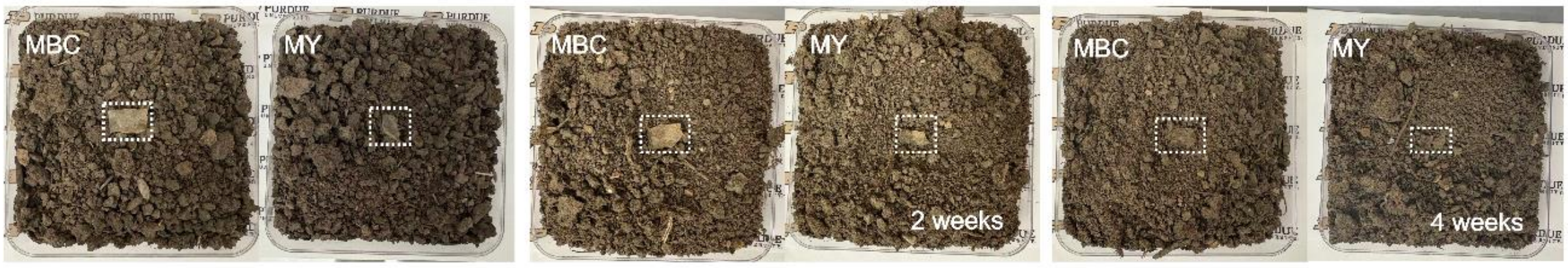

Supplementary Fig. 5. Biodegradability of pure MY and MBC films. Soil burial biodegradation behavior of MBC and MY films.

Comment 7: *The XPS discussion identifies functional groups but does not specify which material components contribute to each peak or whether co-culture induces discernible spectral shifts. A more rigorous and component-specific interpretation is needed.*

Response: We thank the reviewer for this valuable suggestion. As shown in **Fig. R1**, the peaks in C 1s and O 1s spectrum are mainly consistent with the polysaccharide-rich cell wall structure of mycelium, including β-glucans and mannoproteins, as well as chitin-associated carbonyl groups. Additionally, the N 1s spectrum displays a single peak at 400.00 eV, mainly arising from the amide groups (HN–C=O) of chitin. The corresponding content has been added to the revised manuscript, as detailed below.

"The C 1s spectrum can be divided into four peaks at 284.80, 286.45, 287.95, and 289.05 eV, corresponding to C–C/C=C, C–O, C=O, and O–C=O, respectively (Fig. 2a). These peaks are consistent with the polysaccharide-rich cell wall structure of mycelium, including β-glucans and mannoproteins, as well as chitin-associated carbonyl groups." (Page 6, Line 107)

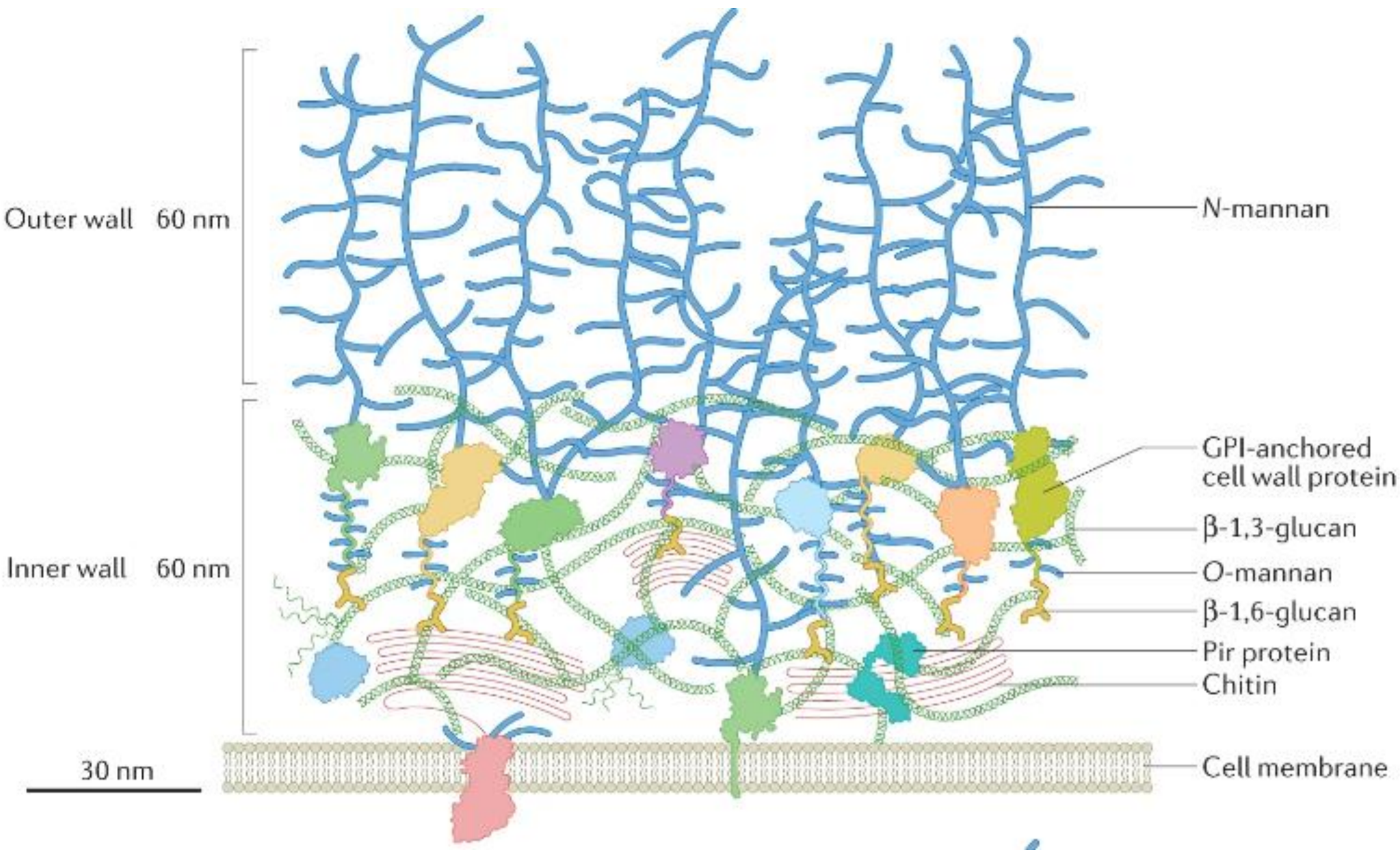

Fig. R1. Architecture of the fungal cell wall[2].

Comment 8: *The manuscript should clarify whether hydrogel healing behavior is dominated by continued biological growth or by reversible physical interactions. These mechanisms have different implications for material performance.*

Response: Thanks for your comment. The healing behavior of the hydrogel arises from the combined contributions of continued biological growth and reversible hydrogen-bond-mediated physical interactions. Immediately after mechanical damage, the rapid recovery of mechanical properties is primarily governed by reversible hydrogen bonding and physical re-entanglement within the network. At longer timescales, continued biological growth of both mycelial hyphae and bacterial cellulose contributes to structural reinforcement by forming new physical entanglements and additional hydrogen-bonding sites (Supplementary Fig. 7). Therefore, the healing process involves an initial physically driven recovery followed by biologically mediated network reinforcement. We have added the discussion in the revised manuscript, as detailed below.

"When the fractured surfaces were brought back into contact, hyphae and BC re-entangled at interface and hydrogen bonds between them were re-established. These interactions enable the reconstruction of the interfacial network and restore the mechanical integrity of the MBC film,

resulting in the observed self-healing behavior.” (Page 9, Line 162)

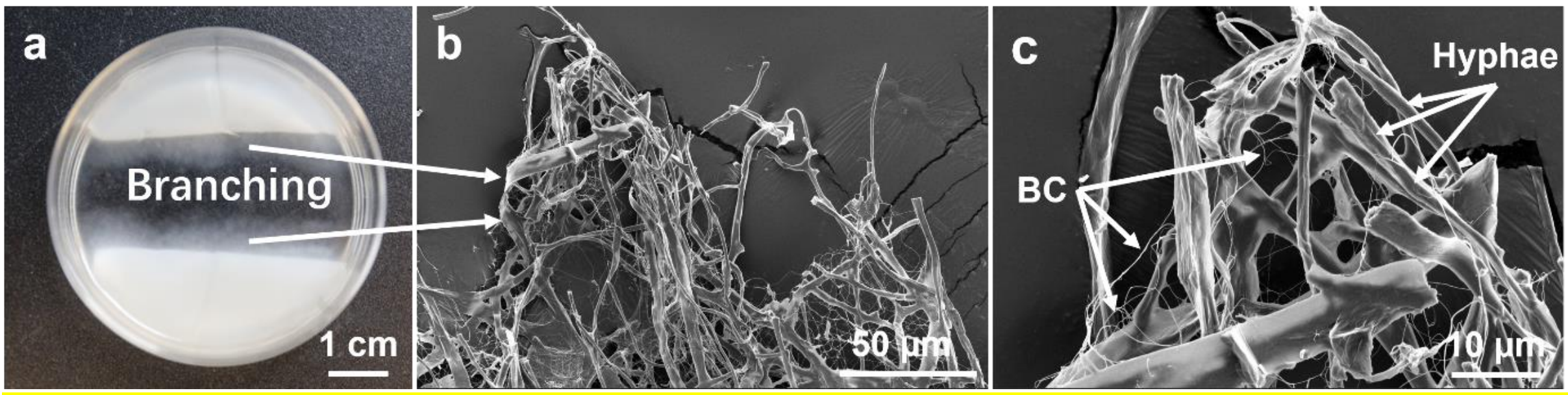

Supplementary Fig. 7. Self-healing and re-growth of MBC hydrogels. a Photographs showing the self-healing and re-growth of MBC hydrogel. **b, c** SEM images of the newly grown thin layer composed of hierarchical BC nanofibers and hyphae.

Comment 9: *Scale bars are missing in Fig. 3a and 3b and should be added.*

Response: Thanks for your comment. Scale bars have been added to Fig. 3a and 3b in the revised manuscript, as shown below.

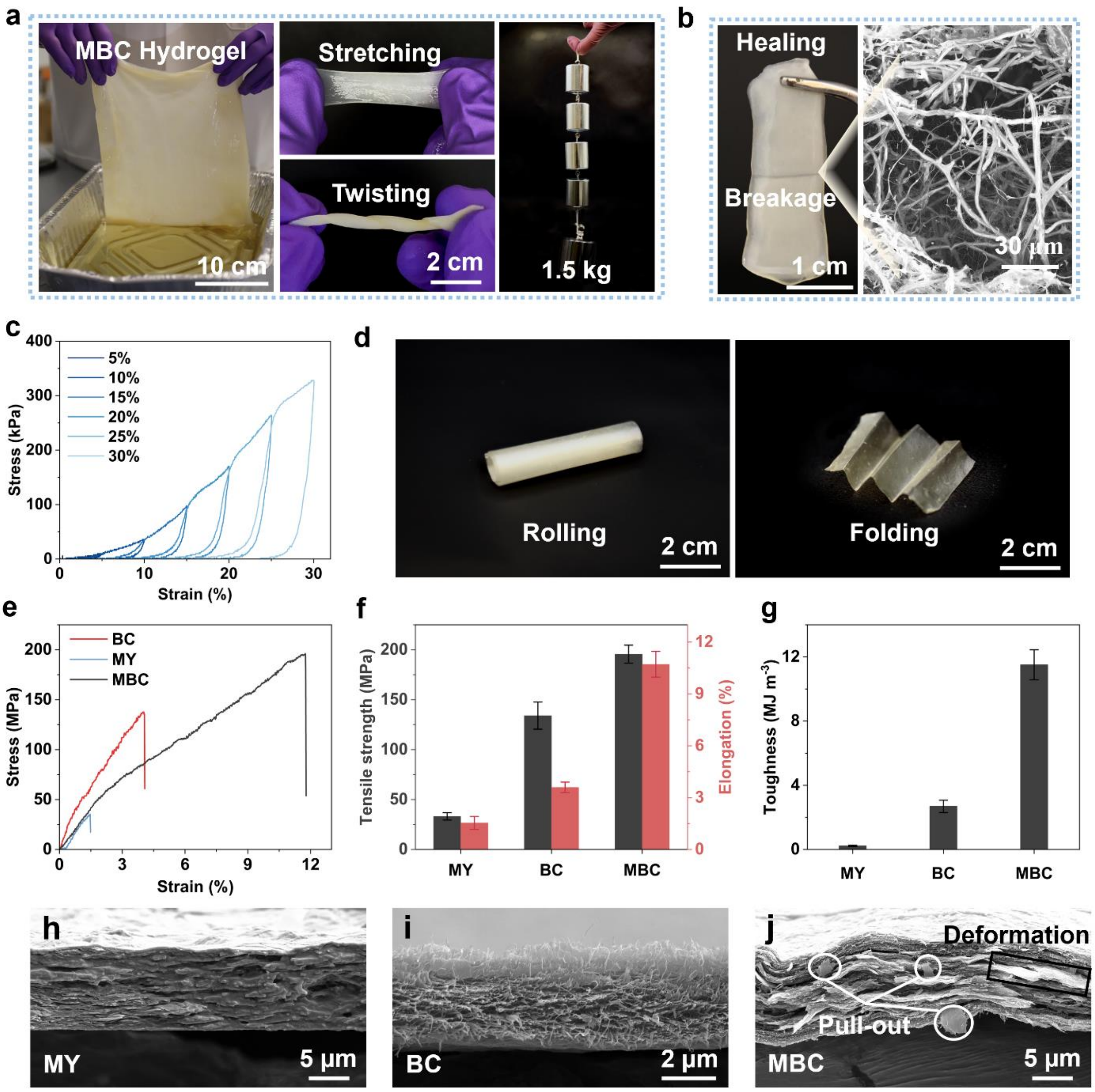

Fig. 3. Mechanical properties of MBC hydrogels and films. a Photographs showing MBC hydrogels and their tensile properties. **b** Photographs of self-healing behavior of MBC hydrogels and SEM images of the breakage. **c** Loading-unloading cyclic tensile stress-strain curves of MBC hydrogel. **d** Photographs of the rolled and folded MBC films. **e** Stress-strain curves of MY, BC and MBC films. **f** Comparison of the tensile strength and elongation of MY, BC and MBC films. **g** Comparison of the toughness of MY, BC and MBC films. **h-j** SEM images of tensile fracture surfaces of MY, BC and MBC films. Fiber pull-out is marked by white circles, and plastic deformation is highlighted by black rectangles.

Comment 10: *Terminology should be used consistently throughout the manuscript. Terms such as "biological crosslinking," "co-assembly," and "interfacial bonding" should be clearly defined and used with precision.*

Response: We thank the reviewer for this valuable suggestion. The terminology has been revised throughout the manuscript to ensure consistency and precision. Specifically, we have clearly defined the term "biological crosslinking" to describe the network formation arising from concurrent biological growth and in situ interpenetration, rather than covalent chemical crosslink formation. Co-assembly refers to the simultaneous growth process of hyphae and BC nanofibers. The term "interfacial bonding" was replaced by "interfacial hydrogen bonding" throughout the manuscript to avoid misunderstanding.

"Microscale fungal mycelium (MY) and nanoscale BC fibers in liquid media to form hierarchically structured hydrogels through biological crosslinking (i.e., the combined effect of physical entanglement and interfacial hydrogen bonding between hyphae and BC nanofibers)" (Page 3, Line 61)

Comment 11: *Drying conditions should be reported clearly, as optical properties depend strongly on drying rate, humidity and temperature. These parameters should be specified.*

Response: Thanks for your valuable comment. The drying process was completed under ambient laboratory conditions at room temperature (~23–25 °C) with a relative humidity of approximately 30% for 12 hours. These parameters have now been specified in the revised manuscript to ensure clarity and reproducibility. We have added these parameters in the Methods section, as detailed below.

"Finally, the hydrogels were air-dried at room temperature with a relative humidity of approximately 30% for 12 hours to obtain films." (Page 19, Line 419)

Reviewer #2: *With the advancing field of Engineered Living Materials, living fabrication has become a promising strategy for producing novel programmable materials. Several co-culture studies have been reported to generate functional bacterial cellulose materials. Here, the authors report a novel composite material with controllable optical properties produced by co-culturing white rot fungi spores with cellulose-producing bacteria. The authors conducted a wide range of material characterization in terms of mechanical properties of this composite film and thorough investigation of optical properties. Potentially, this new material could have a wide range of applications. However, there are vital flaws in the current manuscript. Although the innovation has potential value, it requires major revision before it can be accepted for publication.*

Response: We sincerely thank Reviewer #2 for the thorough review and constructive suggestions. In response, we have added detailed experimental procedures to the Materials and Methods section — including strain preparation, culture conditions, and processing steps — to ensure improved clarity and reproducibility. In addition, we have performed a series of additional experiments to further characterize the thickness, pore structure, and surface roughness of the MBC films with different culture durations. These additional analyses provide deeper insight into the evolution of the hierarchical structure and offer stronger support for the interpretation of the tunable optical properties. We believe that these revisions have significantly improved the rigor and completeness of the manuscript.

Major concerns:

Comment 1: *The primary concern with the current manuscript is the lack of detail in the methods section. Critical information is missing, including: the specific fungal strain used, how fungal spores were produced, how cellulose fermentation was set up, the inoculation ratio between spores and cellulose-producing bacteria, and the dimensions and volumes of the fermentation reactors used in each experimental setup. All of these details have direct impacts on growth dynamics and material properties. Without this information, property comparisons between materials are not valid, experiments cannot be replicated, and results cannot be*

confidently interpreted or concluded.

Response:

We fully agree that detailed methodological information is essential for reproducibility and accurate interpretation of the results. We have addressed each missing item below.

(1) The specific fungal strain used. The fungal strain is *Phanerochaete chrysosporium*, a white-rot fungus purchased from Outgrow on Etsy. This information has been added to the Materials and Methods section (Page 19, Line 395).

(2) How fungal spores were produced. The fungal spores were cultured and maintained in a liquid medium containing 8 g L^{-1} glucose and 1.5 g L^{-1} peptone under sterile conditions. This culture served as the spore suspension inoculum for co-culture experiments. These details have been added to the Methods section (Page 19, Line 402).

(3) How cellulose fermentation was set up. *K. xylinus* was maintained in a liquid medium containing 5 g L^{-1} yeast extract, 3 g L^{-1} peptone, and 25 g L^{-1} mannitol. The HS medium for BC production was prepared by dissolving 10 g glucose, 2.5 g yeast extract, 2.5 g peptone, 2.7 g disodium phosphate anhydrous, and 1.5 g citric acid monohydrate in 1000 g deionized water. In addition, the co-culture medium (LM-HS) combines LM and HS media at a 7:3 volume ratio. All media were sterilized by autoclaving at 121 °C for 15 min. These details have been added to the Methods section (Page 19, Line 404).

(4) The inoculation ratio between spores and cellulose-producing bacteria. Because both the fungal spore suspension and the bacterial culture are introduced as liquid cultures whose biomass concentrations dynamically evolve during propagation, a fixed spore-to-bacteria numerical ratio cannot be precisely defined. Instead, inoculation was controlled by volume: approximately 3 mL of fungal spore suspension and 3 mL of bacterial culture were added to 10 mL of LM-HS medium, giving a volumetric inoculation ratio of 3:3:10 (spores : bacteria : fresh medium). This has been specified in the revised Methods section (Page 19, Line 411).

(5) Dimensions and volumes of the fermentation reactors. All co-culture experiments were

conducted in petri dishes with a diameter of 5 cm and a height of 1 cm, with a total liquid volume of 16 mL (3 mL spore suspension + 3 mL bacterial culture + 10 mL LM-HS medium), followed by static incubation for 4–10 days. This information has been added to the Methods section (Page 19, Line 413).

Summary: All five items raised by the reviewer — fungal strain identity, spore preparation, cellulose fermentation setup, inoculation ratio, and reactor dimensions — have been fully addressed and incorporated into the revised Materials and Methods section. The complete revised passage is provided below (Page 19, Line 402).

"First, the fungal spores purchased from Etsy were cultured and maintained in a liquid culture containing 8 g L^{-1} glucose and 1.5 g L^{-1} peptone under sterile conditions. The bacterial strain (*K. xylinus*) was cultured and maintained in a liquid culture containing 5 g L^{-1} yeast extract, 3 g L^{-1} peptone, and 25 g L^{-1} mannitol. Besides, the liquid medium for MY hydrogel (LM medium) was prepared by dissolving 10 g malt extract, 5 g yeast extract in 1000 g deionized water. The liquid medium for BC hydrogel (HS medium) was prepared by dissolving 10 g glucose, 2.5 g yeast extract, 2.5 g peptone, 2.7 g disodium phosphate anhydrous and 1.5 g citric acid monohydrate in 1000 g deionized water. The co-culture medium (LM-HS) is the combination of LM and HS medium with a volume ratio of 7:3. All the liquid medium was sterilized by autoclaving at 121 °C for 15 min. For the co-culture of mycelium and BC, approximately 3 mL of fungal spore suspension and 3 mL of cellulose-producing bacterial culture were added into 10 mL of LM-HS medium in a petri dish (diameter: 5 cm; height: 1 cm), followed by static incubation for 4–10 days to allow hydrogel formation. All procedures were conducted under sterile conditions. All cultures were incubated under room temperature (~23–25 °C) for desired culture duration (4 days, 6 days and 8 days)." (Page 19, Line 402)

Comment 2: *In addition, many other critical details are missing, such as the thickness of films harvested at different time periods, porosity, and surface roughness. All of this information impacts the properties claimed in the paper. Without in-depth investigation of these parameters, the conclusions are not convincing. The number of replicates for each test and statistical*

analysis methods should also be required in the methods section.

Response: We agree that film thickness, porosity, and surface roughness are all important factors influencing light scattering, and we have addressed them in detail. The increased haze value and slightly decreased transmittance of MBC films are primarily **governed by the increased thickness and the presence of macropores, while the contribution from surface roughness is negligible** due to its subwavelength scale. The corresponding discussion was added in the revised manuscript, as detailed below.

(1) The surface roughness of the film. MBC films exhibit a relatively smooth surface as shown in Fig. 2l and Fig. R2, which may originate from the collapse of initially tubular hyphae into flattened, ribbon-like filaments upon drying due to the loss of turgor pressure. The surface roughness of the MBC films was characterized by AFM (Supplementary Fig. 17). **The root mean square (RMS) roughness values for films cultured for 4, 6, and 8 days were 37.18, 37.60, and 44.34 nm, respectively.** Although a slight increase is observed, the RMS roughness remains much smaller than the wavelength of visible light, indicating that the surface is optically smooth. According to the optical scattering relation described in Supplementary Note 5, light scattering from surface roughness is governed by the ratio between the RMS roughness (σ) and the wavelength of incident light (λ)[3]. **Since all the roughness values remain substantially below the wavelength range of visible light (400–800 nm), indicating that the surface roughness of the MBC film has a negligible effect on its haze and transmittance properties.**

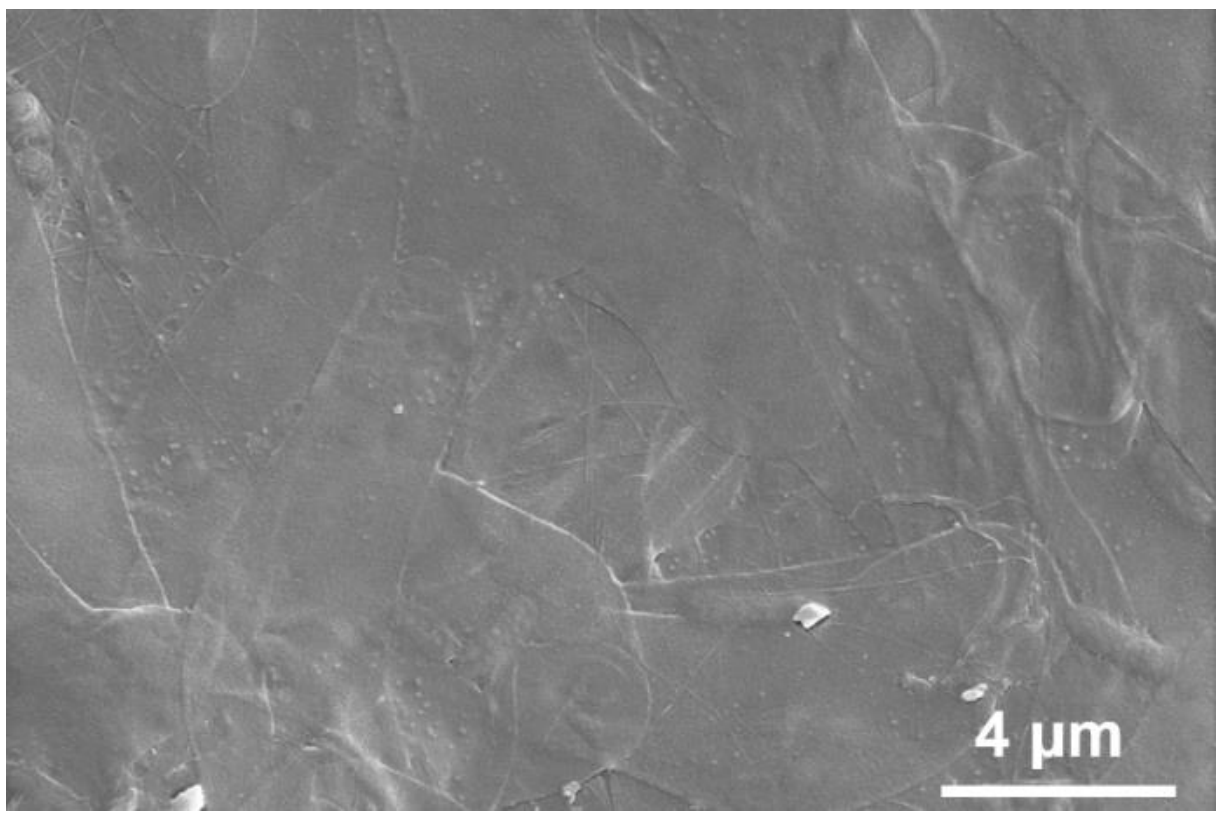

Fig. R2. Surface SEM images of MBC film.

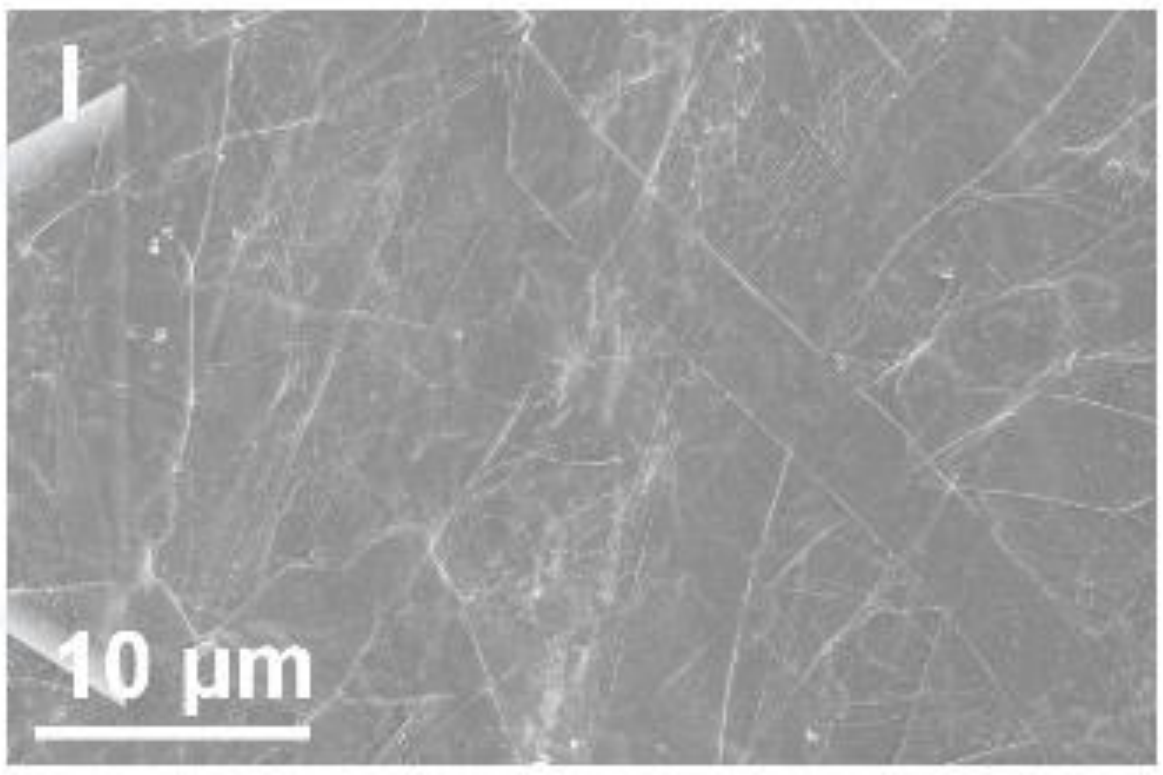

Fig. 2l. Surface SEM images of MBC film.

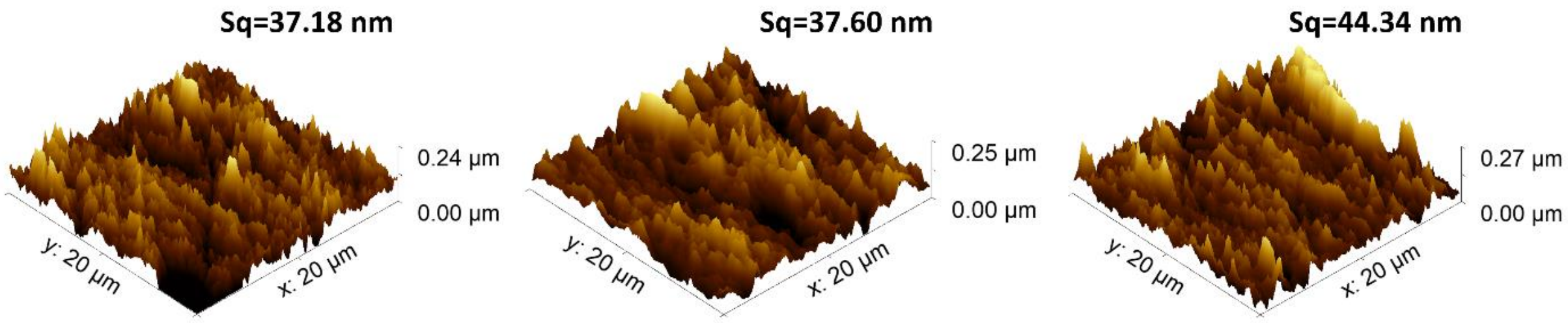

Supplementary Fig. 17. Surface structures of MBC films. Root mean square (RMS) roughness of MBC films. Sq is the RMS roughness.

Supplementary Note 5

$$TIS = 1 - exp\left[-\left(\frac{4\pi\sigma\cos\theta_i}{\lambda}\right)^2\right] \tag{5}$$

where TIS is the total integrated scattering, θ_i is the incident angle, λ is the laser wavelength, σ is the root mean square (RMS) roughness.

(2) **The thicknesses of MBC films with different culture durations** are **7.85 ± 0.22 μm (4 days), 8.96 ± 0.24 μm (6 days) and 9.64 ± 0.44 μm (8 days), respectively.** The thickness increases with increasing culture time due to the introduction of more mycelial hyphae and interlamellar pores (Supplementary Fig. 15). This structural evolution enhances light scattering **by increasing the optical path length** within the hierarchical network, which also slightly reduces the optical transmittance.

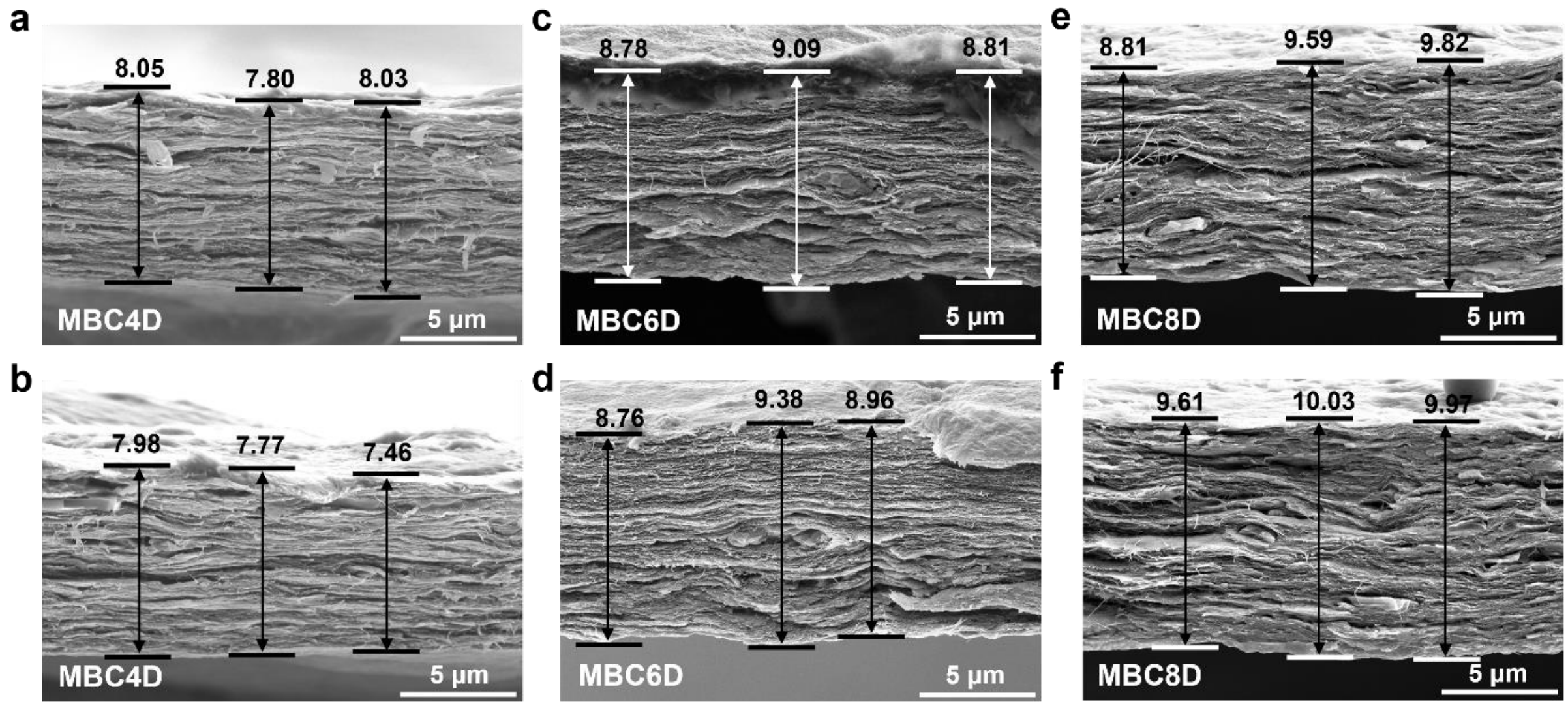

Supplementary Fig. 15. Thicknesses of MBC films. a-f Cross-sectional SEM images of MBC films with different culture durations (4 days, 6 days and 8 days).

(3) **The porosity of the film.** Porosity also plays an important role in the haze and transmittance properties of MBC films. However, smaller pores (below ~100 nm), which are difficult to resolve by SEM, are expected to contribute less to light scattering. According to Supplementary Note 2, in the Rayleigh scattering regime ($r \ll \lambda$), the scattering efficiency increases with particle size as $Q_{sca} \propto (r/\lambda)^4$. Pores with diameters much smaller than the wavelength of visible light contribute only weakly to the overall scattering intensity compared to macropores. Therefore, to quantify the pore structure, ImageJ was employed to analyze the pore area fraction in the cross-section. As shown in Supplementary Fig. 16, **the pore area fraction increases from 5.02% to 11.85% with increasing culture time.** The incorporation of more mycelial hyphae leads to the formation of corresponding macropores within the BC matrix. These macropores act as effective scattering centers due to the refractive index (RI) contrast, in agreement with the optical simulation results (Fig. 4j). **The resulting RI mismatch enhances Mie scattering and interfacial reflections at the interfaces among BC nanofibers, hyphae, and air pores, thereby increasing the haze value while slightly decreasing the transmittance (Supplementary Notes 1 and 3).**

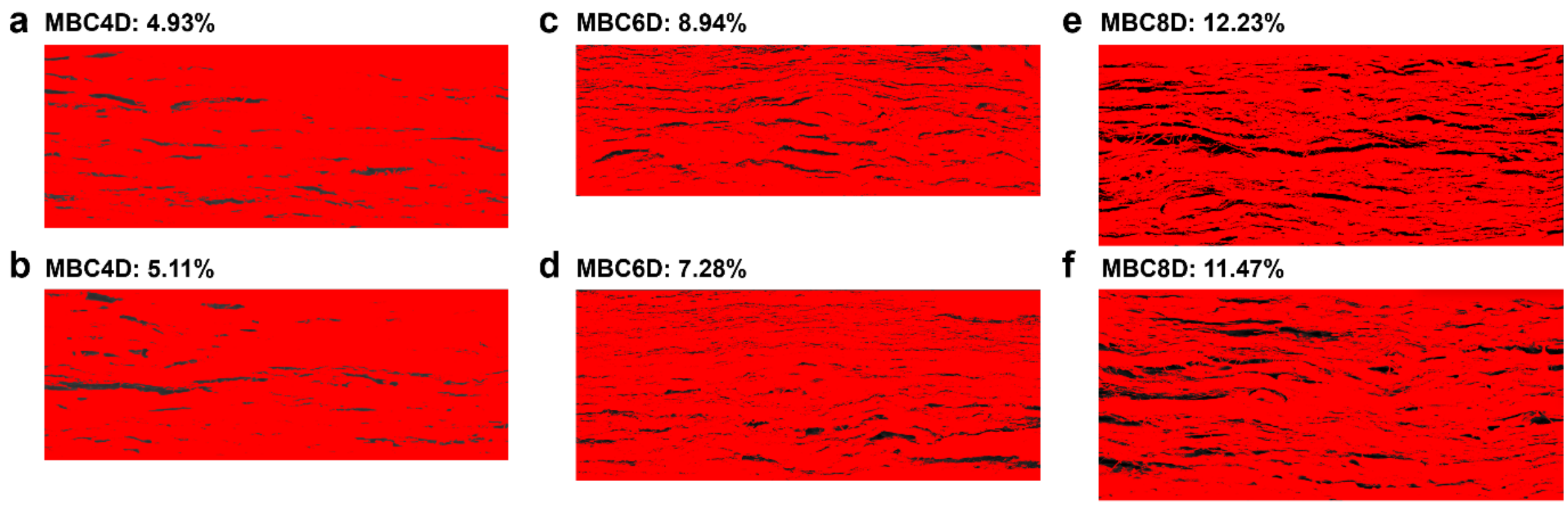

Supplementary Fig. 16. Porous structure of MBC films. Cross-sectional porosity analysis of MBC films with different culture durations using ImageJ.

The corresponding discussion was added in the revised manuscript, as detailed below.

"The increase of hyphal content was also observed from the cross-section of the films. As shown in Supplementary Fig. 15, MBC films with 4-day and 6-day culture durations exhibit only a few scattered hyphae, while the MBC8D sample shows relatively higher hyphal content. Furthermore, the pore area fraction estimated in the cross-section of the MBC films also increase from 5.02% to 11.85% due to the incorporation of more hyphae in the BC matrix (Supplementary Fig. 16). These macropores act as effective light scattering centers due to the RI contrast between air and matrix. The thicknesses of the MBC films cultured for different durations are 7.85 ± 0.22 μm (4 days), 9.10 ± 0.28 μm (6 days), and 9.64 ± 0.44 μm (8 days), respectively. The film thickness gradually increases with culture time, which can be attributed to the increase of hyphal content and the formation of more interlamellar pores. Additionally, the root mean square (RMS) roughness values for films cultured for 4, 6, and 8 days were 37.18, 37.60, and 44.34 nm, respectively (Supplementary Fig. 17). Although a slight increase is observed over the culture period, all values remain substantially below the wavelength range of visible light (400–800 nm), indicating that the surface of MBC films is optically smooth and the roughness has a negligible effect on light scattering (Supplementary Note 5)[49]." (Page 14, Line 290)

"More hyphae and interlamellar air pores are introduced into the MBC films with increasing culture duration, leading to an increased number of interfaces with large refractive index (RI)

contrast as well as greater film thickness. The increased film thickness further promotes light scattering by extending the optical path length within the hierarchical network. Moreover, the resulting RI mismatch enhances Mie scattering and interfacial reflections at the interfaces, thereby increasing the haze while slightly decreasing the transmittance (Supplementary Notes 1 and 3)." (Page 14, Line 314)

Regarding *"The number of replicates for each test and statistical analysis methods should also be required in the methods section."*

All the UV-vis and tensile tests were replicated at least 3 times. The statistical analysis methods for porosity, thickness and surface roughness have also been added to the Methods section, as detailed below.

"All experiments were conducted at a loading rate of 1 mm/min, and each sample was tested four times." (Page 20, Line 449)

"The transmittance and haze value at 550 nm of the films were used for comparison. Each kind of film was measured 3 times." (Page 21, Line 456)

"The pore fraction data was obtained from the SEM images using ImageJ software. Root mean surface roughness of the MBC film was obtained using an atomic force microscopy (AFM, Asylum Cypher, UK). The thicknesses of the MBC films were estimated from cross-sectional SEM images by measuring six different positions for each sample, and the average value was calculated." (Page 20, Line 436)

Comment 3: *Finally, the authors claim that MBC films are ideal for applications as coatings on outdoor displays. There is always a challenge for biodegradable materials to balance degradability and durability for outdoor applications. These properties are not necessarily in conflict, but the authors should demonstrate consideration of overall property requirements for outdoor applications to support this claim.*

Response: We agree that biodegradable materials must balance environmental degradability with sufficient durability for practical outdoor applications. Biodegradation of bio-based

materials typically occurs under biologically active conditions (e.g., soil or compost) where microorganisms and enzymes are present, whereas degradation rates are significantly slower under normal service conditions. These materials can therefore maintain functionality during use while providing environmental benefits at the end of their life cycle. The biodegradability of the MBC film and MY film was evaluated by burying the samples in soil. As shown in Supplementary Fig. 5, both the MY film and the MBC film exhibited **visible wrinkles and structural damage after 2 weeks of burial**, and were further **degraded into small fragments after 4 weeks**. These observations demonstrate the good biodegradability of both mycelium hyphae and bacterial cellulose under natural soil conditions. The corresponding content was added in the manuscript as detailed below.

"The biodegradability of the MBC film and MY film was evaluated by burying the samples in soil. As shown in Supplementary Fig. 5, both the MY film and the MBC film exhibited visible wrinkles and structural damage after 2 weeks of burial, and were further degraded into small fragments after 4 weeks. These observations demonstrate the favorable biodegradability of mycelium and bacterial cellulose under natural soil conditions." (Page 7, Line 128)

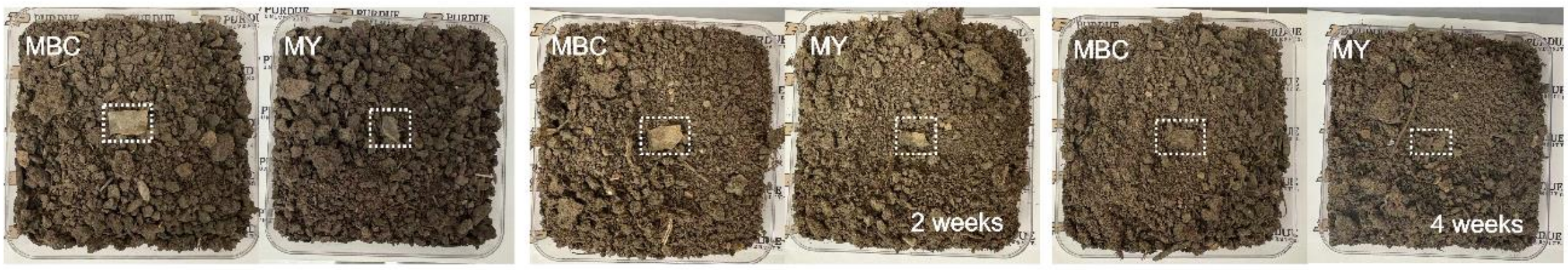

Supplementary Fig. 5. Biodegradability of pure MY and MBC films. Soil burial biodegradation behavior of MBC and MY films.

However, MBC films do not undergo degradation when used in outdoor environments, unlike when they are placed in biologically active environments such as soil. As shown in Supplementary Fig. 20, the MBC-coated glass exhibits good stability after 30 days of outdoor exposure. **No noticeable degradation is observed, and the MBC-coated glass still reduces the temperature of the heater surface by approximately 2.5 °C.** The corresponding content has been added to the revised manuscript, as detailed below.

We acknowledge that further material optimization may be required for specific outdoor

conditions. In practical implementations, the durability of MBC films can be further enhanced through surface or bulk modifications, which can improve resistance to moisture, UV exposure, and mechanical wear. Notably, the mycelial component contains abundant chemically active functional groups (e.g., hydroxyl, amine, and polysaccharide-rich moieties), which provide versatile reactive sites for further functionalization or crosslinking. These intrinsic features offer additional flexibility to tailor the balance between environmental degradability and service stability.

"In addition, the MBC-coated glass demonstrates good stability, showing no noticeable degradation after 30 days of outdoor exposure while still achieving a temperature reduction of ~2.5 °C on the heater surface (Supplementary Fig. 20)." (Page 17, Line 361)

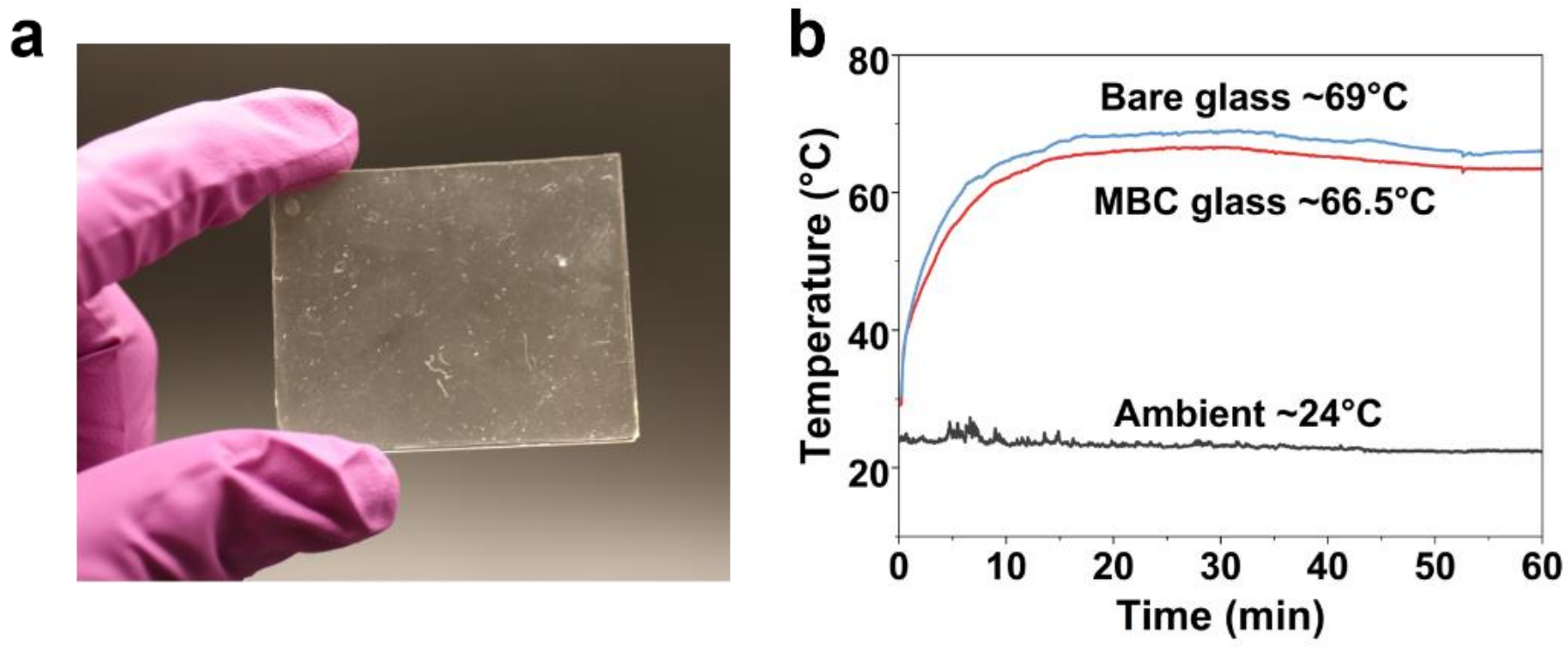

Supplementary Fig. 20. Durability of MBC glass after 30-day outdoor exposure. a Photograph of MBC glass after 30-day outdoor exposure. **b** Temperature of heater covered by bare glass and MBC glass under solar illumination.

Minor Issues:

We thank the reviewer for all the valuable suggestions and have made point-by-point revisions throughout the manuscript as detailed below.

Line 25: *Please avoid acronyms in the abstract if possible, or define them before use.*

Response: We have replaced the acronym "MBC" in the abstract with "Mycelium/bacterial" for improved clarity and readability. (Page 2, Line 25)

Line 33: *While this statement is true, microorganisms can also make inert materials that do not self-heal. Consider using the term "Engineered Living Material" more precisely.*

Response: We thank the reviewer for this valuable suggestion. We have clarified the use of the term "Engineered Living Material" in the revised manuscript to ensure it is used precisely and appropriately. (Page 3, Line 35)

Line 38: *I am not sure this paper has explored regulation of living materials sufficiently. Perhaps this point should be moved to the discussion.*

Response: We agree that the regulation of living materials is a critical aspect that warrants clearer articulation. In this work, the regulation of the living composite system is achieved through two complementary and tunable strategies.

First, by regulating the medium composition, we establish a co-culture liquid environment that simultaneously supports the growth of mycelium and the production of bacterial cellulose. This enables their co-development within a single system, leading to the formation of an interpenetrating hierarchical network. Such a design provides a fundamental level of regulation by controlling the coexistence and interaction between the two biological components.

Second, we introduce temporal control as an effective regulation parameter. By adjusting the culture duration, the relative content of mycelium and bacterial cellulose can be systematically tuned, which directly influences the porosity, density, and connectivity of the resulting network. This structural evolution further governs the optical properties of the composite films, enabling controllable modulation of light scattering and transparency.

Together, these strategies establish a simple yet versatile framework for regulating both the structure and properties of living composite materials through coupled biological and process parameters. Following the reviewer's suggestion, we have revised the manuscript to further clarify this regulation mechanism and expanded the relevant discussion to better highlight its significance. We have added the corresponding content in the Results and Discussion sections,

as detailed below.

"This structural tunability of MBC films can be directly reflected in their optical performance." (Page 14, Line 307)

"More hyphae and interlamellar air pores are introduced into the MBC films with increasing culture duration, leading to an increased number of interfaces with large refractive index (RI) contrast as well as greater film thickness. The increased film thickness further promotes light scattering by extending the optical path length within the hierarchical network. Moreover, the resulting RI mismatch enhances Mie scattering and interfacial reflections at the interfaces, thereby increasing the haze while slightly decreasing the transmittance (Supplementary Notes 1 and 3). By simply adjusting culture duration, MBC films achieve tunable optical haze across a wide range while preserving high transmittance, offering a scalable, bio-based strategy for tailoring light diffusion in applications." (Page 14, Line 315)

"Mycelium microfibers and BC nanofibers co-assemble within a designed liquid medium, enabling their simultaneous growth into an interpenetrating double network via biological crosslinking, which results in a strong, self-supporting hydrogel." (Page 18, Line 376)

"Moreover, by regulating culture duration, the film thickness, hyphal content and interlamellar pore structure can be tuned, enabling programmable optical scattering: the haze can be modulated from 16.0% to 78.1% while maintaining ~80% transmittance." (Page 18, Line 384)

Line 45: *"Acetobacter" is an outdated name for this organism. Please update it with the latest scientific name (Komagataeibacter).*

Response: We have updated the organism name to the currently accepted taxonomy throughout the manuscript. (Page 3, Line 47)

Line 49: *Can the authors please provide a reference?*

Response: We have added appropriate references to support the reported low stretchability of pure BC films. Previous studies have shown that the **elongation at break of pure BC films typically ranges from approximately 3–5%**[4-7]. These references have now been cited in the

revised manuscript. In comparison, the elongation at break of the MBC film in this work increased to 10.72%, which can be attributed to the hierarchical architecture formed by mycelial hyphae and BC nanofibers.

Line 54: *Could the authors briefly explain what nacre is?*

Response: We appreciate this question. Nacre (mother-of-pearl) isthe iridescent inner layer of mollusk shells and is widely recognized as a model natural structural material. It exhibits a highly ordered hierarchical "brick-and-mortar" architecture, in which stiff aragonite platelets (~95 vol%) are densely stacked and bonded by thin organic interlayers (~5 vol%) composed of proteins and polysaccharides. Despite being primarily composed of a brittle ceramic phase, nacre achieves an exceptional combination of strength and toughness due to its hierarchical organization across multiple length scales. This architecture enables various toughening mechanisms, including platelet sliding, crack deflection, and energy dissipation at organic–inorganic interfaces.

We have added a brief explanation of nacre (mother-of-pearl, a natural hierarchical composite composed of aragonite platelets and organic interfaces[8]) at its first appearance in the manuscript for clarity. (Page 3, Line 55)

Line 55: *Instead of "butterfly shows," consider "butterflies show."*

Response: We thank the reviewer for the careful reading. We have corrected this grammatical error, replacing "butterfly shows" with "butterflies show" in the manuscript. (Page 3, Line 57)

Line 85: *Could the authors please explain this hydrogel more fully and explain how it differs from mycelium, which is well known to form on the surface of liquid cultures? In fact, many references in this paper rely on the known fact that mycelium forms a layer on liquid surfaces, and Agrawal et al., 2025 in JOM has also reported mycelium hydrogels.*

Response: We appreciate this comment and would like to clarify that the hydrogel reported in this work is distinct from the mycelium layers commonly formed at the air–liquid interface. **In**

our work, mycelium forms an independent hydrogel in the liquid medium rather than developing a bilayer structure with an aerial layer, and can co-culture with bacterial cellulose (BC) to yield a previously unreported composite hydrogel.

In our system, mycelium and bacterial cellulose (BC) grow simultaneously at the liquid surface and form an interpenetrating composite hydrogel network. The fungal hyphae provide a microscale scaffold, while BC nanofibers are continuously produced by bacteria and interwoven with the hyphae to form a hierarchical structure. Therefore, the resulting material is a **mycelium/BC composite hydrogel rather than a hydrogel formed solely by mycelium.** After drying, this composite hydrogel can be converted into films with tunable structures and properties.

We also note that the structure reported by Agrawal et al. (JOM, 2025) differs from the hydrogel system in this work. In their study, the material exhibits a bilayer structure formed by mycelium. The submerged layer, formed in the liquid phase, can be considered hydrogel-like, whereas the aerial layer grows into the air and develops into a relatively thick mycelial network. This upper layer is not a hydrogel but rather a porous, foam-like structure composed of air-grown hyphae. In contrast, our system forms an independent hydrogel network at the liquid interface. We have added the corresponding content in the Introduction section, as detailed below.

"In contrast to the previous study[9], mycelium in this work forms an independent hydrogel in the liquid medium rather than developing a bilayer structure with an aerial layer, and can co-culture with bacterial cellulose (BC) to yield a previously unreported composite hydrogel." (Page 4, Line 63)

Figure 2i: *The annotation (highlighted hyphae region) is misleading; please revise it.*

Response: We agree that this annotation was potentially misleading and have removed the highlighted region from Fig. 2i.

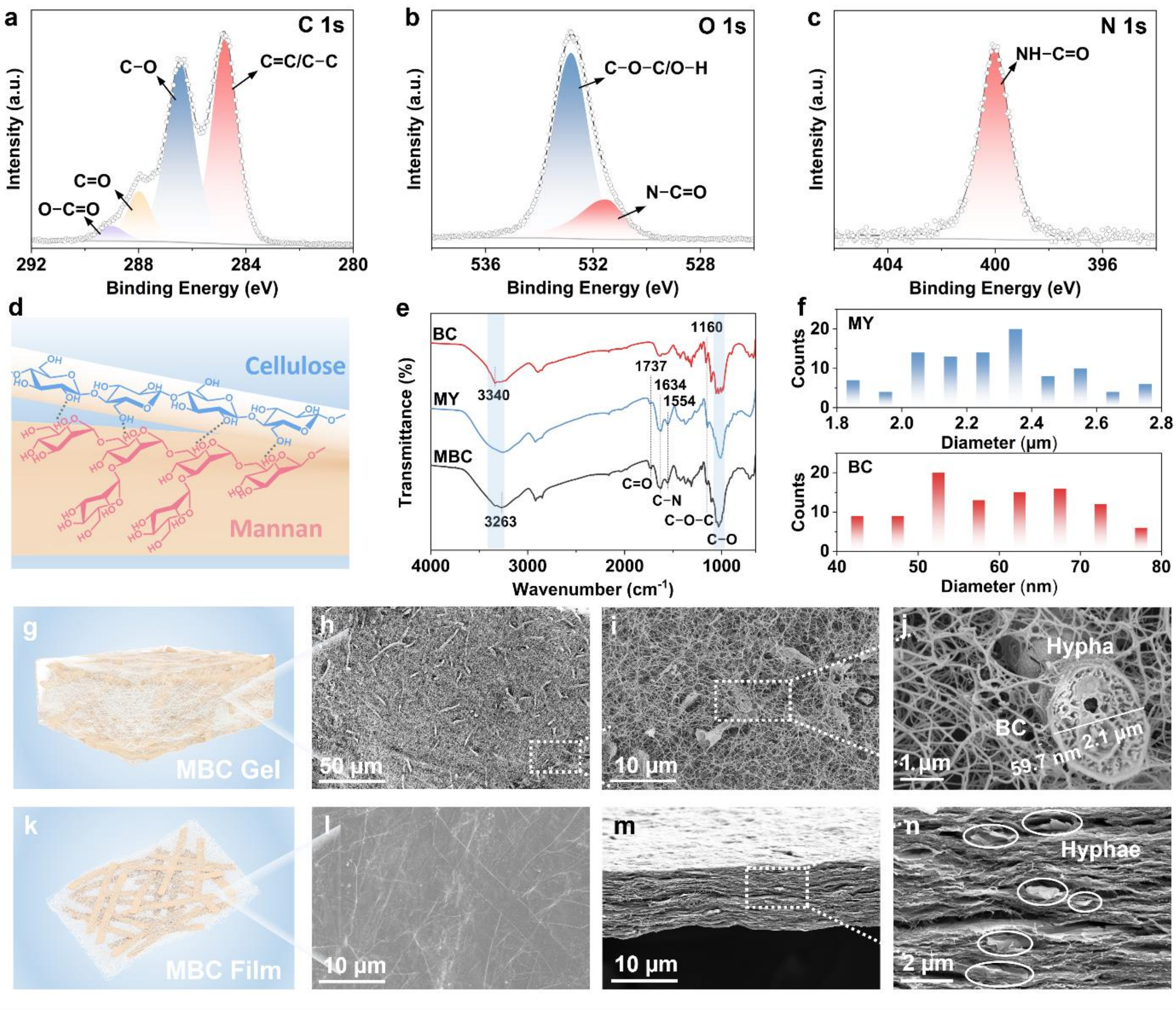

Fig. 2. Structure of MBC hydrogel and MBC film. a-c C 1s, O 1s and N 1s XPS spectra of pure mycelium film. **d** Schematic diagram of hydrogen bonding between mannan on mycelial hyphae and cellulose chains of BC. **e** ATR-FTIR of BC, MY and MBC films. **f** Diameter distribution of MY and BC. **g** Schematic diagram of MBC hydrogel. **h-j** Cross-sectional cryo-SEM images of MBC hydrogel. **k** Schematic diagram of MBC film. **l** Surface SEM images of MBC film. **m, n** Cross-sectional SEM images of MBC films.

Figure S5: *Could the authors add scale bars to these microscope images?*

Response: We have addressed this point as follows. We have added scale bars on Figure S5, which was supplementary Fig. 7 in the revised manuscript.

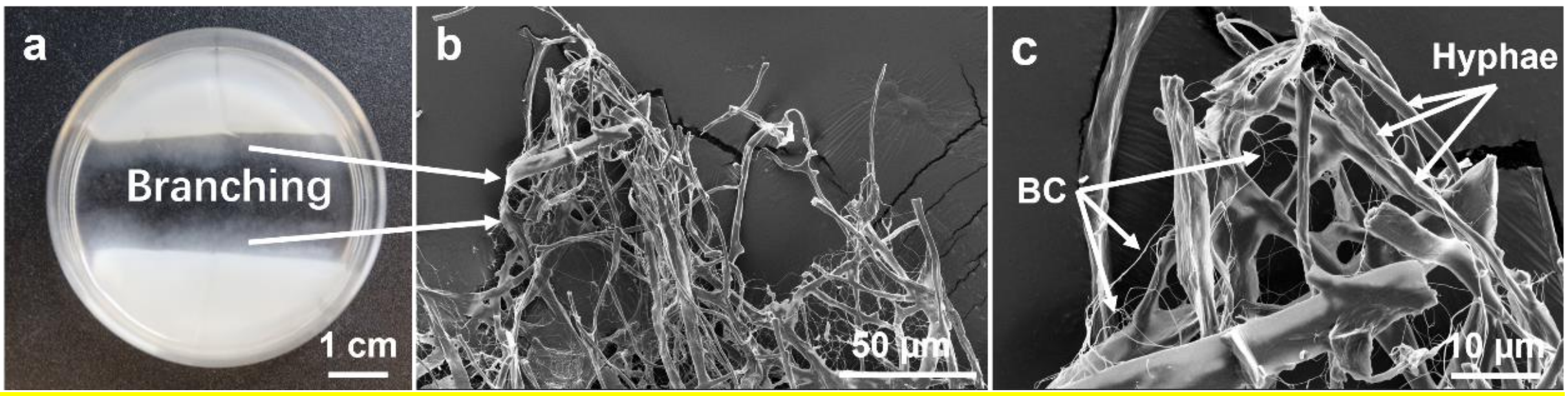

Supplementary Fig. 7. Self-healing and re-growth of MBC hydrogels. a Photographs showing the self-healing and re-growth of MBC hydrogel. **b, c** SEM images of the newly grown thin layer composed of hierarchical BC nanofibers and hyphae.

Line 142: *Although Fig. 3b shows 'self-healing' properties, there is no explanation in the text of how this was achieved. This is important as it demonstrates the mechanism for healing.*

Response: We have addressed this point by addinga detailed explanation of the self-healing mechanism. When the fractured surfaces were brought back into contact, the mycelial hyphae and bacterial cellulose (BC) nanofibers at the interface re-entangled, and hydrogen bonds between their abundant hydroxyl groups were re-established. These interactions enable the **reconstruction of the interfacial network and restore the mechanical integrity of the MBC film**, resulting in the observed self-healing behavior shown in Fig. 3b. We have revised the corresponding discussion in the manuscript, as detailed below.

"When the fractured surfaces were brought back into contact, hyphae and BC re-entangled at interface and hydrogen bonds between them were re-established. These interactions enable the reconstruction of the interfacial network and restore the mechanical integrity of the MBC film, resulting in the observed self-healing behavior." (Page 9, Line 162)

Line 144: *References 29-30 are inappropriate, as the mechanisms in those papers are very different from what is presented here.*

Response: We agree that the previously cited references employed mechanisms not directly comparable to those presented here. We have therefore replaced them with more appropriate references that better align with the biological and structural context of this work. **The newly cited works (Li et al., 2025; Gantenbein et al., 2023)[10,11] report self-healing behavior in**

mycelial systems that is more consistent with the biological and structural mechanisms discussed here, particularly in terms of hyphal growth, network reconnection, and interface-mediated healing. These revisions improve the accuracy and relevance of the literature context. (Page 9, Line 166)

Line 145: *From the images, it appears that only hyphae are growing. Could the authors explain how they identified new growth of both BC and mycelium? There is an absence of bacterial cells in the image, which are necessary to produce more BC, unlike mycelium which is itself alive.*

Response: We have added magnified SEM images to better distinguish the structures of BC and mycelial hyphae in the revised manuscript (Supplementary Fig. 7). BC nanofibers and mycelial hyphae can be clearly differentiated based on their characteristic morphology and size. The BC nanofibers have an average diameter of 59.5 ± 9.9 nm, which is much smaller than that of the hyphae (2.3 ± 0.2 μm). Therefore, BC and mycelial hyphae can be readily distinguished in the magnified SEM images. In addition, bacterial cells are typically difficult to observe in SEM images of mature BC networks because they are often embedded within or covered by the dense cellulose nanofiber matrix. The freeze-drying and sample preparation processes (soaking, washing) may also cause collapse or removal of bacterial cells, making them less visible in SEM images.

"As indicated in Supplementary Fig. 7, both the microscale hyphae and nanoscale BC in the MBC hydrogel continue to grow and branch after being cut for 24 h, forming a new hierarchical thin layer." (Page 9, Line 168)

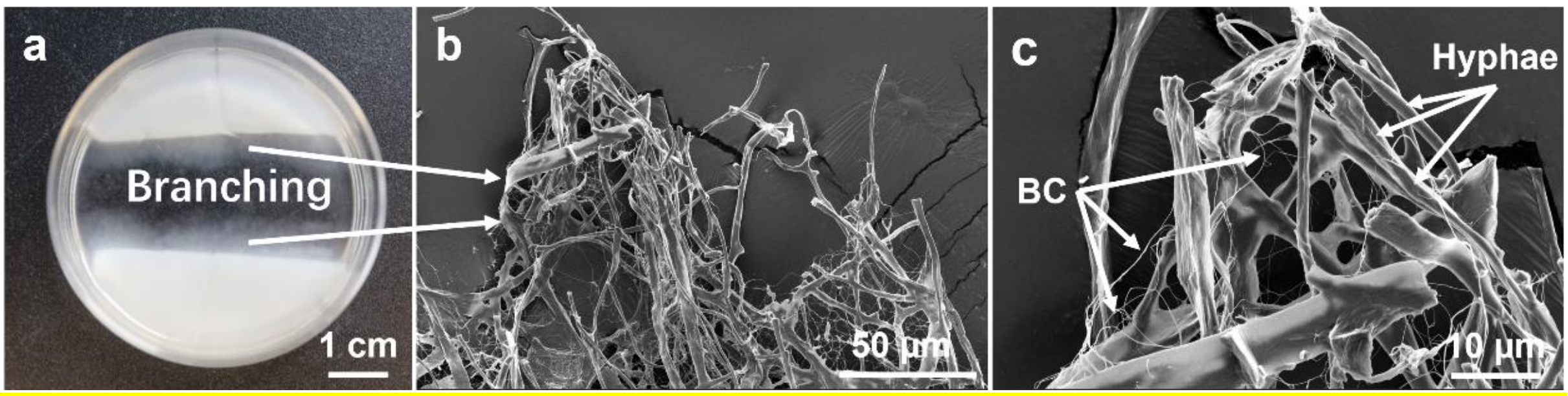

Supplementary Fig. 7. Self-healing and re-growth of MBC hydrogels. a Photographs showing the self-healing and re-growth of MBC hydrogel. **b, c** SEM images of the newly grown

thin layer composed of hierarchical BC nanofibers and hyphae.

Lines 212-227: *Transmittance and haze of films often depend on thickness, fibril density, porosity, and surface roughness. Without investigating these properties, only presenting percentages is weak and unconvincing. Additionally, how repeatable are these data? In the literature, unmodified BC usually shows much lower transmittance than what the authors present here. Could you discuss this discrepancy?*

Response: We have included additional structural analysis that may influence optical properties, including film thickness, surface roughness and porosity analysis in the revised manuscript. The increased haze value and slightly decreased transmittance of MBC films are primarily governed by the increased thickness and the presence of macropores, while the contribution from surface roughness is negligible due to its subwavelength scale.

Regarding *"Transmittance and haze of films often depend on thickness, fibril density, porosity, and surface roughness. Without investigating these properties, only presenting percentages is weak and unconvincing."*

(1) MBC films exhibit a relatively smooth surface as shown in Fig. 2l and Fig. R2, which may originate from the collapse of initially tubular hyphae into flattened, ribbon-like filaments upon drying due to the loss of turgor pressure. The surface roughness of the MBC films was characterized by AFM (Supplementary Fig. 17). **The root mean square (RMS) roughness values for films cultured for 4, 6, and 8 days were 37.18, 37.60, and 44.34 nm, respectively.** Although a slight increase is observed, the RMS roughness remains much smaller than the wavelength of visible light, indicating that the surface is optically smooth. According to the optical scattering relation described in Supplementary Note 5, light scattering from surface roughness is governed by the ratio between the RMS roughness (σ) and the wavelength of incident light (λ)[3]. **Since all the roughness values remain substantially below the wavelength range of visible light (400–800 nm), indicating that the surface roughness of the MBC film has a negligible effect on its haze and transmittance properties.**

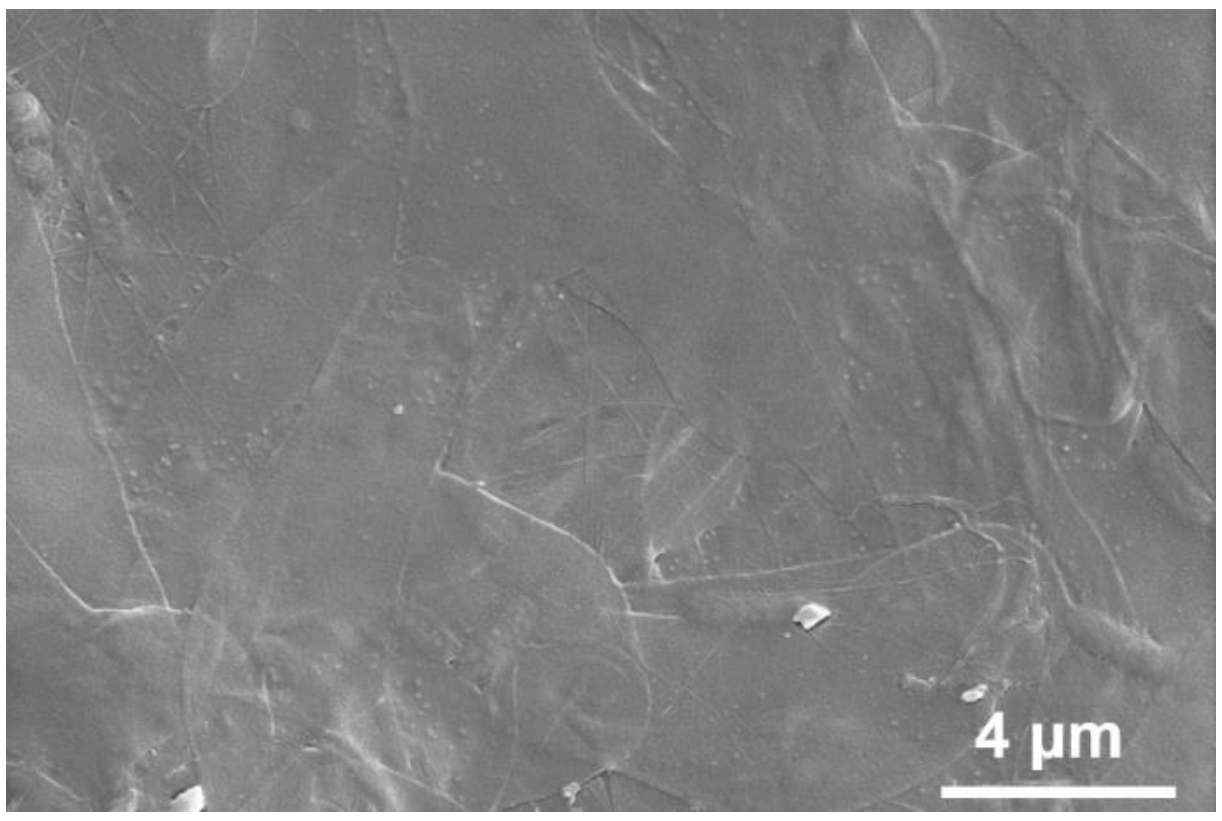

Fig. R2. Surface SEM images of MBC film.

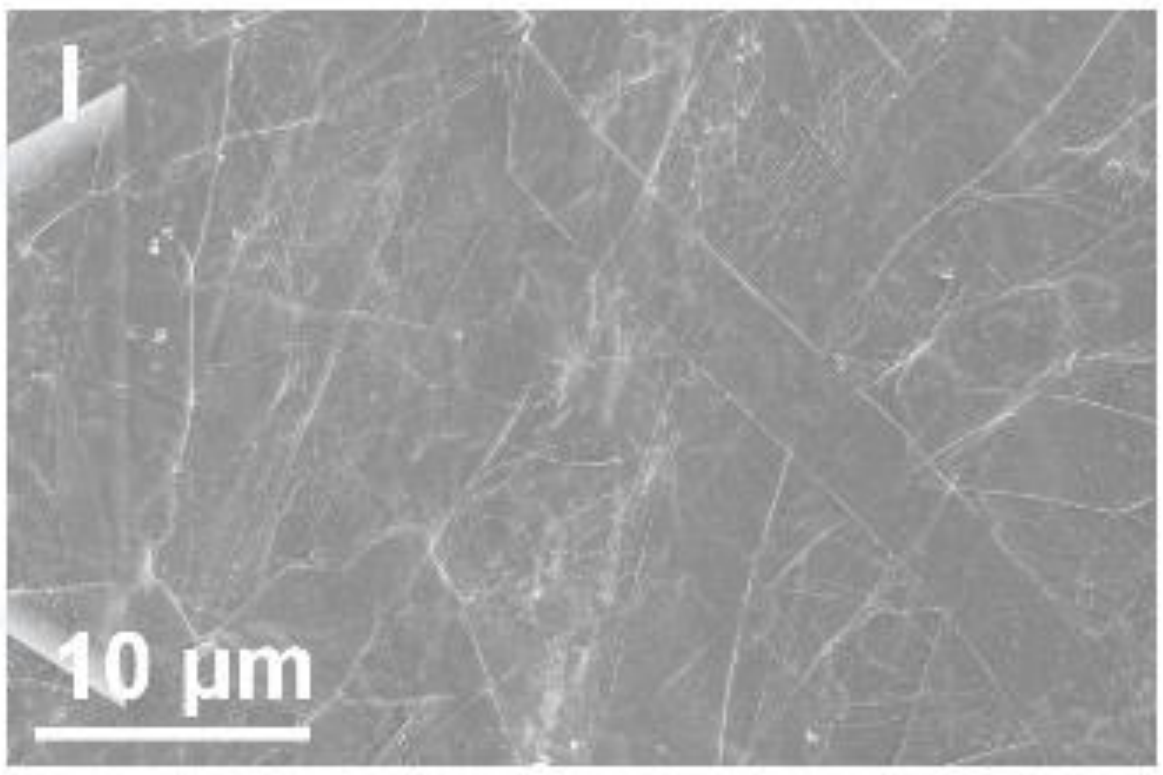

Fig. 2l. Surface SEM images of MBC film.

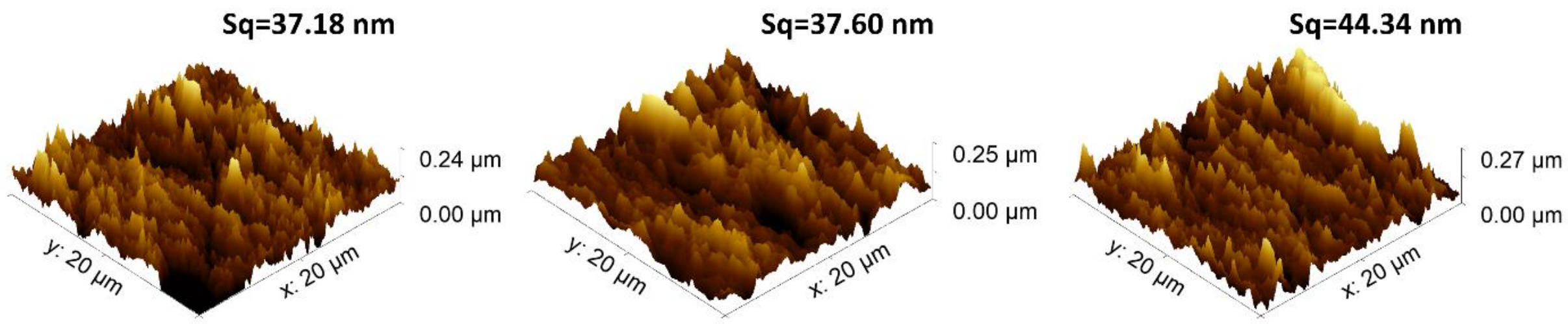

Supplementary Fig. 17. Surface structures of MBC films. Root mean square (RMS) roughness of MBC films. Sq is the RMS roughness.

Supplementary Note 5

$$TIS = 1 - exp\left[-\left(\frac{4\pi\sigma\cos\theta_i}{\lambda}\right)^2\right] \qquad (5)$$

where TIS is the total integrated scattering, θ_i is the incident angle, λ is the laser wavelength, σ is the root mean square (RMS) roughness.

(2) The thicknesses of MBC films with different culture durations are **7.85 ± 0.22 µm (4 days),**

8.96 ± 0.24 μm (6 days) and 9.64 ± 0.44 μm (8 days), respectively. The thickness increases with increasing culture time due to the introduction of more mycelial hyphae and interlamellar pores (Supplementary Fig. 15). This structural evolution enhances light scattering **by increasing the optical path length** within the hierarchical network, which also slightly reduces the optical transmittance.

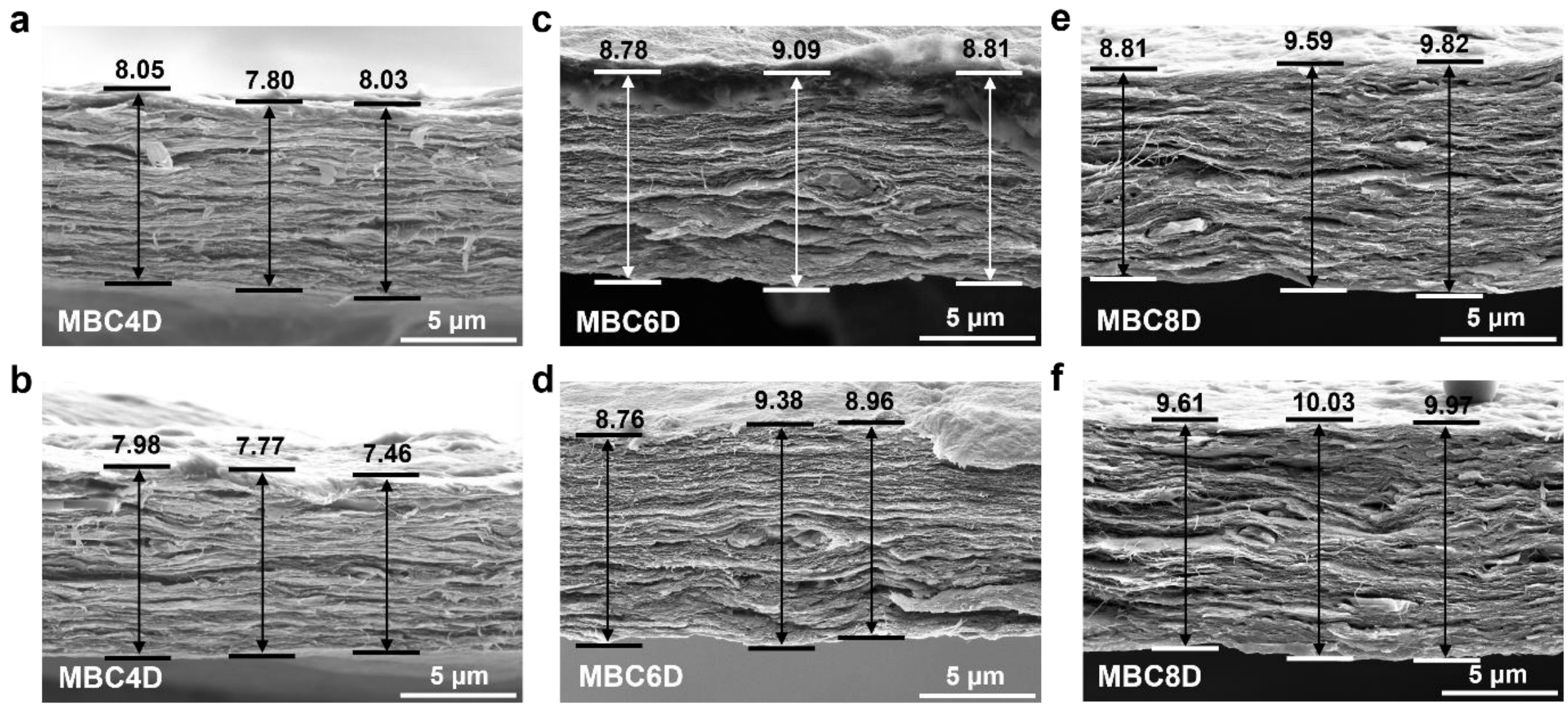

Supplementary Fig. 15. Thicknesses of MBC films. a-f Cross-sectional SEM images of MBC films with different culture durations (4 days, 6 days and 8 days).

(3) Porosity also plays an important role in the haze and transmittance properties of MBC films. However, smaller pores (below ~100 nm), which are difficult to resolve by SEM, are expected to contribute less to light scattering. According to Supplementary Note 2, in the Rayleigh scattering regime ($r \ll \lambda$), the scattering efficiency increases with particle size as $Q_{sca} \propto (r/\lambda)^4$. Pores with diameters much smaller than the wavelength of visible light contribute only weakly to the overall scattering intensity compared to macropores. Therefore, to quantify the pore structure, ImageJ was employed to analyze the pore area fraction in the cross-section. As shown in Supplementary Fig. 16, **the pore area fraction increases from 5.02% to 11.85% with increasing culture time.** The incorporation of more mycelial hyphae leads to the formation of corresponding macropores within the BC matrix. These macropores act as effective scattering centers due to the refractive index (RI) contrast, in agreement with the optical simulation results (Fig. 4j). **The resulting RI mismatch enhances Mie scattering and interfacial reflections at the interfaces among BC nanofibers, hyphae, and air pores, thereby increasing the haze**

value while slightly decreasing the transmittance (Supplementary Notes 1 and 3).

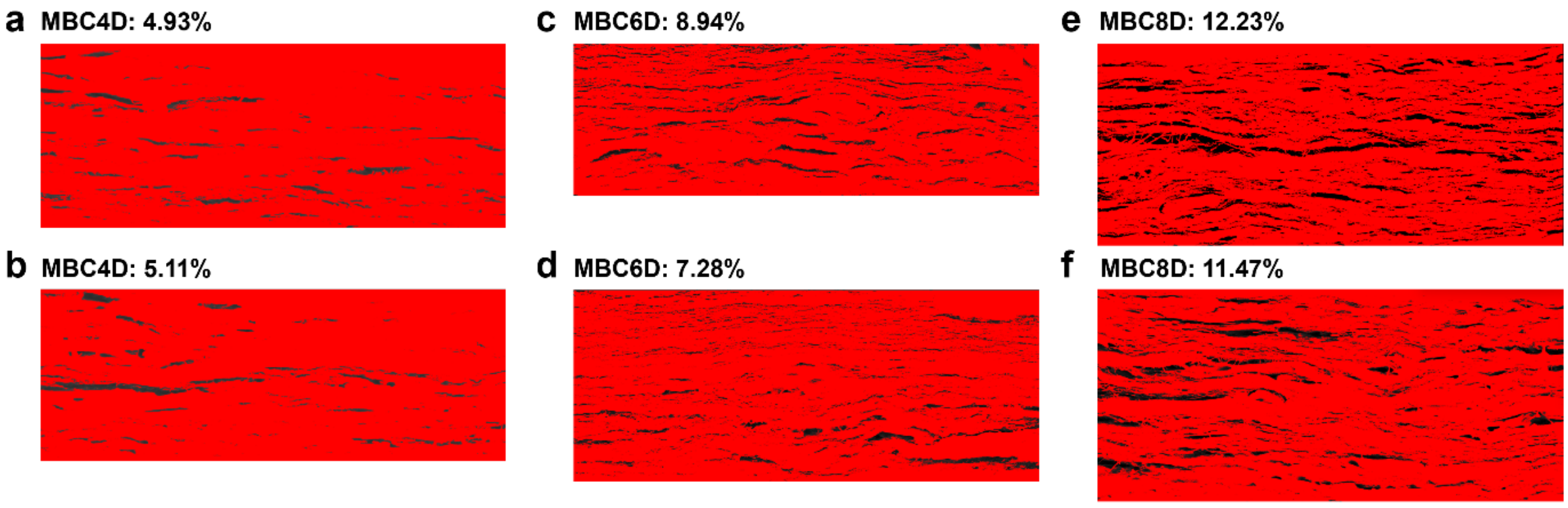

Supplementary Fig. 16. Porous structure of MBC films. Cross-sectional porosity analysis of MBC films with different culture durations using ImageJ.

Regarding *"how repeatable are these data?"*

All optical measurements were repeated **3 times**, and the results showed good reproducibility.

Regarding *"In the literature, unmodified BC usually shows much lower transmittance than what the authors present here. Could you discuss this discrepancy?"*

Regarding the comparison with literature values, most unmodified BC films typically exhibit transmittance in the range of 80–90%[4,12,13], which is consistent with the transmittance values reported in this work. **Some BC films reported in the literature show lower transmittance, which may be due to differences in film thickness.**

Line 283: *Adjusting culture duration will undoubtedly impact film thickness, and film thickness has a direct and strong impact on scattering intensity. Attributing scattering intensity changes only to hyphae and interlamellar pores provides only a partial explanation. The authors should consider all other elements that changed and their impacts.*

Response: We thank the reviewer for this comment. We have added the required experiments and discussions regarding film thickness, porosity, and surface roughness to provide a more thorough analysis of the optical properties. UV-vis measurements showed that the haze of MBC films at 550 nm increased from 16.0% (4 days) to 54.9% (6 days) and 78.1% (8 days). The thicknesses of the MBC films cultured for different durations are 7.85 ± 0.22 μm (4 days), 9.10

± 0.28 μm (6 days) and 9.64 ± 0.44 μm (8 days), respectively. The film thickness gradually increases with culture time, which can be attributed to the increase of hyphal content and the formation of more interlamellar pores. We have added the discussion in the manuscript as detailed below.

"More hyphae and interlamellar air pores are introduced into the MBC films with increasing culture duration, leading to an increased number of interfaces with large refractive index (RI) contrast as well as greater film thickness. The increased film thickness further promotes light scattering by extending the optical path length within the hierarchical network. Moreover, the resulting RI mismatch enhances Mie scattering and interfacial reflections at the interfaces, thereby increasing the haze while slightly decreasing the transmittance (Supplementary Notes 1 and 3)." (Page 14, Line 314)

Figure 5g: *Could the authors explain why the thermocouple was positioned next to the heater rather than on the glass surface?*

Response: The thermocouple was positioned next to the heater to directly monitor the temperature of the simulated heat source beneath the MBC glass. If the thermocouple were placed on the glass surface, the tip of thermocouple would absorb incident light, thereby increasing the measured temperature.

In addition, thermal tape was used between the thermocouple and the heater to improve thermal contact and minimize the influence of air gaps on heat transfer. This configuration ensures a more accurate measurement of the heater temperature and enables a reliable evaluation of the radiative cooling performance of the MBC glass.

Line 347: *Can the authors explain succinctly how they measured the increase in mycelium (and how this was attributed to mycelium rather than BC)? Please provide a percentage increase.*

Response: We thank the reviewer for this valuable comment. A precise quantitative determination of mycelium content is not straightforward; however, the trend can be reliably estimated from TGA curves and SEM observations, both of which consistently indicate an

increase in mycelial content with culture duration.

First, the increase in mycelium content can be inferred from the thermal degradation behavior of the MBC films. As shown in the TGA curves of MBC films cultured for different durations, the total weight loss decreased from 73.8% to 64.9%, while the residual char yield increased correspondingly from 26.2% to 35.1%, representing an increase of approximately 34% in char yield. The higher residual mass of mycelium compared with BC in the TGA curves can be attributed to the complex biochemical composition of fungal biomass. According to the previous studies, fungal cell walls contain polysaccharides, chitin, proteins, and other nitrogen-containing components, which tend to promote char formation during thermal decomposition[14-16]. Therefore, as the culture duration prolonged, the increased hyphal content contributes to higher char residue observed in the TGA results.

Moreover, the increase of hyphal content can also be observed from the cross-section of the films. As shown in Supplementary Fig. 15, MBC films with 4-day and 6-day culture durations exhibit only a few scattered hyphae, while the MBC8D sample shows relatively higher hyphal content.

Line 357: *Please use italics for microorganism names.*

Response: We have corrected this throughout the manuscript. The names of microorganisms have been revised to italics throughout the manuscript.

"The white rot fungi (*Phanerochaete chrysosporium*) were purchased from Outgrow on Etsy." (Page 19, Line 395)

Line 357: *Please provide the name of the seller on Etsy.*

Response: We agree and have added this information. The seller's name (Outgrow) has been added to the Materials section, as detailed below.

"The white rot fungi (*Phanerochaete chrysosporium*) were purchased from Outgrow on Etsy." (Page 19, Line 395)

Line 358: *Please use italics for microorganism names.*

Response: This has been corrected throughout the manuscript. The name of microorganism has been revised as detailed below.

"*Komagataeibacter xylinus* (*K. xylinus*, ATCC 23767) for bacterial cellulose production was purchased from ATCC." (Page 19, Line 396)

Line 367: *Please use italics for microorganism names.*

Response: We agree and have made this correction. The name of microorganism has been revised as detailed below.

"The bacterial strain (*K. xylinus*) was cultured and maintained in a liquid culture containing 5 g L^{-1} yeast extract, 3 g L^{-1} peptone, and 25 g L^{-1} mannitol." (Page 19, Line 404)

Line 367: *Did your media contain any glucose? If not, can the authors explain how they achieved any BC production?*

Response: Glucose was indeed present in the culture medium and served as the primary carbon source for BC production. We apologize that this information was omitted in the original manuscript. The composition of the culture medium has now been clarified in the revised manuscript.

"Besides, the liquid medium for MY hydrogel (LM medium) was prepared by dissolving 10 g malt extract, 5 g yeast extract in 1000 g deionized water. The liquid medium for BC hydrogel (HS medium) was prepared by dissolving 10 g glucose, 2.5 g yeast extract, 2.5 g peptone, 2.7 g disodium phosphate anhydrous and 1.5 g citric acid monohydrate in 1000 g deionized water. The co-culture medium (LM-HS) is the combination of LM and HS medium with a volume ratio of 7:3." (Page 19, Line 405)

Line 368: *Please provide details on how the fungi and bacteria were prepared for co-culture and how much of each was added.*

Response: We appreciate this comment and have added the requested details. Details of the preparation of the fungi and bacteria for co-culture have been added in the revised manuscript, as shown below.

"First, the fungal spores purchased from Etsy were cultured and maintained in a liquid culture containing 8 g L^{-1} glucose and 1.5 g L^{-1} peptone under sterile conditions. The bacterial strain (*K. xylinus*) was cultured and maintained in a liquid culture containing 5 g L^{-1} yeast extract, 3 g L^{-1} peptone, and 25 g L^{-1} mannitol. Besides, the liquid medium for MY hydrogel (LM medium) was prepared by dissolving 10 g malt extract, 5 g yeast extract in 1000 g deionized water. The liquid medium for BC hydrogel (HS medium) was prepared by dissolving 10 g glucose, 2.5 g yeast extract, 2.5 g peptone, 2.7 g disodium phosphate anhydrous and 1.5 g citric acid monohydrate in 1000 g deionized water. The co-culture medium (LM-HS) is the combination of LM and HS medium with a volume ratio of 7:3. All the liquid medium was sterilized by autoclaving at 121 °C for 15 min. For the co-culture of mycelium and BC, approximately 3 mL of fungal spore suspension and 3 mL of cellulose-producing bacterial culture were added into 10 mL of LM-HS medium in a petri dish (diameter: 5 cm; height: 1 cm), followed by static incubation for 4–10 days to allow hydrogel formation. All procedures were conducted under sterile conditions. All cultures were incubated under room temperature (~23–25 °C) for desired culture duration (4 days, 6 days and 8 days)." (Page 19, Line 402)

Line 370: *Could you please state the temperature range for "room temperature" in your geographical location?*

Response: In this study, "room temperature" refers to approximately 23–25 °C; this has been explicitly stated in the revised manuscript.

"All procedures were conducted under sterile conditions. All cultures were incubated under room temperature (~23–25 °C)" (Page 19, Line 415)

Line 371: *Were all films treated with NaOH? If so, can the authors explain how any BC could grow during breakage and self-healing without any living cells to produce it? And how did the*

mycelium survive this treatment?

Response: The MBC hydrogel samples used for the self-healing experiments were not treated with NaOH; consequently, the living microbial cells remained active within the hydrogel network. During the self-healing process, the mycelium hyphae continued to grow and the bacterial cells continued to produce new BC, enabling structural regrowth and network reconnection.

Line 373: *Can the authors please provide details on the air-drying procedure, i.e., how long were samples air-dried, and how was the endpoint determined?*

Response: The air-drying process was carried out at room temperature (~23–25 °C) for approximately 12 h (overnight). The drying endpoint was determined by the complete disappearance of visible surface moisture and the formation of a stable, self-supporting film. This information has now been added to the Methods section.

"Finally, the hydrogels were air-dried at room temperature with a relative humidity of approximately 30% for 12 hours to obtain films." (Page 19, Line 419)

Line 394: *Please reread this sentence for clarity.*

Response: We appreciate all the helpful suggestions. We have revised this sentence for clarity as detailed below.

"The tensile properties of the films were measured using a tensile tester (ADMET, USA). The films were cut into strips with a width of ~4 mm and conditioned at 30% relative humidity for 24 h at room temperature prior to testing." (Page 20, Line 446)

Reviewer #3: *I co-reviewed this manuscript with one of the reviewers who provided the listed reports. This is part of the Nature Communications initiative to facilitate training in peer review and to provide appropriate recognition for Early Career Researchers who co-review manuscripts.*

Response: We sincerely thank Reviewer #3 for co-reviewing this manuscript. We greatly appreciate the time and effort devoted to the evaluation of our work. We have carefully considered all comments provided during the review process and have made the corresponding revisions to strengthen the manuscript.

References

1. Y. Zhang, Q. Liu, J. Li, H. Yao, Y. Hou, J. Hou, "Large-scale fabrication of flexible antiglare film with superhydrophobic surface: Toward high transmittance and high haze," *Chemical Engineering Journal*, *511* (**2025**), 161805. https://doi.org/10.1016/j.cej.2025.161805
2. N.A.R. Gow, M.D. Lenardon, "Architecture of the dynamic fungal cell wall," *Nature Reviews Microbiology*, *21* (**2023**), 248-259. http://doi.org/10.1038/s41579-022-00796-9
3. J. Harvey, S. Schröder, N. Choi, A. Duparré, "Total integrated scatter from surfaces with arbitrary roughness, correlation widths, and incident angles," *Optical Engineering*, *51* (**2012**), 3402. http://doi.org/10.1117/1.OE.51.1.013402
4. S. Wang, T. Li, C. Chen, W. Kong, S. Zhu, J. Dai, et al., "Transparent, Anisotropic Biofilm with Aligned Bacterial Cellulose Nanofibers," *Advanced Functional Materials*, *28* (**2018**), 1707491. http://doi.org/10.1002/adfm.201707491
5. W.-B. Sun, Z.-M. Han, X. Yue, H.-Y. Zhang, K.-P. Yang, Z.-X. Liu, et al., "Nacre-Inspired Bacterial Cellulose/Mica Nanopaper with Excellent Mechanical and Electrical Insulating Properties by Biosynthesis," *Advanced Materials*, *35* (**2023**), 2300241. http://doi.org/10.1002/adma.202300241
6. K. Yu, S.T. Chua, R. Zhao, A. Smith, M. Kühl, A.G. Smith, et al., "Artificial Symbiosis for Bulk Production of Bacterial Cellulose Composites," *Advanced Materials*, *38* (**2026**), e14125. http://doi.org/10.1002/adma.202514125
7. T. Wang, J. Zeng, F. Hu, H. Lou, G. Liu, R. Lu, et al., "Ultrastrong Aligned and Bridged Bacterial Cellulose-Supported Liquid Metal/Silver Nanowire Films for Superior Electromagnetic Interference Shielding and Thermal Management," *Advanced Functional Materials*, *n/a* (**2026**), e74439. http://doi.org/10.1002/adfm.74439
8. H.-L. Gao, S.-M. Chen, L.-B. Mao, Z.-Q. Song, H.-B. Yao, H. Cölfen, et al., "Mass production of bulk artificial nacre with excellent mechanical properties," *Nature Communications*, *8* (**2017**), 287. http://doi.org/10.1038/s41467-017-00392-z
9. A. Agrawal, T. Ipsen, B.T.M. Dentinger, S.E. Naleway, "Multilayer, Functionally Graded Organic Living Hydrogels Built by Pure Mycelium," *JOM*, *77* (**2025**), 9241-9252. http://doi.org/10.1007/s11837-025-07685-5
10. Z. Li, X. Deng, G. Chen, Z. Zhang, J. Han, X. Lu, "Mycelium-derived self-healing electrodes for high-performance and biodegradable supercapacitors," *Chemical Engineering Journal*, *524* (**2025**), 169372. http://doi.org/10.1016/j.cej.2025.169372
11. S. Gantenbein, E. Colucci, J. Käch, E. Trachsel, F.B. Coulter, P.A. Rühs, et al., "Three-dimensional printing of mycelium hydrogels into living complex materials," *Nature Materials*, *22* (**2023**), 128-134. http://doi.org/10.1038/s41563-022-01429-5
12. P. Lv, H. Zhou, M. Zhao, D. Li, K. Lu, D. Wang, et al., "Highly flexible, transparent, and conductive silver nanowire-attached bacterial cellulose conductors," *Cellulose*, *25* (**2018**), 3189-3196. http://doi.org/10.1007/s10570-018-1773-8
13. F. Guan, S. Chen, J. Yao, W. Zheng, H. Wang, "ZnS/Bacterial Cellulose/Epoxy Resin (ZnS/BC/E56) Nanocomposites with Good Transparency and Flexibility," *Journal of Materials Science & Technology*, *32* (**2016**), 153-157. http://doi.org/10.1016/j.jmst.2015.08.014
14. M. Jones, T. Bhat, E. Kandare, A. Thomas, P. Joseph, C. Dekiwadia, et al., "Thermal

Degradation and Fire Properties of Fungal Mycelium and Mycelium - Biomass Composite Materials," *Scientific Reports*, *8 (***2018)**, 17583. http://doi.org/10.1038/s41598-018-36032-9

15. N. Chulikavit, T. Huynh, C. Dekiwadia, A. Khatibi, A. Mouritz, E. Kandare, "Influence of growth rates, microstructural properties and biochemical composition on the thermal stability of mycelia fungi," *Scientific Reports*, *12 (***2022)**, 15105. http://doi.org/10.1038/s41598-022-19458-0
16. N. Chulikavit, T. Huynh, C. Wang, A.C.Y. Yuen, A. Khatibi, A. Mouritz, et al., "Engineering mycelium fungi into an effective char-forming thermal protection material via alkaline deacetylation," *Polymer Degradation and Stability*, *212 (***2023)**, 110355. http://doi.org/10.1016/j.polymdegradstab.2023.110355

Reviewer #1 (Remarks to the Author):

The authors have addressed all my concerns.

Response: We thank Reviewer #1 for the positive evaluation of our revised manuscript and for confirming that the concerns raised in the previous round of review have been fully addressed.

Reviewer #2 (Remarks to the Author):

The authors have made a good effort to address the initial comments and the further tests and images provided in the revised manuscript are very welcome. There are still some areas that require more detail in both methods and results, and some conclusions which are not supported by the evidence provided.

Response: We thank the reviewer for the positive assessment of our revised manuscript and for recognizing the value of the additional experiments and analyses. We agree that several aspects of the Methods and Results required further clarification, and that some conclusions needed to be aligned more carefully with the available evidence. Accordingly, we have substantially revised the manuscript by adding detailed descriptions of the pre-culture conditions, inoculation procedures, self-healing experiments, and medium compositions. We have also added an EDS analysis to complement the TGA results, revised the interpretation of the thickness and porosity measurements, and explicitly stated the limitations of these analyses. In addition, several conclusions have been revised to better reflect the supporting evidence. We believe these changes have significantly improved the rigor, clarity, and reproducibility of the manuscript, and we address each specific point below.

Major concerns:

Comment 1: *Authors claim that their MBC hydrogel is distinct from previously reported mycelium hydrogels due to the absence of an extensive hyphal network, but they do not provide*

a mechanistic explanation for this observation. Given that hyphal network formation is a well-known characteristic of fungal growth, the authors should discuss in depth why the co-culture conditions suppress hyphal network development. This explanation is critical to substantiate their claim of a unique co-culture system and would significantly strengthen the process–structure–property relationship that is central to this work.

Response: We thank the reviewer for this important comment. To address it precisely, we would first like to clarify our understanding of the term "extensive hyphal network." We interpret it as referring to the dense, white aerial hyphal layer that forms above the liquid surface in previously reported mycelium hydrogels, as distinct from the submerged hyphae that grow within the liquid medium. Under this interpretation, the relevant observation is the limited formation of aerial hyphae in our system, for which we provide a mechanistic explanation below. We also note that the central novelty of this work lies in the successful co-culture of mycelium and bacterial cellulose (BC) and in exploiting the resulting hierarchical structure for tunable optical properties, rather than in the absence of an aerial hyphal layer.

Mechanistic explanation for the limited aerial hyphal growth

Mechanistically, aerial hyphae develop when hyphae at the air–liquid interface escape the liquid and extend into the air—a process promoted by the secretion of hydrophobic cell-wall proteins (hydrophobins) and strongly dependent on both the fungal species and the culture mode. In submerged liquid culture, many fungi grow predominantly as submerged hyphae or pellets and produce few aerial hyphae. The strain used here, *Phanerochaete chrysosporium* (PC), intrinsically shows this behavior: under our static, submerged, nutrient-rich liquid culture, it forms mainly submerged hyphae and does not develop the dense white aerial hyphal layer characteristic of previously reported mycelium hydrogels within the culture window used. We therefore emphasize that the limited aerial hyphal growth originates from the intrinsic growth behavior of the selected strain under submerged liquid culture, and is not a result of suppression by BC.

Strain screening and the choice of PC

To support this, we performed a strain-screening experiment. Abundant aerial hyphae are

typically highly porous and substantially increase surface roughness, which strongly enhances light scattering and blocking and causes the resulting films to appear opaque or white with limited optical tunability. We evaluated five fungal species grown in liquid medium under similar conditions, and found that most developed extensive aerial hyphal networks after 8 days (Supplementary Fig. 1). In contrast, PC exhibited relatively compact growth with limited aerial hyphal formation, making it the most suitable strain for preparing transparent composite films with tunable optical properties.

Medium optimization for the co-culture

Based on this strain selection, we further optimized the culture medium to support the simultaneous growth of BC and PC in liquid culture, yielding mixed hydrogels. Upon drying, these hydrogels were converted into hierarchical composite films whose structure and optical properties could be tuned by controlling the growth duration.

We have revised the manuscript as detailed below.

Through strain screening, we found that *Phanerochaete chrysosporium* (PC) forms only submerged hyphae in liquid medium without developing a white aerial hyphal layer within the desired culture duration (Supplementary Fig. 1). This growth behavior differs from previously reported bilayer mycelium hydrogels and provides a suitable structure for co-culturing with BC and regulating the optical properties of the resulting films[26]. (Page 4, Line 61)

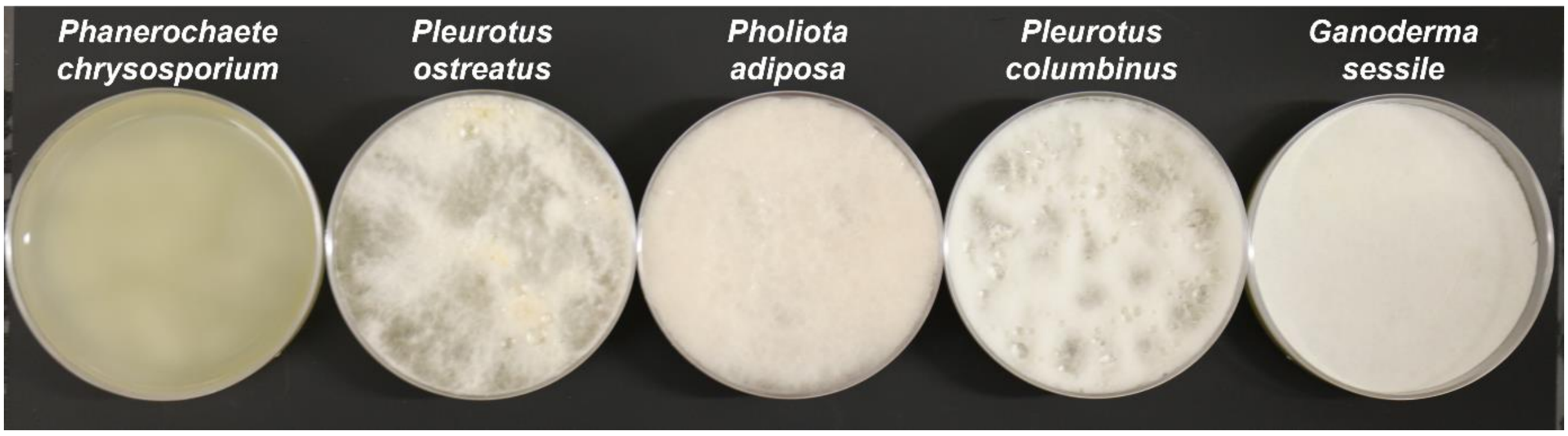

Supplementary Fig. 1. Fungal species screening. Photographs of 5 species grown in LM medium (liquid medium for mycelium hydrogel) under room temperature for 8 days.

We have also added details in the Materials section as shown below.

Pleurotus ostreatus, *Pholiota adiposa* and *Pleurotus columbinus* were purchased from mymushshop on Etsy. *Ganoderma sessile* was purchased from Primefungi on Etsy. (Page 20, Line 403)

Comment 2: *Additionally, P. chrysosporium is known to be a very efficient degrader of cellulose. The authors should address why this fungus has been chosen and why it did not metabolise the bacterial cellulose in this co-culture system, and provide a mechanistic discussion of how the BC matrix was preserved despite the presence of a known cellulolytic organism. This is a fundamental question that has not been adequately addressed in the current revision.*

Response: We thank the reviewer for raising this important point, and we address both why *P. chrysosporium* (PC) was chosen and, mechanistically, why the BC matrix was preserved despite the presence of this cellulolytic fungus. PC was selected because, among the strains we screened, it forms only limited aerial hyphae in submerged liquid culture (see our response to Comment 1), which is essential for obtaining transparent, optically tunable films, and because it is a well-characterized model white-rot fungus. Mechanistically, the preservation of the highly crystalline BC matrix in our short-term, nutrient-rich co-culture is governed by two main factors, described below.

Mechanism 1: carbon catabolite repression in the nutrient-rich medium

The co-culture was carried out in a nutrient-rich liquid medium designed to support simultaneous microbial growth, rather than under the nutrient-limited, wood-decaying conditions that normally trigger aggressive lignocellulose degradation. The medium contains readily metabolizable carbon sources (glucose and malt extract), and the expression of cellulase-related genes in white-rot fungi, including PC, is strongly repressed in the presence of such carbon sources through carbon catabolite repression[1-3]. Consequently, PC preferentially consumes the soluble nutrients and functions primarily as a living structural component that forms an interpenetrating network with BC, rather than secreting the cellulases required to depolymerize the BC nanofibers.

Mechanism 2: high crystallinity of the BC network

Even if limited cellulolytic activity occurs, the BC network is intrinsically resistant to rapid

enzymatic degradation. BC possesses a highly crystalline nanofibrous structure, with reported crystallinity values typically in the range of 60–90%[4]. White-rot fungi preferentially attack amorphous cellulose regions during the early stages of degradation[5], so the dense, highly crystalline BC network is degraded only slowly; in some cases, the overall crystallinity even increases after 4–6 weeks owing to the preferential removal of amorphous domains[6]. Over the short cultivation period used here (8 days), this kinetic resistance allows the BC matrix to remain largely intact.

Summary

Taken together, the nutrient-rich medium (which suppresses cellulase expression) and the high crystallinity of the BC network (which limits enzymatic accessibility), combined with the short culture duration, mechanistically explain why the BC matrix is largely preserved in our co-culture despite the cellulolytic capability of PC.

We have added the related discussion in the revised manuscript as detailed below.

To enable the simultaneous growth of mycelium and BC, we designed a composite LM–HS medium that provides the nutrients required for both mycelial growth and BC biosynthesis. The presence of readily metabolizable glucose is known to repress the cellulase-related gene expression in white-rot fungi, including PC[29,30], while the high crystallinity of BC further limits its enzymatic degradation[31,32], allowing the BC network to remain largely preserved during the co-culture process. (Page 6, Line 96)

Comment 3: *The authors' response regarding the inoculation ratio remains unsatisfactory. Authors sated that a fixed spore-to-bacteria numerical ratio cannot be precisely defined due to the dynamic nature of the cultures, simply reporting a volumetric ratio (3 mL spores : 3 mL bacteria: 10 mL medium) is insufficient to ensure reproducibility and rigorous interpretation of the results. In standard microbiology practice, inocula are routinely standardised using quantitative measures such as OD600, CFU/mL, or spore counts per mL at the time of inoculation, regardless of the dynamic nature of the cultures. Without such standardisation, it*

is unclear whether the reported material properties are reproducible across different batches and different lab conditions. The actual ratio of fungal to bacterial biomass introduced into the co-culture system could vary significantly between experiments. The authors should provide at least one quantitative measure of the starting biomass concentration for both the fungal spore suspension and the bacterial culture, and discuss how batch-to-batch variation in inoculum concentration may affect the resulting material properties.

Response: We thank the reviewer for this helpful comment, and we agree that quantitative characterization of the starting inocula is important for reproducibility. We address the reviewer's two specific requests in turn below.

Regarding *"provide at least one quantitative measure of the starting biomass concentration for both the fungal spore suspension and the bacterial culture":*

We measured the optical density (OD600) of 4 batches of bacterial and fungal pre-cultures that successfully supported the co-growth of mycelium and BC. The OD600 was 0.018 ± 0.001 for the liquid culture collected from beneath the BC pellicle and 0.023 ± 0.006 for the fungal liquid culture. These quantitative values have been added to the revised manuscript, as shown below.

The OD600 value of the liquid culture collected from beneath the BC pellicle was 0.018 ± 0.001, while that of the mycelial liquid culture was 0.023 ± 0.006. (Page 20, Line 425)

The OD600 values of four fungal pre-cultures and four bacterial pre-cultures were measured using a UV–vis spectrophotometer (Shimadzu, UV-2600, Japan). (Page 22, Line 459)

Regarding *"discuss how batch-to-batch variation in inoculum concentration may affect the resulting material properties":*

We agree that the OD600 measurements showed fluctuations in inoculum concentration between different batches, particularly for the fungal pre-culture. However, in our protocol, the inoculum volumes, medium composition, and static culture duration were all kept constant. Hydrogel formation occurred only after the two microorganisms had co-grown into a continuous interpenetrating network. In this fixed-duration, growth-saturated regime, moderate

differences in the initial inoculum mainly affect the time required to reach a given network density rather than the final network architecture. Consistent with this, the mechanical and optical properties measured across independently grown samples showed relatively small standard deviations, indicating that the resulting material properties are reproducible despite variation in the starting inoculum concentration.

Comment 4: *The methods section still lacks critical details regarding the pre-culture conditions for both organisms. The authors should specify: the temperature, agitation speed, volume of media, and duration for which each pre-culture was grown or maintained; whether a new pre-culture was prepared for each experiment or whether it was maintained continuously; and whether 3 mL of liquid culture was used directly, or whether mycelium mats or BC pellicles were added instead. If pellicles were used, the authors must explain how this translates to a consistent 3 mL volume across replicates.*

Response: We thank the reviewer for these comments, and we agree that these experimental details are important for the reproducibility and interpretation of the results.

Pre-culture conditions and maintenance

Regarding the temperature, agitation, volume, duration, and maintenance of each pre-culture: the fungal pre-culture was maintained at room temperature (23–25 °C) for 2 weeks, during which it was shaken by hand once per day to maintain a more uniform distribution of the mycelium; no mechanical agitation was applied. The bacterial pre-culture was maintained under static conditions at room temperature for 3 weeks. Both pre-cultures were prepared in sterilized 500 mL bottles, with the culture volume kept at approximately half of the bottle volume. After cultivation, the pre-cultures were stored at 3 °C to maintain relatively stable inoculum concentrations and were used directly for subsequent co-culture experiments; new pre-cultures were not prepared for each experiment.

Form of the inoculum: liquid culture, not mats or pellicles

Regarding whether liquid culture was used directly or whether mats/pellicles were added:

during the fungal pre-culture, no mycelial mat formation was observed; the bacterial pre-culture formed a BC pellicle at the air–liquid interface, and the liquid culture collected from beneath this pellicle was used for inoculation. Neither mycelial mats nor BC pellicles were transferred during the pre-culture or co-culture processes, only the liquid cultures were used. The reported 3 mL therefore always refers to a defined volume of liquid culture, which ensures a consistent inoculum volume across replicates.

For the pre-culture of fungi, 3 mL of the purchased liquid culture was inoculated into a sterilized liquid medium containing 8 g L^{-1} glucose and 1.5 g L^{-1} peptone. The fungal pre-culture was maintained at room temperature (23–25 °C) for 2 weeks during which the culture was shaken by hand once per day to maintain a more uniform distribution of the mycelium.

For the pre-culture of BC, 3 mL of the purchased bacteria strain (*K. xylinus*) was inoculated into a sterilized liquid medium containing 5 g L^{-1} yeast extract, 3 g L^{-1} peptone, and 25 g L^{-1} mannitol. The bacterial pre-culture was maintained under static conditions at room temperature for 3 weeks, during which a BC pellicle formed at the air-liquid interface. For inoculation, the liquid culture collected from beneath the BC pellicle was used.

Both fungal and bacterial pre-cultures were prepared in sterilized 500 ml bottles, with the culture volume maintained at approximately half of the bottle volume. After cultivation, the fungal and bacterial pre-cultures were stored at 3 °C to maintain relatively stable inoculum concentrations and were directly used for subsequent co-culture experiments. No BC pellicles or mycelial mats were directly transferred between experiments. (Page 20, Line 412)

Comment 5: *Authors have added soil burial experiments in response to the biodegradability concern, which is welcomed. But the evidence presented in Supplementary Fig. 5 raises questions about experimental consistency. Specifically, the soil texture and appear noticeably different across the three timepoints (initial, 2 weeks, and 4 weeks), which makes it difficult to confirm that the same samples are being monitored throughout the experiment. In a rigorous soil burial degradation study, it is standard practice to track the same samples continuously*

under identical environmental conditions, with consistent imaging conditions including fixed camera angle, lighting, and distance. The authors need to clarify whether the images presented represent the same experimental batch monitored over time, or whether different soil batches or containers were used at each timepoint. In addition, to strengthen the biodegradability claims, the authors should supplement the visual observations with quantitative measurements such as mass or area loss percentage at each timepoint, which would provide more convincing and reproducible evidence of degradation. The inclusion of scale bars in the images would also improve the clarity and rigor of this figure. The authors should also clarify whether the samples used for biodegradability testing were treated with NaOH. If not, we suspect we should see mycelium growth. If so, this is inconsistent with the self-healing experiments where living cells are required, and this distinction should be made explicit in the manuscript.

Response: We thank the reviewer for this careful and constructive comment, and we apologize for the inconsistency in the original images. We would like to transparently clarify what was done. In the original test, the same MBC film sample was monitored throughout; however, the surrounding soil was changed partway through because the initial soil batch contained large aggregates that prevented uniform contact with the film and complicated consistent imaging. Replacing the soil altered the background appearance of the photographs, resulting in the texture differences noted by the reviewer. However, it did not affect the degradation of the film itself. To remove any ambiguity and to provide more rigorous, quantitative evidence, we have repeated the experiment under controlled conditions and now report a quantitative area-loss measurement, as described below.

Repeated soil-burial experiment with consistent conditions

In the repeated test, a single MBC film was buried at a depth of approximately 2 cm in soil within an aluminum box; the soil was not replaced at any point during the 4-week period. The setup was kept in the laboratory at room temperature (23–25 °C) and a relative humidity of 30%, and 50 mL of water was added every 4 days to maintain moisture. The film used in this test was obtained by air-drying a NaOH-treated MBC hydrogel, and its area was recorded before and after burial. Any residual differences in soil appearance between photographs therefore arise only from surface disturbance during burial and from the periodic watering, not from

changing the soil. The Methods section has been updated accordingly, as shown below.

Biodegradability test

The biodegradability of the MBC film (2 cm × 2 cm) was evaluated by burying the film at a depth of approximately 2 cm in soil contained within an aluminum box for 4 weeks. The setup was placed in the laboratory at room temperature (23–25 °C) and a relative humidity of 30%. To maintain soil moisture, 50 mL of water was added to the soil every 4 days throughout the experiment. The soil was not replaced during the 4-week burial period. The MBC film used in this experiment was obtained by air-drying a NaOH-treated MBC hydrogel. The area of the film was recorded before and after burial for comparison. (Page 22, Line 476)

Quantitative degradation results

As shown in Supplementary Fig. 6, the initially intact MBC film progressively degraded and fragmented into small pieces after 4 weeks. Quantitatively, the film area decreased from an initial ~4 cm^2 to less than 1 cm^2, corresponding to an area loss of approximately 75%. This quantitative measurement provides objective, reproducible evidence of degradation that does not rely on visual comparison of the soil background. We have revised the manuscript as shown below.

The biodegradability of the MBC film was also evaluated through a soil burial test. As shown in Supplementary Fig. 6, the MBC film degrades into small fragments after 4 weeks of soil burial, accompanied by an approximately 75% area loss, indicating its good biodegradability. (Page 8, Line 147)

Distinction between the biodegradation and self-healing samples

Finally, we clarify the point regarding NaOH treatment. The samples used in the biodegradation test were intentionally NaOH-treated to remove residual cells and nutrients, which is why no mycelial regrowth was observed during burial. This preparation is deliberately different from that of the self-healing experiments, in which the hydrogels were not NaOH-treated and therefore retained living cells capable of continued growth. The two experiments thus use distinct sample states: non-living, purified films for biodegradation and living hydrogels for self-healing, and we have now made this distinction explicit in the manuscript.

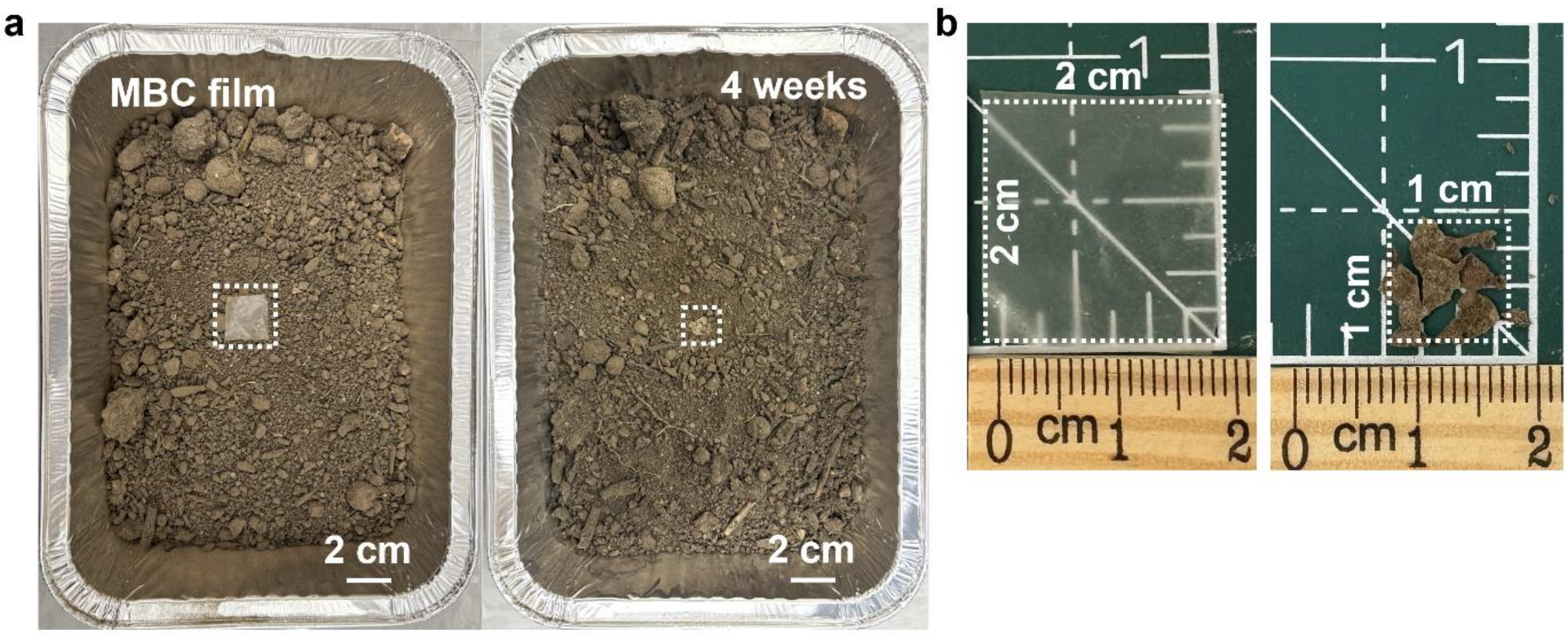

Supplementary Fig. 6. Biodegradability of the MBC film. a Photographs of the MBC film before and after 4 weeks of soil burial. **b** Photographs showing the area of the MBC film before and after 4 weeks of soil burial.

Minor concerns:

Comment 1: *The authors should explain why the air-dried samples on glass plates (Fig 3d and Fig S8) appear considerably more transparent than those dried without glass (Fig 5). It is unclear whether this difference is due to film thickness, surface contact effects during drying, or other factors. This discrepancy should be addressed explicitly.*

Response: We thank the reviewer for this comment. We would like to clarify that the apparent transparency difference between the MBC glass (Fig. 5a) and the free-standing MBC film (Fig. 3d) is not reflected in the UV–vis measurements: **the transmittance at 550 nm is 84.02% for the free-standing MBC6D film and 84.44% for the MBC glass**, indicating no significant difference in intrinsic optical transparency.

The free-standing films shown in Fig. 3d were rolled or folded to demonstrate their flexibility and processability. In these configurations, multiple film–air interfaces are introduced, and the large refractive-index contrast between air and the film enhances light scattering and reflection, making the films appear less transparent. The apparent transparency in photographs is also affected by imaging conditions such as viewing angle and illumination; for example, the same

MBC glass appears visually different in Fig. 5c and Fig. 5d despite being the identical sample.

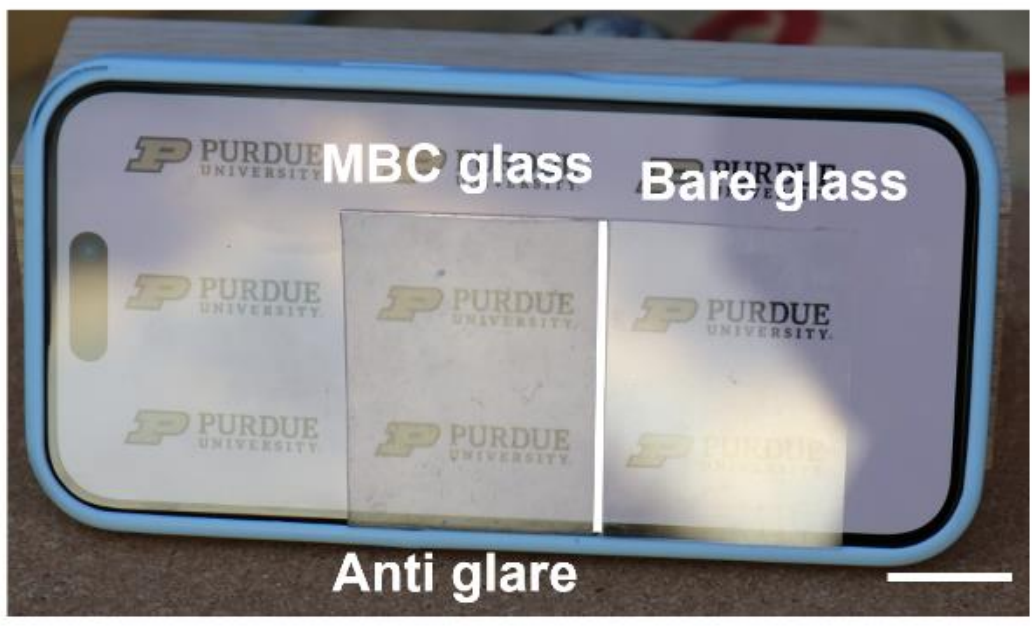

Fig. 5c. Outdoor anti-glare performance of MBC glass and bare glass on the screen of smart phone.

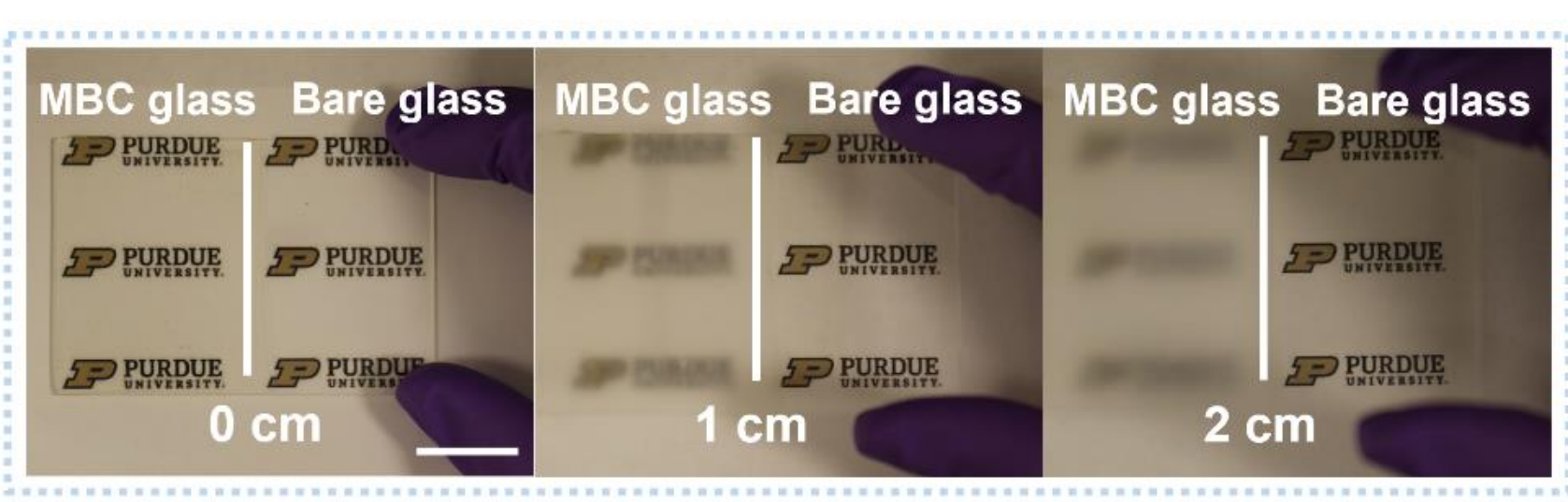

Fig. 5d. Photographs showing the view of pattern under MBC glass and bare glass at different viewing distances (0 cm, 1 cm, 2 cm).

We have further added a visibility test for the free-standing MBC film. As shown in Supplementary Fig. 20c, when the free-standing MBC film was placed in direct contact with the patterned substrate (0 cm), the pattern remained clearly visible, demonstrating display performance comparable to that of the MBC glass.

Although the UV–vis measurements indicate only a minor difference in transmittance, **the pattern beneath the MBC glass appears slightly clearer.** We attribute this small difference to the different drying conditions: during drying on glass, **the hydroxyl-rich MBC network can interact with silanol groups on the glass** surface through hydrogen bonding, promoting intimate interfacial contact and restricting drying-induced deformation, resulting in a flatter film. In contrast, the free-standing films were dried on a polytetrafluoroethylene (PTFE) substrate, where weaker interfacial interactions allowed less constrained deformation during drying, which may reduce the overall flatness of the film and slightly decrease its apparent transparency. We have added this comparison to the manuscript as detailed below.

Compared with the free-standing MBC film, the MBC glass shows better visibility, which may be attributed to hydrogen-bond-assisted adhesion between the coating and the glass substrate that restricts drying-induced deformation and results in a flatter surface morphology[54]. (Page 17, Line 356)

We have also revised the Methods section to provide additional details regarding the drying conditions.

Finally, the hydrogels were air-dried on a polytetrafluoroethylene (PTFE) substrate at room temperature with a relative humidity of 30% for 24 h to obtain films. (Page 21, Line 441)

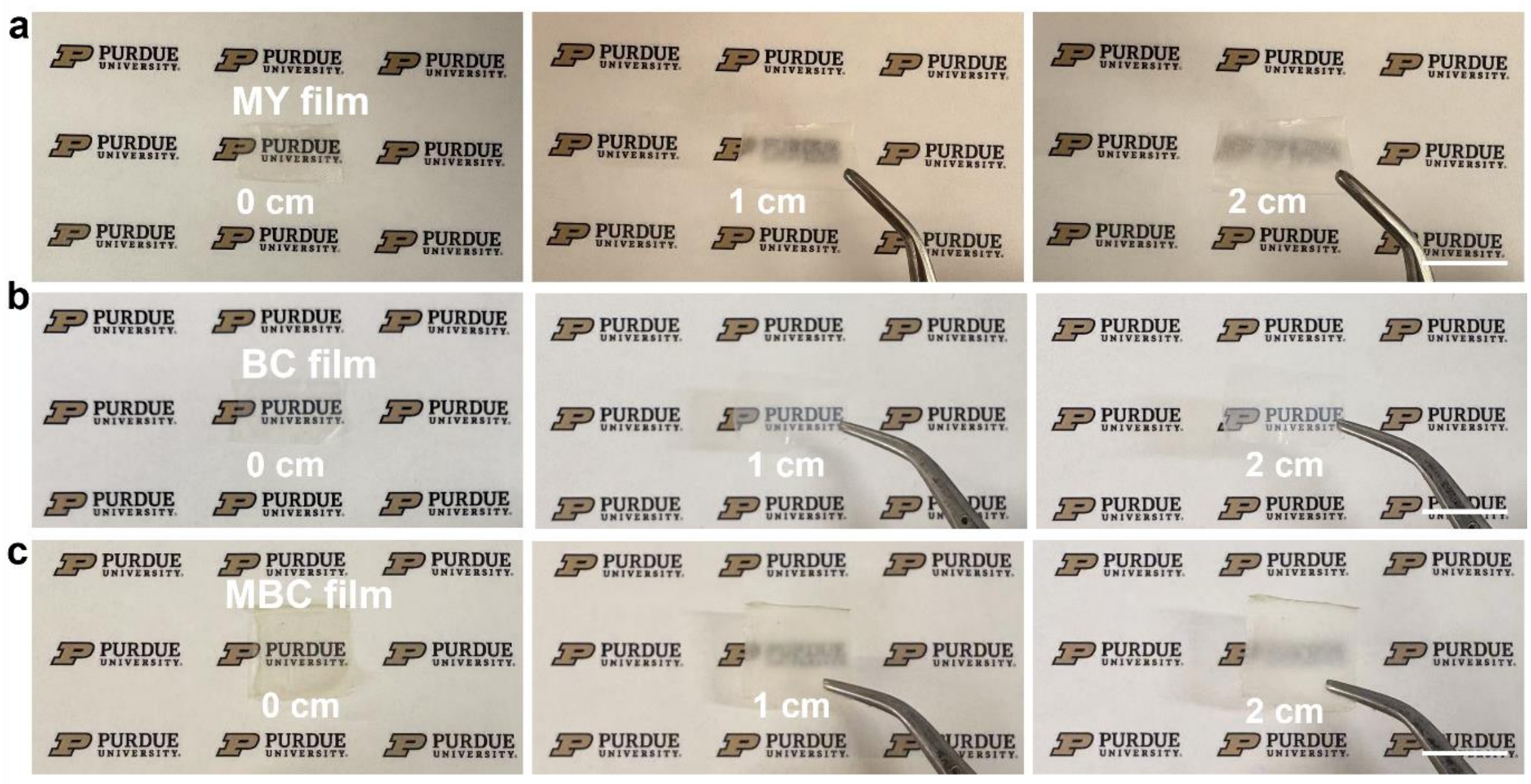

Supplementary Fig. 20. Pattern visibility tests. Photographs showing the view of pattern under a MY film, b BC film and c MBC film at different viewing distances (0 cm, 1 cm, 2 cm). Scale bar = 2 cm.

Comment 2: *The TGA graph presented in Fig 4f does not appear sufficient to definitively determine or calculate hyphal content based on height or pore size. The authors should consider revising the manuscript to reflect this limitation. Alternative approaches such as EDS analysis (which may allow a crude estimate based on nitrogen presence, given that chitin is nitrogen-*

rich) or through the use of specific cellulose or mycelium fluorescent dyes could provide more rigorous quantification of the relative contributions of each component.

Response: We thank the reviewer for this valuable comment, and we agree that the TGA results alone are not sufficient to definitively determine the hyphal content of the MBC films. Accordingly, we now describe the **TGA data only as indicating a compositional trend** rather than providing a quantitative measure, and we have explicitly acknowledged this limitation. We also note that, in the revised manuscript, Fig. 4f now shows the new EDS nitrogen-content data, while the original TGA curves have been moved to Supplementary Fig. 15.

Thermogravimetric analysis (TGA) also provides supporting evidence for the trend of increased mycelial contribution indicated by the EDS analysis, **although it cannot directly quantify the mycelial content.** Pure BC films show the greatest weight loss (83.9%) between 200–550 °C, leaving only 8.2% residue at 550 °C (Supplementary Fig. 15a). The weight loss of MBC films between 200–550 °C decreases from 73.8% to 64.9%, while the char yield at 550 °C increases from 15.2% to 26.0% with prolonged culture duration (Supplementary Fig. 15b-d). The increased char yield is mainly attributed to the greater contribution of mycelial components, such as chitin, proteins, and lipids, which tend to form more residual char during thermal degradation[51,52]. (Page 14, Line 291)

As suggested by the reviewer, we also performed EDS analysis to estimate the relative mycelial contribution from the nitrogen signal, since chitin and other nitrogen-containing components are present in fungal hyphae whereas BC contains no nitrogen. The nitrogen contents of the pure BC and MY films were 0% and 7.1%, respectively, while the MBC samples cultured for 4, 6, and 8 days contained 2.1%, 3.4%, and 4.0% nitrogen, respectively, consistent with an increasing mycelial contribution at longer culture durations. All films used for EDS analysis were prepared from NaOH-treated hydrogels followed by thorough washing. **We agree that EDS cannot provide an absolute quantification of mycelial content, and we have acknowledged this limitation in the revised manuscript as detailed below.**

Energy-dispersive X-ray spectroscopy (EDS) was applied to evaluate changes in mycelial

content within the MBC films based on the nitrogen signal, since BC contains no nitrogen, whereas mycelium contains nitrogen-rich components such as chitin and proteins. As shown in Fig. 4f and Supplementary Fig. 16, the pure BC film exhibits no detectable nitrogen signal, while the pure MY film contains 7.1% nitrogen. In comparison, the MBC samples cultured for 4, 6 and 8 days show nitrogen contents of 2.1%, 3.4% and 4.0%, respectively. **Although EDS cannot accurately quantify the mycelial content, the increasing nitrogen signal suggests a greater mycelial contribution at longer culture durations.** (Page 13, Line 283)

We also added details in Methods section.

Energy-dispersive X-ray spectroscopy (EDS) was performed using a Helios G4 UX dual-beam system to evaluate the elemental composition of the films. All EDS spectra were collected at an accelerating voltage of 10 kV and a beam current of 0.1 nA. The relative nitrogen content was calculated by dividing the nitrogen atomic percentage by the sum of the carbon, nitrogen, and oxygen atomic percentages obtained from EDS analysis. All films used for EDS analysis were prepared from NaOH-treated hydrogels followed by thorough washing. (Page 21, Line 450)

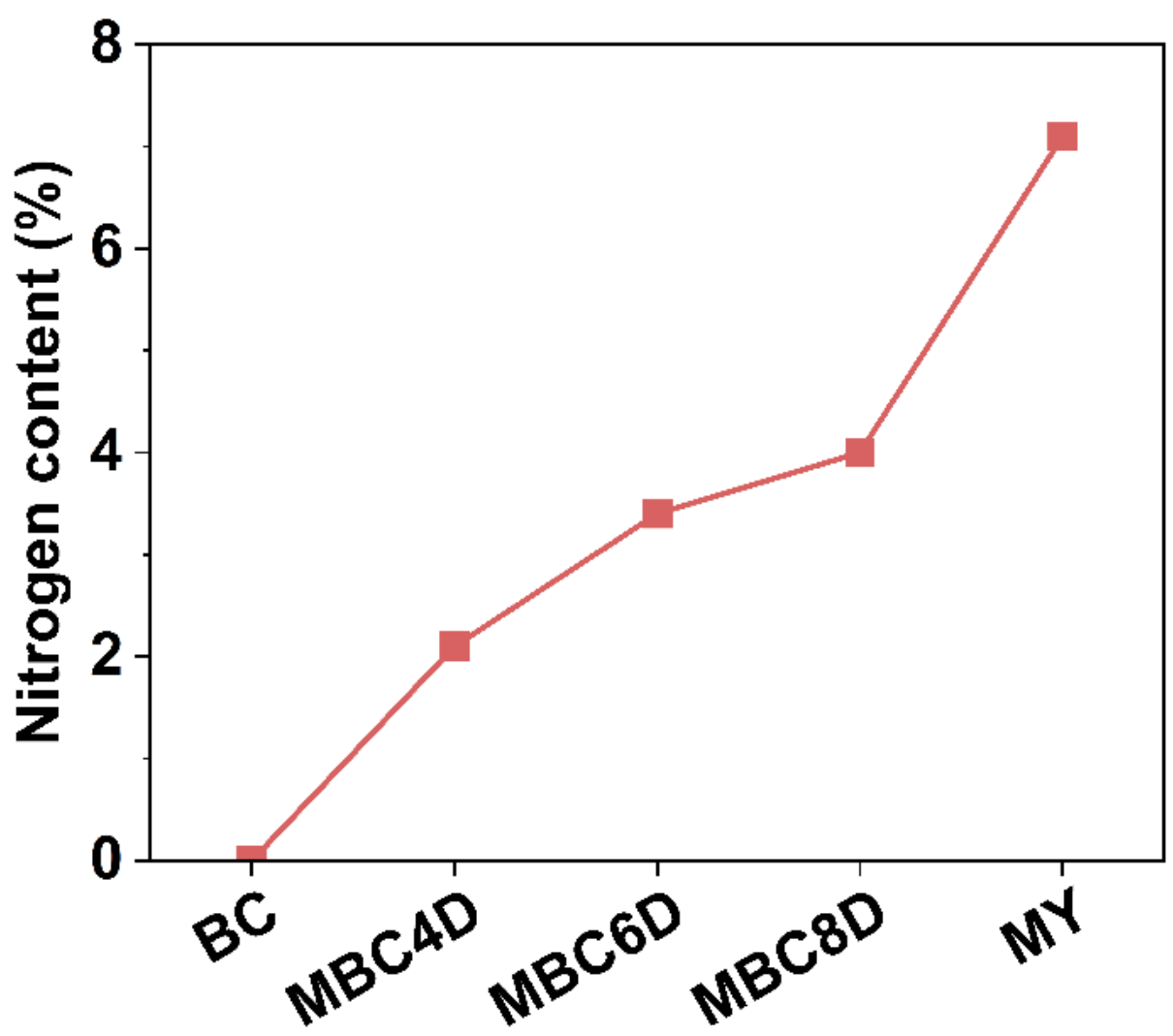

Fig. 4f. Nitrogen content of BC, MY, and MBC films with different culture durations determined by energy-dispersive X-ray spectroscopy (EDS). (MBC4D, 4 days; MBC6D, 6 days; MBC8D, 8 days).

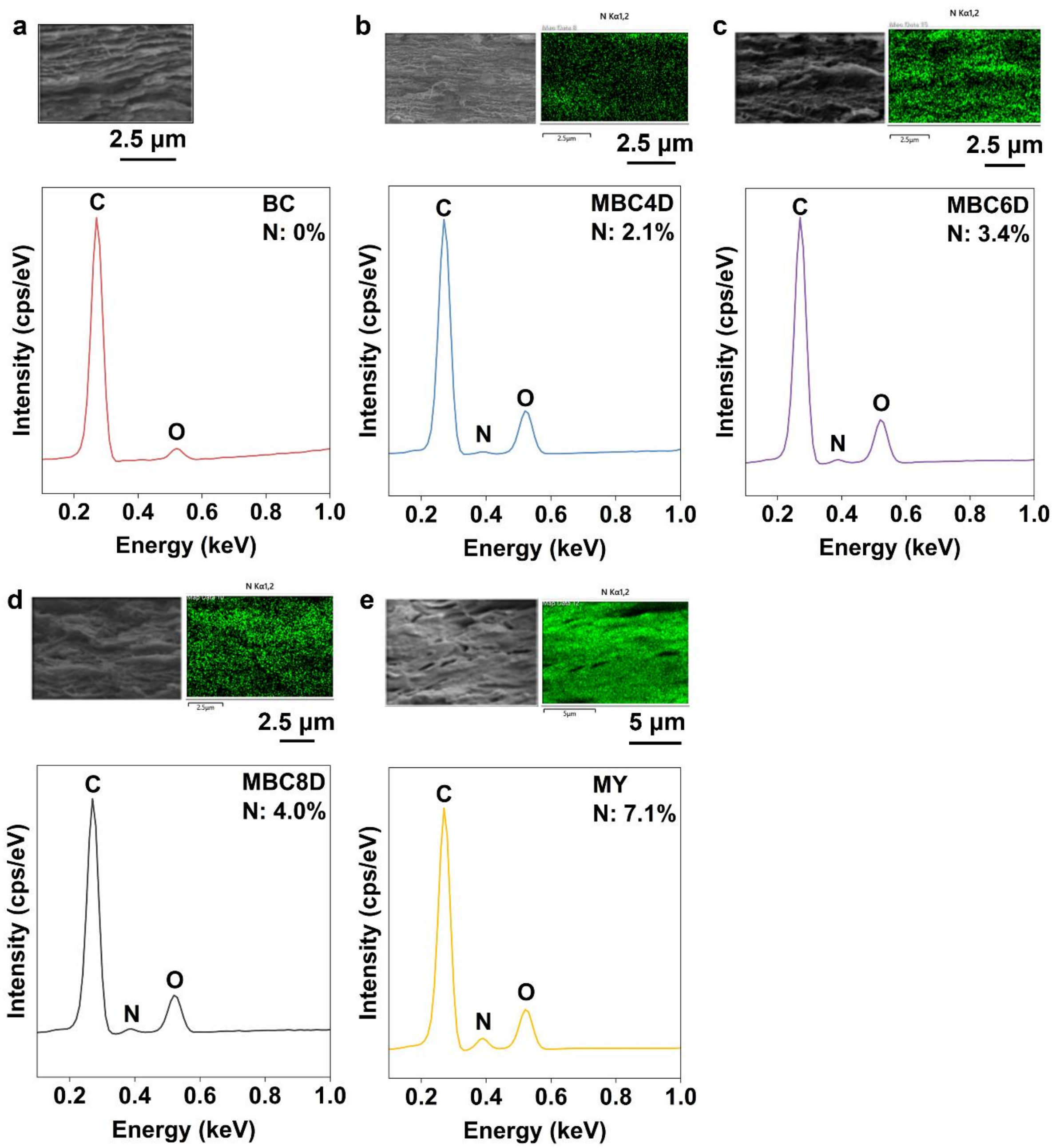

Supplementary Fig. 16. Energy-dispersive X-ray spectroscopy (EDS) analysis of BC, MY and MBC films with different culture durations. a–e SEM images, EDS nitrogen elemental maps, and EDS spectra of **a** BC, **b** MBC4D, **c** MBC6D, **d** MBC8D and **e** MY films.

Comment 3: *The text states that "MBC films with 4-day and 6-day culture durations exhibit only a few scattered hyphae, while the MBC8D sample shows relatively higher hyphal content." However, in Fig S15, the thickness measurements are very similar for the 6-day and 8-day samples, and the porosity measurements from Fig S16 show only modest differences. The*

authors should explain more rigorously how these structural measurements support the conclusion that 8-day cultivation results in significantly higher hyphal content. Furthermore, the ImageJ porosity analysis may reflect pore spaces caused by wrinkling during drying rather than definitively being caused by hyphal incorporation, and this limitation should be acknowledged.

Response: We appreciate this comment, and we agree that SEM observations alone cannot provide a reliable or quantitative determination of mycelial content. We have therefore **removed the statement** that inferred differences in hyphal content solely from SEM images. We also agree that the thickness variation measured from SEM images cannot serve as direct evidence of changes in mycelial content; the thickness data are now discussed only **in the context of the optical properties**, as shown below, and are no longer used to infer mycelial content.

The film thickness increases from 7.85 ± 0.22 μm at 4 days to 9.10 ± 0.28 μm at 6 days and 9.64 ± 0.44 μm at 8 days, which further promotes light scattering by extending the optical path length within the hierarchical network (Supplementary Fig. 18). (Page 14, Line 314)

We also agree that the ImageJ-based pore-area analysis cannot fully distinguish pores generated by hyphal incorporation from wrinkles formed during drying. We have therefore **revised the manuscript to acknowledge this limitation**, and we now treat the pore-area fraction as a structural feature associated with increased mycelial incorporation rather than as a direct quantification of mycelial content.

Although the presence of wrinkles may affect the accuracy of the porosity measurement, the analysis still provides a rough indication of the increasing pore content in the films. (Page 14, Line 308)

Accordingly, the revised text presents the change in pore-area fraction as a structural consequence of increased mycelial incorporation rather than as direct evidence of its absolute amount, as detailed below.

The increased mycelial hyphae with increasing culture duration introduces more interlamellar air pores into the MBC films. The estimated pore area fraction in the film cross-section increases from 5.02% to 11.85% with prolonged culture duration (Supplementary Fig. 17). Although the presence of wrinkles may affect the accuracy of the porosity measurement, the analysis still suggests a general increase in pore content within the films. The increased number of macropores and mycelial hyphae provides additional light-scattering interfaces due to the refractive-index (RI) mismatch among air, mycelium, and BC. (Page 14, Line 307)

To provide more direct evidence for the change in mycelial content, we added the EDS analysis described in our response to Minor Comment 2 (nitrogen contents of 2.1%, 3.4%, and 4.0% for the 4-, 6-, and 8-day MBC films, respectively), which supports an increasing mycelial contribution with culture duration while acknowledging that EDS cannot provide an absolute quantification.

Energy-dispersive X-ray spectroscopy (EDS) was applied to evaluate changes in mycelial content within the MBC films based on the nitrogen signal, since BC contains no nitrogen, whereas mycelium contains nitrogen-rich components such as chitin and proteins. As shown in Fig. 4f and Supplementary Fig. 16, the pure BC film exhibits no detectable nitrogen signal, while the pure MY film contains 7.1% nitrogen. In comparison, the MBC samples cultured for 4, 6 and 8 days show nitrogen contents of 2.1%, 3.4% and 4.0%, respectively. **Although EDS cannot accurately quantify the mycelial content, the increasing nitrogen signal suggests a greater mycelial contribution at longer culture durations.** (Page 13, Line 283)

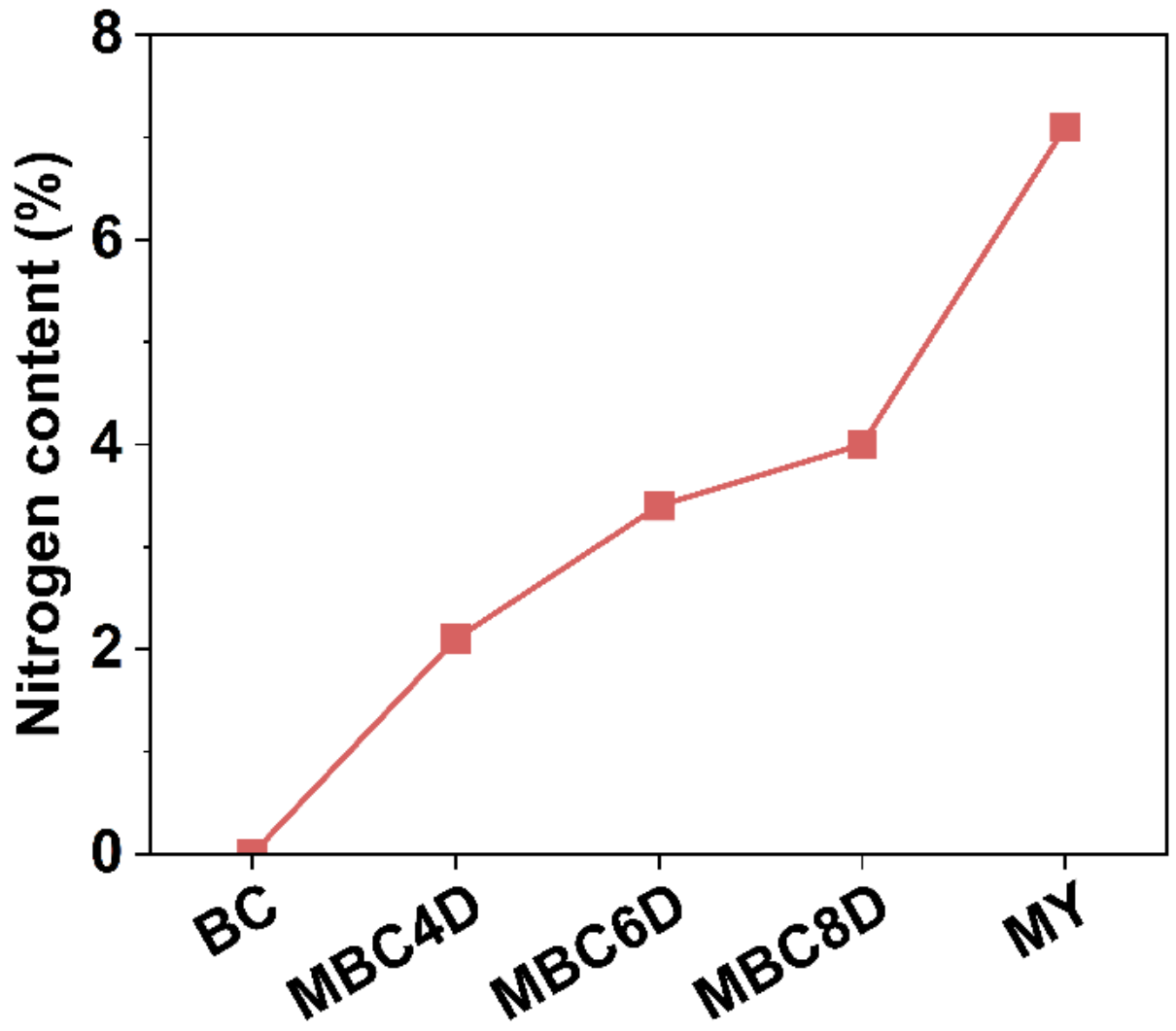

Fig. 4f. Nitrogen content of BC, MY, and MBC films with different culture durations determined by energy-dispersive X-ray spectroscopy (EDS). (MBC4D, 4 days; MBC6D, 6 days; MBC8D, 8 days).

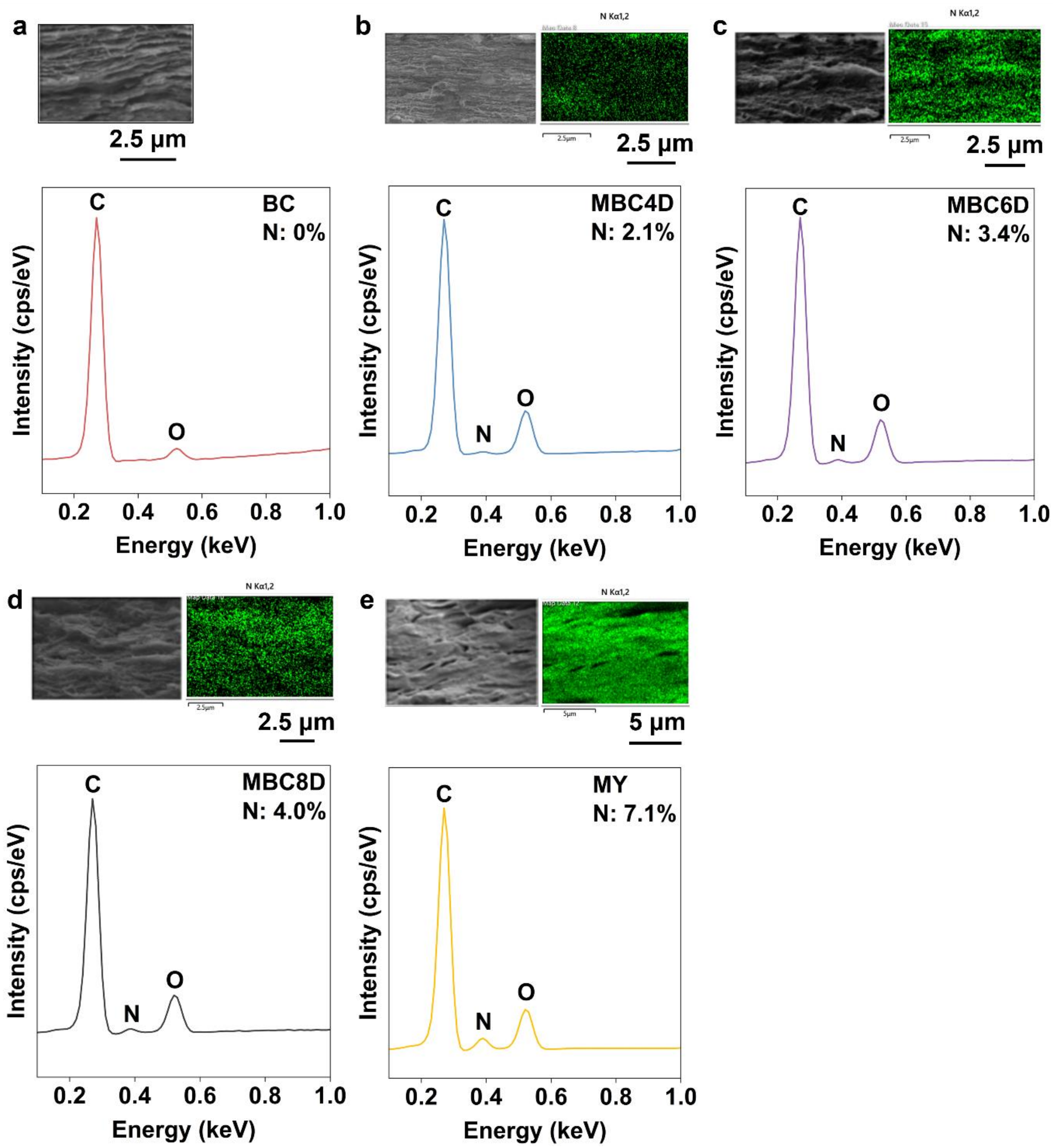

Supplementary Fig. 16. Energy-dispersive X-ray spectroscopy (EDS) analysis of BC, MY and MBC films with different culture durations. a–e SEM images, EDS nitrogen elemental maps, and EDS spectra of **a** BC, **b** MBC4D, **c** MBC6D, **d** MBC8D and **e** MY films.

Comment 4: *Fig 4f: It does not look possible to determine hyphal content from this TGA graph, nor is it possible to calculate this based on height or pore size. Could the authors consider revising the manuscript to reflect this limitation? An EDS analysis may allow a crude estimate based on nitrogen presence. Or through use of a specific cellulose or mycelium dye.*

Response: We thank the reviewer for this comment, which is closely related to Minor Comment 2. We agree that the hyphal content cannot be quantitatively determined from the TGA curves, film thickness, or pore-area fraction alone, and we have clarified that **these analyses are used to explain the tunability of the optical properties rather than to quantify hyphal content**.

Regarding the TGA analysis, previous studies have shown that the residual char is mainly associated with mycelial components such as chitin, proteins, and lipids, so the increased char yield with longer culture duration roughly reflects an increasing mycelial contribution. We have accordingly described the TGA results only as **indicating a compositional trend**, and have acknowledged the corresponding limitation in the revised manuscript.

Thermogravimetric analysis (TGA) also provides supporting evidence for the trend of increased mycelial contribution indicated by the EDS analysis, **although it cannot directly quantify the mycelial content.** Pure BC films show the greatest weight loss (83.9%) between 200–550 °C, leaving only 8.2% residue at 550 °C (Supplementary Fig. 15a). The weight loss of MBC films between 200–550 °C decreases from 73.8% to 64.9%, while the char yield at 550 °C increases from 15.2% to 26.0% with prolonged culture duration (Supplementary Fig. 15b-d). The increased char yield is mainly attributed to the greater contribution of mycelial components, such as chitin, proteins, and lipids, which tend to form more residual char during thermal degradation[51,52]. (Page 14, Line 291)

To further support the increased mycelial content, we added EDS analysis based on the nitrogen signal, since nitrogen is associated mainly with mycelial components rather than BC. The nitrogen contents of the pure BC and MY films were 0% and 7.1%, respectively, while the 4-, 6-, and 8-day MBC films contained 2.1%, 3.4%, and 4.0% nitrogen, respectively, supporting an increasing mycelial contribution. We agree that EDS cannot provide an absolute quantification of mycelial content, and we have acknowledged this limitation in the revised manuscript as detailed below.

Energy-dispersive X-ray spectroscopy (EDS) was applied to evaluate changes in mycelial content within the MBC films based on the nitrogen signal, since BC contains no nitrogen,

whereas mycelium contains nitrogen-rich components such as chitin and proteins. As shown in Fig. 4f and Supplementary Fig. 16, the pure BC film exhibits no detectable nitrogen signal, while the pure MY film contains 7.1% nitrogen. In comparison, the MBC samples cultured for 4, 6 and 8 days show nitrogen contents of 2.1%, 3.4% and 4.0%, respectively. **Although EDS cannot accurately quantify the mycelial content, the increasing nitrogen signal suggests a greater mycelial contribution at longer culture durations.** (Page 13, Line 283)

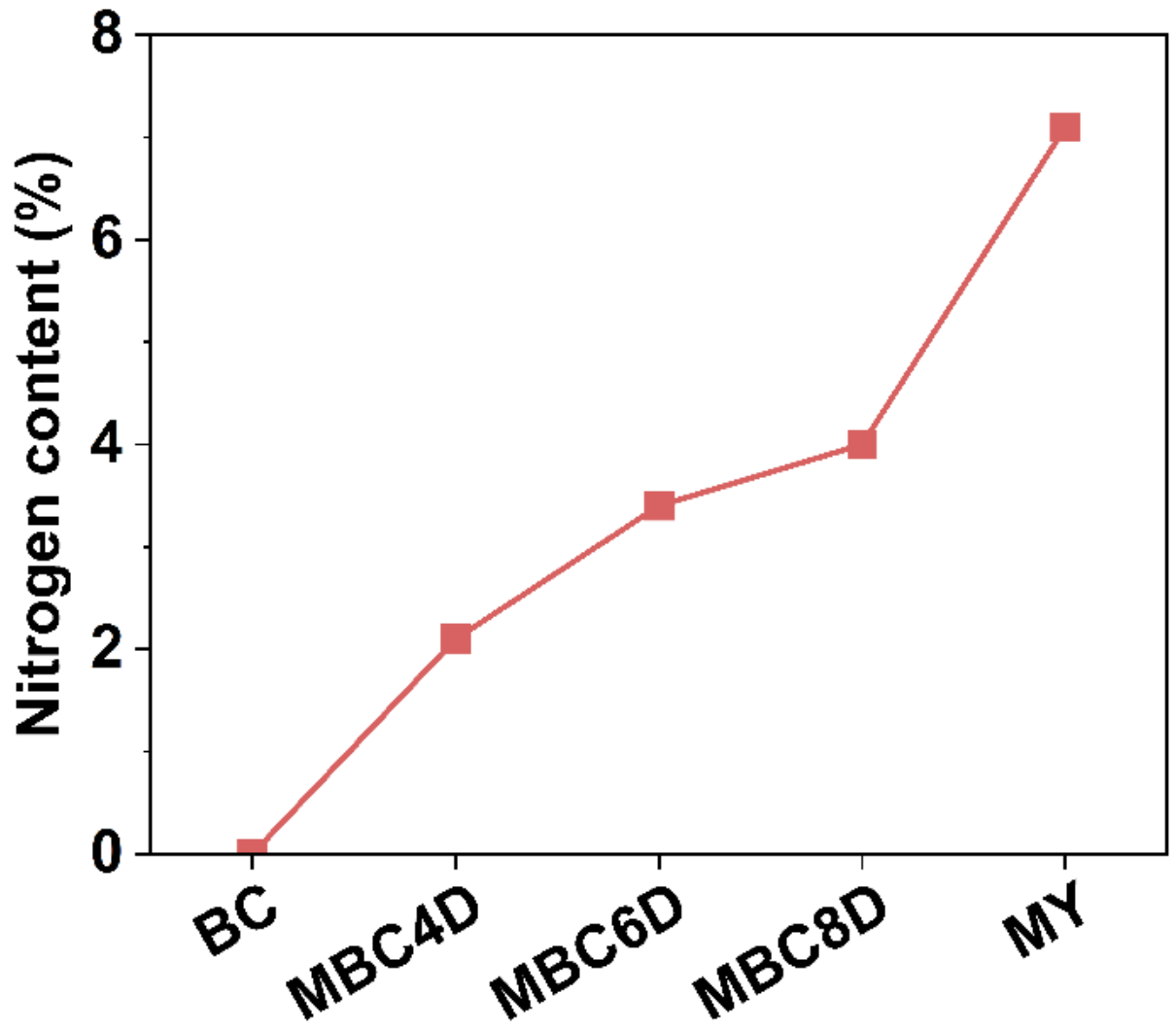

Fig. 4f. Nitrogen content of BC, MY, and MBC films with different culture durations determined by energy-dispersive X-ray spectroscopy (EDS). (MBC4D, 4 days; MBC6D, 6 days; MBC8D, 8 days).

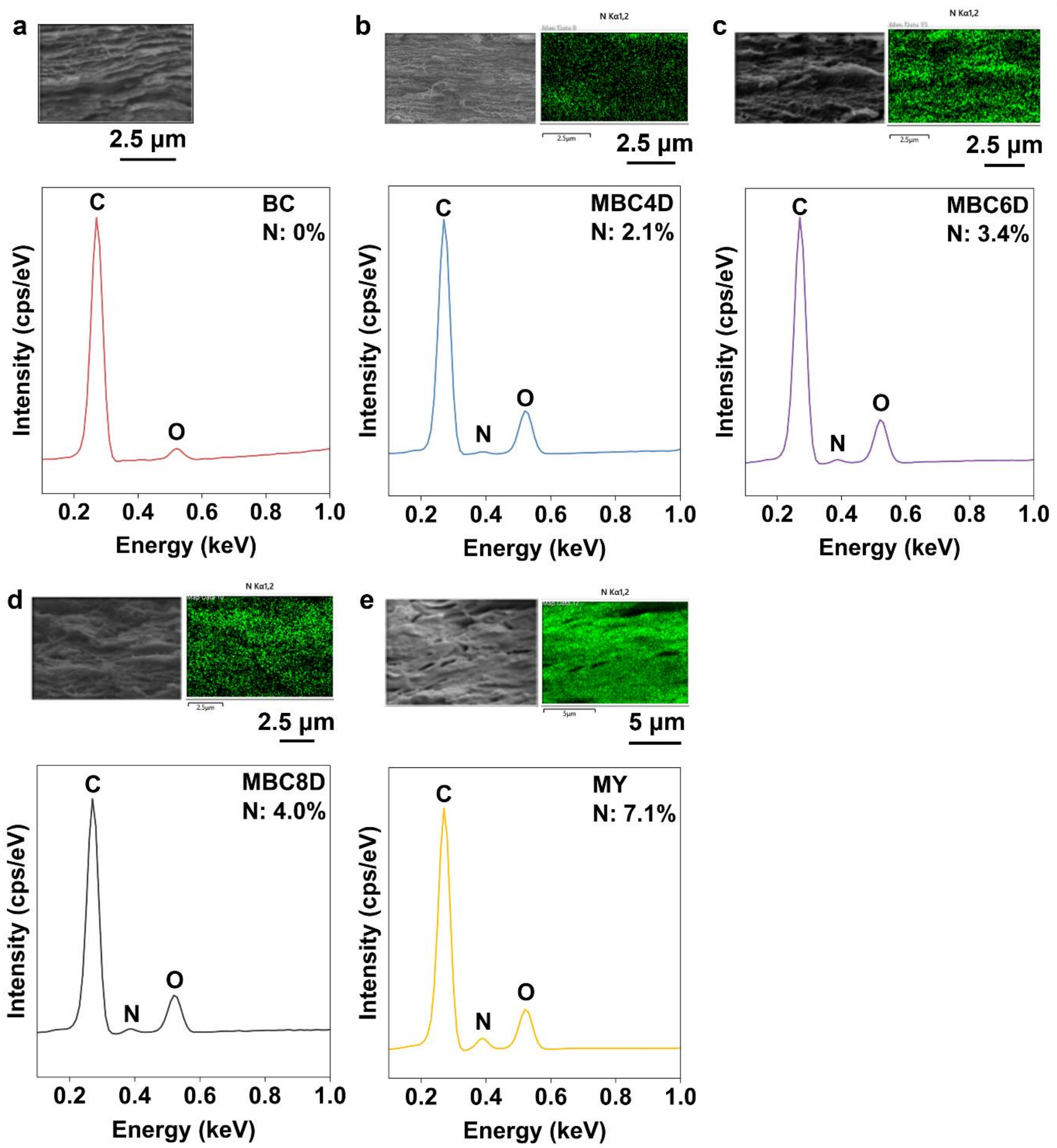

Supplementary Fig. 16. Energy-dispersive X-ray spectroscopy (EDS) analysis of BC, MY and MBC films with different culture durations. a–e SEM images, EDS nitrogen elemental maps, and EDS spectra of **a** BC, **b** MBC4D, **c** MBC6D, **d** MBC8D and **e** MY films.

Comment 5: *Line 162: Where is the evidence for hydrogen bond formation? There is still no evidence that the fibres shown are "new" fibres, specifically the BC.*

Response: We appreciate this comment. Several characterizations in this study, together with previous reports on hydrogen bonding in BC and in cellulose/chitin-based systems, support the

presence of hydrogen-bonding interactions between BC and mycelium in the MBC hydrogel.

Regarding *"the evidence for hydrogen bond formation"*

Previous studies have shown that BC possesses an extensive hydrogen-bonding network owing to its abundant hydroxyl groups[4,7], and that hydrogen-bonding interactions are also commonly observed between cellulose and other polysaccharides[8,9]. In our system, XPS and FTIR analyses showed that the mycelium surface bears **hydroxyl, amide, and carbonyl groups,** while BC is rich in **hydroxyl groups**, **providing abundant potential hydrogen-bond donors and acceptors at the interface**. Furthermore, the FTIR spectra showed a shift of the O–H stretching vibration toward lower wavenumbers in the MBC samples relative to pure BC, which is commonly regarded as indicative of strengthened hydrogen bonding. SEM observations also revealed intimate interfacial contact and intertwined assembly between the nanoscale BC fibers and the microscale mycelial hyphae, supporting a strong interfacial association consistent with hydrogen-bond-mediated interactions.

Regarding *"evidence that the fibres shown are "new" fibres"*

As shown in the magnified Fig. 3b, SEM imaging was performed at the healed fracture region after self-healing. Because the original MBC hydrogel was cut before healing, the observed crack-bridging region represents newly regenerated material formed after fracture rather than the original intact network. At this early healing stage, both regenerated hyphae and BC can be observed at the fracture interface. A nanoscale fibrillar network was visible within the healed region, morphologically distinct from the microscale hyphae owing to its much smaller fiber diameter. A similar hierarchical fibrillar morphology was also observed in Fig. 2j. In addition, Supplementary Fig. 8 shows that the newly regenerated region contained both hyphae and nanoscale fibrillar structures. We therefore attribute these nanofiber networks to newly formed BC generated during the healing process.

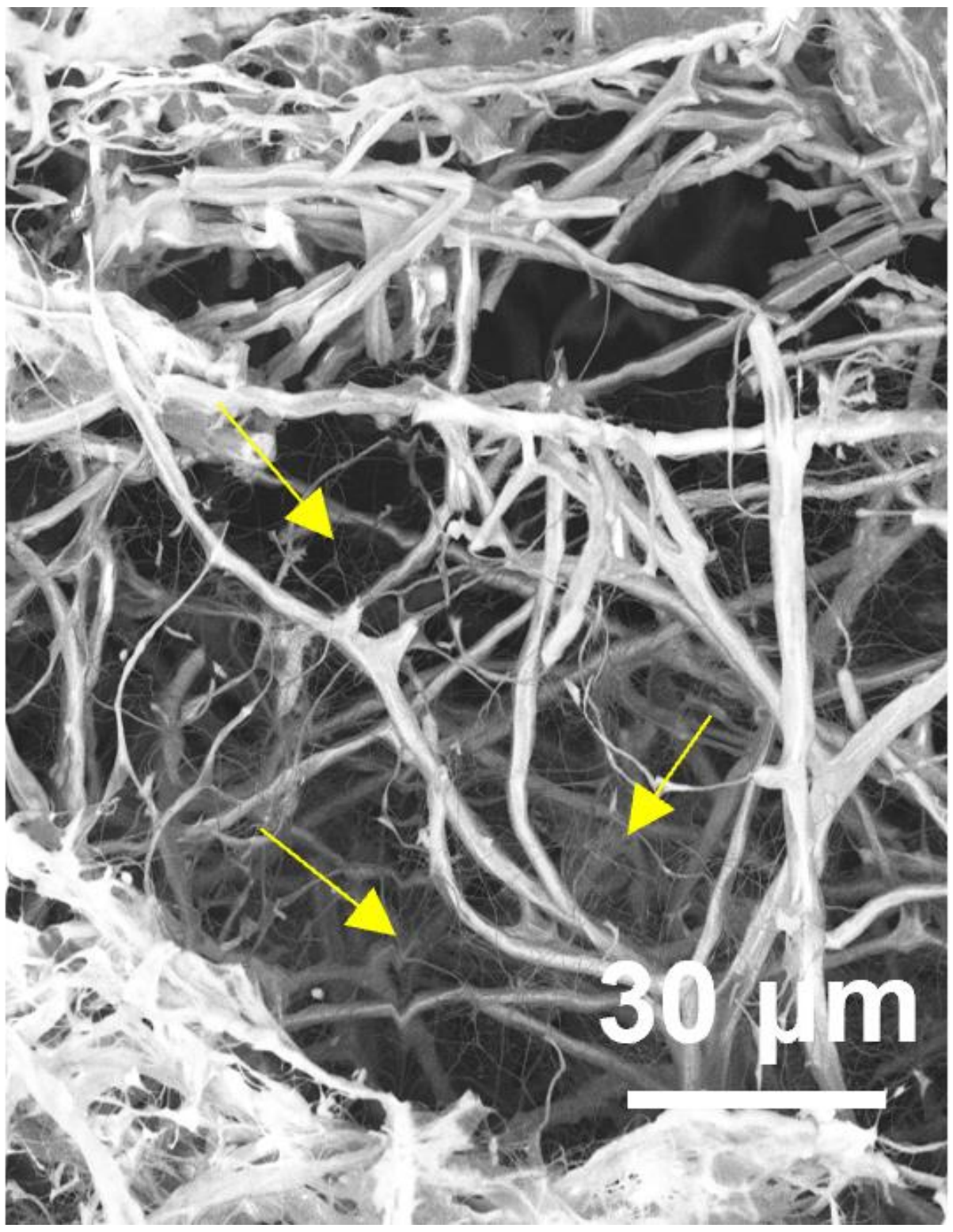

Fig. 3b. SEM images of the breakage after self-healing.

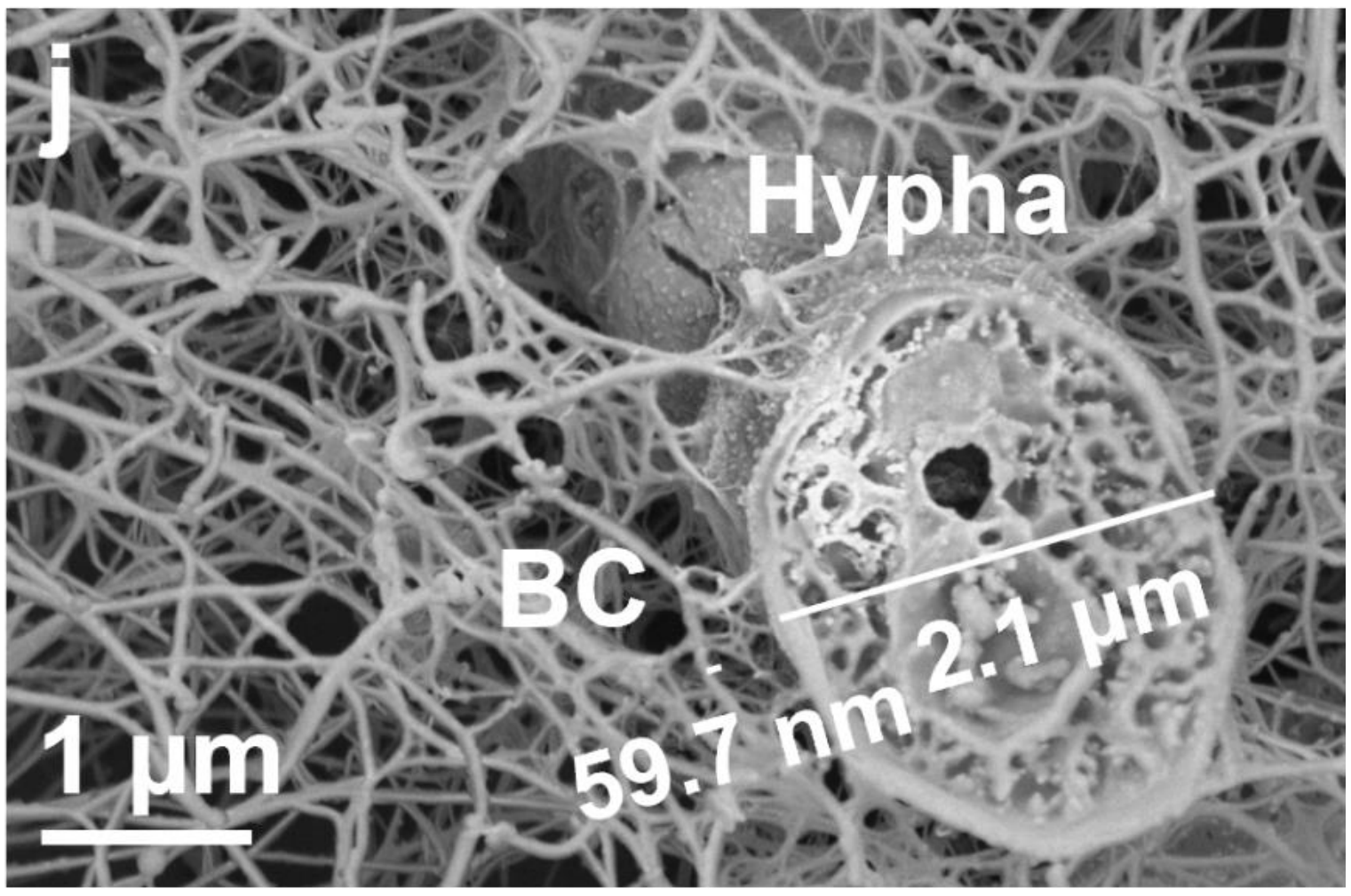

Fig. 2j. Cross-sectional cryo-SEM image of MBC hydrogel.

\

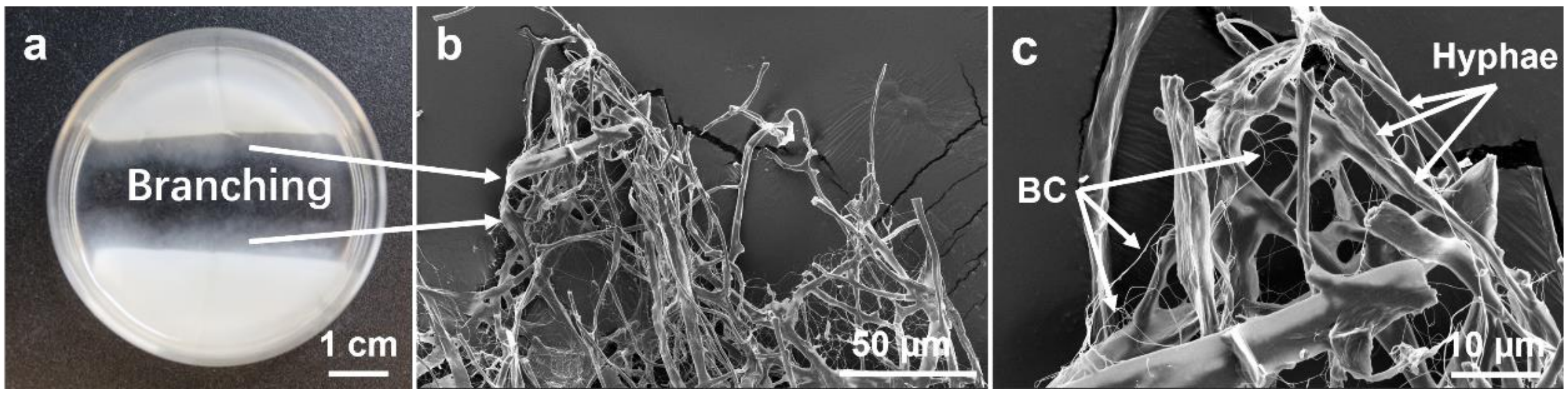

Supplementary Fig. 8. Self-healing and re-growth of MBC hydrogels. **a** Photographs showing the self-healing and re-growth of MBC hydrogel after being cut for 36 h. **b, c** SEM images of the newly grown thin layer composed of hierarchical BC nanofibers and hyphae.

Comment 6: *The HS medium as described in the manuscript appears to be prepared at half strength compared to the standard Hestrin-Schramm formulation (Glucose: 20 g/L, Yeast Extract: 5 g/L, Peptone: 5 g/L, Disodium Phosphate: 2.7 g/L, Citric Acid: 1.15 g/L). The authors should clarify whether this was intentional and provide justification. Additionally, it should be stated whether the medium was titrated to a specific pH prior to inoculation, as pH has a well-known effect on both BC production and fungal growth.*

Response: We appreciate the reviewer's careful comment. The HS medium composition was stated incorrectly in the original manuscript owing to an oversight, for which we apologize. The HS medium actually used in this study contained 20 g L^{-1} glucose, 5 g L^{-1} yeast extract, 5 g L^{-1} peptone, 2.7 g L^{-1} disodium phosphate (anhydrous), and 1.5 g L^{-1} citric acid monohydrate, following the formulation reported by Wang et al. (Adv. Mater. 2017, "Super-Strong, Super-Stiff Macrofibers with Aligned, Long Bacterial Cellulose Nanofibers")[10]. No pH adjustment was performed prior to inoculation. We have corrected the Methods section accordingly, as detailed below.

The BC hydrogel was prepared by inoculating 3 mL of bacterial pre-culture into 10 mL of HS medium containing 20 g L^{-1} glucose, 5 g L^{-1} yeast extract, 5 g L^{-1} peptone, 2.7 g L^{-1} disodium phosphate anhydrous and 1.5 g L^{-1} citric acid monohydrate. (Page 21, Line 429)

Comment 7: *The methods for the self-healing experiments are insufficiently described. The authors should clarify: how the samples were fractured; whether and how the samples were washed prior to rejoining; how long the samples were grown before fracture; and how long the healing period was before assessment. These details are essential for reproducibility and for interpreting the self-healing results.*

Response: We appreciate the reviewer's comment.

Regarding *"how the samples were fractured"*

The sample was removed from the liquid medium and directly cut into two pieces using a sterilized blade.

Regarding *"whether and how the samples were washed prior to rejoining"*

No washing step was performed before rejoining, in order to preserve the living cells and nutrients within the hydrogel.

Regarding *"how long the samples were grown before fracture"*

The MBC hydrogel sample was cultured for 8 days before the healing test.

Regarding *"how long the healing period was before assessment"*

After 12 h of healing, the two cut pieces had reconnected into an integrated hydrogel. After another 24 h of healing, the sample was freeze-dried, and the healed fracture region was observed by SEM.

We have added the detailed procedure for the self-healing experiment in the revised Methods section as detailed below.

After 8 days of culture, the MBC hydrogel sample was removed from the liquid medium and directly cut into two pieces using a sterilized blade. No washing step was performed before rejoining to preserve the living cells and nutrients within the hydrogel. The two cut pieces were immediately brought back into contact and placed in a sterilized petri dish. After 12 h of healing,

the two cut pieces had reconnected into an integrated hydrogel. After another 24 h of healing, the sample was freeze-dried, and the healed fracture region was observed by SEM. (Page 23, Line 494)

We have also clarified in the Results section as detailed below.

SEM image of the fractured region after an additional 24 h of healing shows that the continued growth of hyphae and BC forms new entanglements, further contributing to the repair of the damaged region[41]. (Page 9, Line 170)

Reviewer #3 (Remarks to the Author):

I co-reviewed this manuscript with one of the reviewers who provided the listed reports. This is part of the Nature Communications initiative to facilitate training in peer review and to provide appropriate recognition for Early Career Researchers who co-review manuscripts.

Response: We sincerely thank Reviewer #3 for co-reviewing this manuscript, and we greatly appreciate the time and effort devoted to evaluating our work. We have carefully considered all the comments raised during the review process and have made the corresponding revisions to strengthen the manuscript.

References

1. Yoshida, M., Igarashi, K., Kawai, R., Aida, K. & Samejima, M. Differential transcription of β-glucosidase and cellobiose dehydrogenase genes in cellulose degradation by the basidiomycete Phanerochaete chrysosporium. *FEMS Microbiol. Lett.* **235**, 177-182, (2004).
2. Okal, E. J., Aslam, M. M., Karanja, J. K. & Nyimbo, W. J. Mini review: Advances in understanding regulation of cellulase enzyme in white-rot basidiomycetes. *Microb. Pathog.* **147**, 104410, (2020).
3. Suzuki, H., Igarashi, K. & Samejima, M. Real-time quantitative analysis of carbon catabolite derepression of cellulolytic genes expressed in the basidiomycete Phanerochaete chrysosporium. *Appl. Microbiol. Biotechnol.* **80**, 99-106, (2008).
4. Yan, Y., Liu, L., Wang, F. & Zhang, H. Bacterial cellulose as a promising biodegradable bioplastic for sustainability. *Nat. Commun.* **17**, 4387, (2026).
5. Sun, J. *et al.* Enhanced mechanical energy conversion with selectively decayed wood. *Sci. Adv.* **7**, eabd9138.
6. Hastrup, A. C. S. *et al.* Differences in crystalline cellulose modification due to degradation by brown and white rot fungi. *Fungal Biol.* **116**, 1052-1063, (2012).
7. Saadi, M. A. S. R. *et al.* Flow-induced 2D nanomaterials intercalated aligned bacterial cellulose. *Nat. Commun.* **16**, 5825, (2025).
8. Wu, Y. *et al.* Revivable self-assembled supramolecular biomass fibrous framework for efficient microplastic removal. *Sci. Adv.* **10**, eadn8662, (2024).
9. Kong, P., Rosnan, S. M. & Enomae, T. Carboxymethyl cellulose–chitosan edible films for food packaging: A review of recent advances. *Carbohydr. Polym.* **346**, 122612, (2024).
10. Wang, S. *et al.* Super-Strong, Super-Stiff Macrofibers with Aligned, Long Bacterial Cellulose Nanofibers. *Adv. Mater.* **29**, 1702498, (2017).

Reviewer #2 (Remarks to the Author):

The authors have addressed most of my concerns. One minor change is required: the figure 4f should be a barplot rather than a line graph as there is no relationship across all the samples so the points shouldn't be joined up.

Response: We thank Reviewer #2 for the positive evaluation of our revised manuscript and for recognizing that the concerns raised in the previous review have been addressed. Figure 4f has been revised from a line graph to a bar plot, and the connecting lines between data points have been removed as shown below.

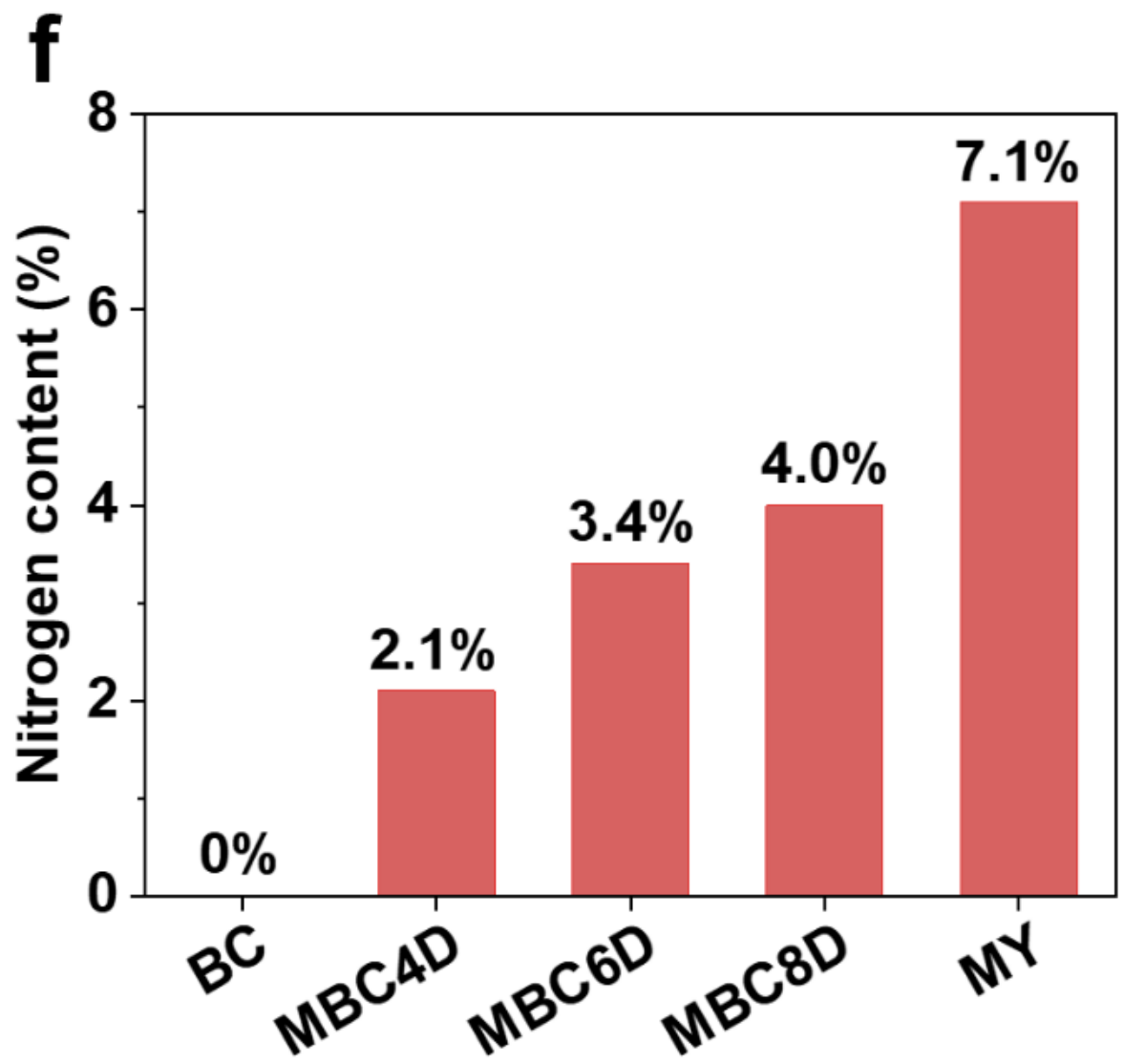

Fig. 4f. Nitrogen content of BC, MY, and MBC films with different culture durations determined by energy-dispersive X-ray spectroscopy (EDS). (MBC4D, 4 days; MBC6D, 6 days; MBC8D, 8 days).

Reviewer #3 (Remarks to the Author):

I co-reviewed this manuscript with one of the reviewers who provided the listed reports. This is part of the Nature Communications initiative to facilitate training in peer review and to provide appropriate recognition for Early Career Researchers who co-review manuscripts.

Response: We sincerely thank Reviewer #3 for co-reviewing this manuscript. We greatly appreciate the time and effort devoted to the evaluation of our work. We have carefully considered all comments provided during the review process and have made the corresponding revisions to strengthen the manuscript.
